# Mismatch between marine plankton range movements and the velocity of climate change

**DOI:** 10.1038/ncomms14434

**Published:** 2017-02-10

**Authors:** William J. Chivers, Anthony W. Walne, Graeme C. Hays

**Affiliations:** 1 Faculty of Science and IT, University of Newcastle, Ourimbah, New South Wales 2258, Australia; 2 Sir Alister Hardy Foundation for Ocean Science, The Laboratory, Citadel Hill, Plymouth PL1 2PB, UK; 3 Deakin University, Geelong, Australia, School of Life and Environmental Sciences, Centre for Integrative Ecology, Warrnambool, Victoria 3280, Australia

## Abstract

The response of marine plankton to climate change is of critical importance to the oceanic food web and fish stocks. We use a 60-year ocean basin-wide data set comprising >148,000 samples to reveal huge differences in range changes associated with climate change across 35 plankton taxa. While the range of dinoflagellates and copepods tended to closely track the velocity of climate change (the rate of isotherm movement), the range of the diatoms moved much more slowly. Differences in range shifts were up to 900 km in a recent warming period, with average velocities of range movement between 7 km per decade northwards for taxa exhibiting niche plasticity and 99 km per decade for taxa exhibiting niche conservatism. The differing responses of taxa to global warming will cause spatial restructuring of the plankton ecosystem with likely consequences for grazing pressures on phytoplankton and hence for biogeochemical cycling, higher trophic levels and biodiversity.

## Abstract (web summary)

Marine plankton are the basis of the oceanic food chain. Here, Chivers and colleagues use ocean-basin wide plankton population data over six decades to show huge differences in the response of different plankton groups to climate change with major implications for the marine ecosystem and fisheries.

Species distribution models that predict impacts of climate change on species' ranges generally assume that each species has a fixed environmental niche, referred to here as ‘niche conservatism', including an optimal temperature range so that each species' distribution and/or phenology will be determined by environmental conditions^1^. Certainly there is strong evidence that environmental conditions are an important driver of marine plankton distribution^2^,^3^,^4^,^5^,^6^,^7^. However, this assumption of environmental niche conservatism may be overly simplistic with palaeoecological and other studies indicating that species may be much more resilient to climate change than predicted by models assuming a fixed environmental niche^8^,^9^,^10^. Such resilience to climate change, referred to here as ‘niche plasticity', may be due to a range of biotic and abiotic factors including (a) evolutionary adaptation, (b) genetic variation across the range of a taxon, (c) phenotypic plasticity, (d) interaction with other taxa including competitors, parasites, prey and predators, each of which will exhibit their own responses to changing conditions or (e) phenological changes. Whether such niche plasticity is actually occurring in free-living populations remains equivocal, leading some authors to stress the need for more comprehensive estimates of climate change impacts especially on plankton^7^,^8^,^11^ due to their particular importance: their responses to climate change may have profound ecosystem consequences as they form an integral component of marine food chains and phytoplankton are responsible for nearly 50% of global photosynthesis^12^. In addition, they are a particularly tractable group for examining species responses to climate change.

While range shifts among marine plankton have been demonstrated in recent years^2^,^3^,^4^,^13^,^14^,^15^,^16^,^17^ it is unknown whether this group in general simply tracks environmental conditions, exhibiting thermal niche conservatism^7^,^18^,^19^, or displays resilience to climate change in some way and exhibits thermal niche plasticity. One method of examining responses of free-living plankton populations to environmental change is by assessing their range changes with respect to isotherm movement through time. Assessing the proportions of a plankton population north and south of individual isotherms is an approach to look for objective evidence for or against a fixed environmental niche: if a taxon is exhibiting niche plasticity in response to ocean warming then its range change should be less than the movement of its starting thermal niche and hence the proportion of the species polewards of each given isotherm should fall. We used data from the CPR^20^ (Continuous Plankton Recorder) survey, one of the most extensive biological time-series in existence, to map the distribution of 35 of the best sampled taxa of diatoms, dinoflagellates and copepods in the NE Atlantic and North Sea over six decades from 1954 to 2013. We assessed how plankton range changes compared to the velocity of climate change, that is, the rate at which isotherms have moved polewards, and hence we establish both the extent of variability across taxa in their range changes as well as whether taxa are showing environmental niche conservatism or plasticity.

While the range of some taxa closely tracked the velocity of climate change (that is, the rate of isotherm movement across years), for other taxa their range moved much more slowly, suggesting that their environmental niche has changed. These contrasting patterns of niche conservatism versus niche plasticity respectively varied across groups: dinoflagellates and copepods tended to show niche conservatism while diatoms tended to show niche plasticity, resulting in considerable differences in range shifts between taxa. This result contrasts with recent modelling and has major implications for the biological assemblage and hence the marine ecosystem and fisheries.

## Results

### Response of the major phytoplankton groups

**Figure 1:**
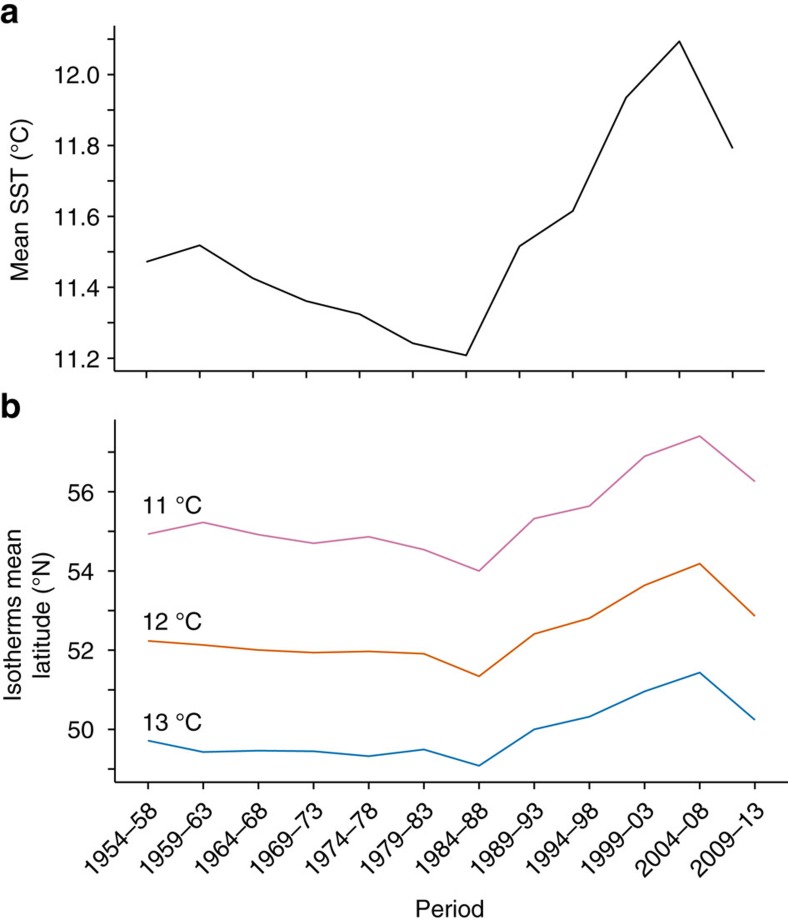
Sea surface temperatures and isotherm latitudes. (**a**) Mean of the estimated SST values over the twelve 5-year periods. (**b**) Mean latitudes of the 11, 12 and 13 °C isotherms for each 5-year period.

**Figure 2:**
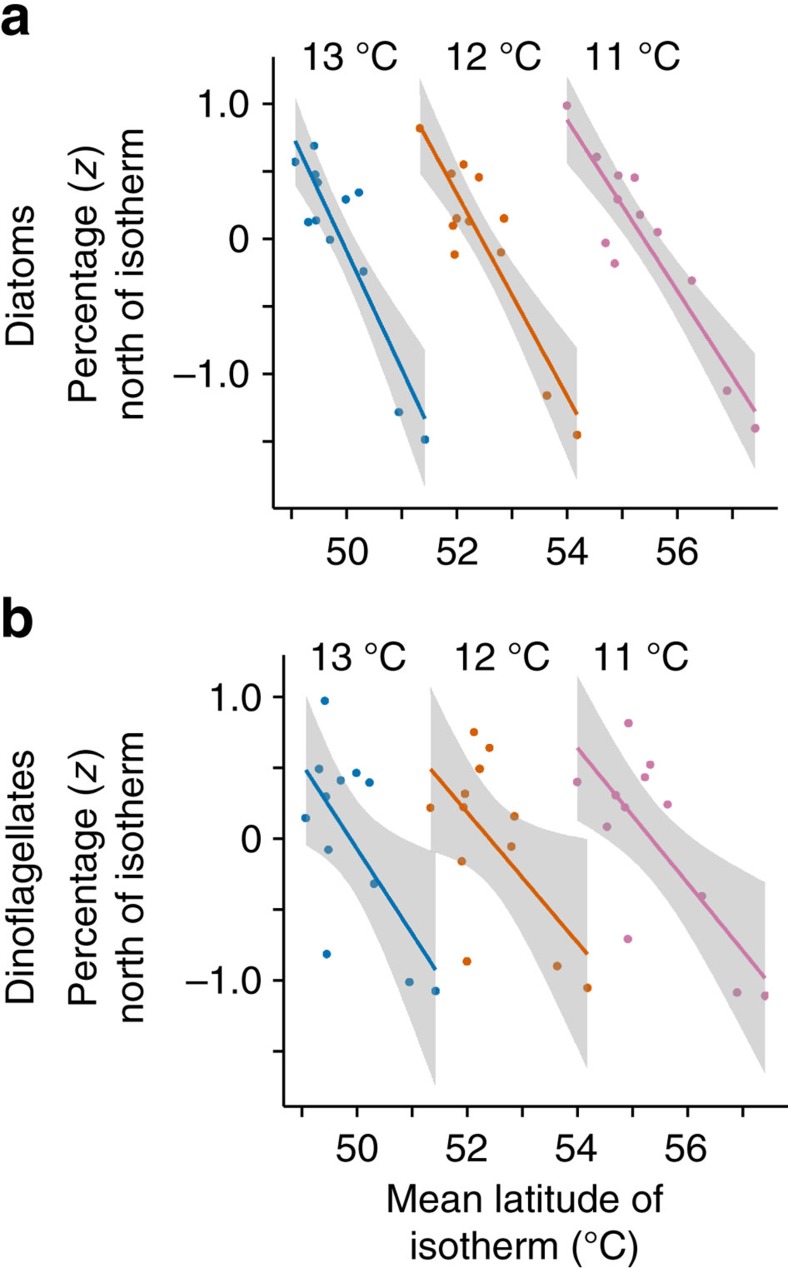
Proportions of diatom and dinoflagellate populations north of three isotherms. Proportions (normalized) of the (**a**) diatom and (**b**) dinoflagellate populations north of the three isotherms within the geographic area 45–64° N, 20° W–8° E in the twelve 5-year periods from 1954–1958 to 2009–2013. The isotherms move north as the SST rises; these plots illustrate negative correlations (at *P*<0.05) between the normalized population percentages north of each isotherm and the mean latitude of the isotherms. A loess smoother was used for locally weighted polynomial regression, the grey area indicating the 95% confidence interval for the line. The *P* values are listed in Supplementary Table 1a.

The six decades used here include periods of consistent ocean cooling (1959–1984) and warming (1984–2008) (Fig. 1a). The mean latitude of three isotherms at 11, 12 and 13 °C are correlated with the sea surface temperature (SST) (Fig. 1b, Supplementary Fig. 3), all three moving south in the period of cooling and then moving north in the period of warming. We found that diatoms and dinoflagellates, major phytoplankton groups, both broadly exhibited evidence for a plastic environmental niche: Fig. 2 illustrates the relationship between the percentage of each group north of each of the three isotherms and the latitude of those isotherms in the twelve 5-year periods 1954–2013. In all cases there was a significant negative correlation (Supplementary Table 1a): as the isotherms moved north the proportion of each group north of each isotherm fell and vice versa.

**Figure 3:**
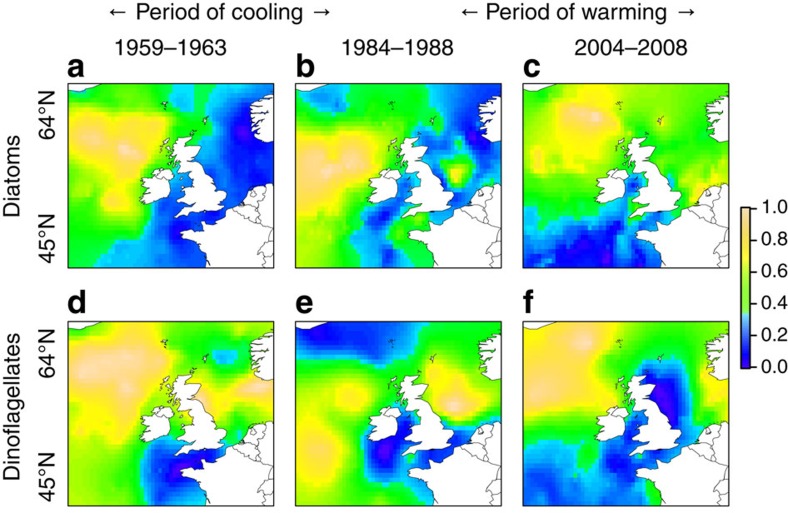
Movement of diatoms and dinoflagellates in cooling and warming periods. (**a–f**) Maps of the totals of the log-transformed (log(*x*+1)) cell counts determined by ordinary kriging for all diatom and dinoflagellate taxa. The maps were independently scaled 0.0–1.0 to highlight population movements. The movement of the dinoflagellates south in the period of cooling and then north in the period of warming exceeds the corresponding movement of the diatoms.

Despite this, both groups exhibited major range changes over recent decades: Fig. 3 maps the two groups in the 5-year periods at the start of the cooling period (1959–1963), the transition from cooling to warming (1984–1988) and the end of the warming period (2004–2008). During the period of cooling the median latitude of the diatoms moved south 84 km before moving north 92 km during the period of warming; the median latitude of the dinoflagellates moved south 111 km then north 135 km in the same periods. These range changes were smaller than the movement of the isotherms, that is, the velocity of climate change (Fig. 1, Table 1b, Supplementary Table 5).

### Differences revealed by individual taxa

Although the two groups appear to be exhibiting similar behaviour, analysis of the movements of the 35 individual taxa used here (mostly species) provided compelling evidence that more diatom taxa exhibit niche plasticity than do dinoflagellate taxa. Figure 4 and Supplementary Fig. 2 show the movements of each taxon in the periods of cooling and warming, Table 1a indicates significant negative correlations between proportion north of isotherms and isotherm latitudes for each taxon and Supplementary Figs 4–42 show further details for each taxon and group.

Ten of the twelve diatom taxa exhibited strong negative correlations between the percentage north of an isotherm and that isotherm position in periods of both warming and cooling (Table 1a), *Ditylum brightwellii* and *Skeletonema costatum* being the two diatom species not exhibiting such evidence of a plastic environmental niche. Only one of the diatom taxa, *Rhizosolenia styliformis*, exhibited a significant negative correlation between population size and mean SST (two others exhibited positive correlations, Table 1c, Supplementary Table 2a). The general observation is that diatoms appear able to adapt to SST changes and those SST changes do not negatively affect their abundance.

In contrast to the diatoms, only 4 of the 12 dinoflagellate taxa (*Ceratium fusus*, *C. minutum*, *Dinophysis spp.* and *Protoperidinium spp.*) exhibited environmental niche plasticity at all three isotherms examined (Table 1a). Of the dinoflagellate taxa which exhibited niche plasticity to SST at any isotherm, all exhibited negative correlations between population size and mean SST (Table 1c, Supplementary Table 2a) with the exception of *Ceratium minutum* which has a very low population size over the six decades examined here (Table 1d), resulting in a major decline in the abundance of dinoflagellates in the NE Atlantic region in the recent warming period, as has been noted previously in a shorter time-series^21^. The general observation is that dinoflagellates either showed no niche plasticity to SST changes or showed plasticity accompanied by falling populations and/or very low populations.

### Response of the copepods

Of the copepods, five species from the *Calanus*, *Euchaeta* and *Undeuchaeta* genera exhibited no evidence of niche plasticity (Table 1a, *E*. *acuta* in Figs 5 and 6, top row). The warm-water species *C. helgolandicus* exhibited population growth in response to warming while the cold-water species *C. finmarchicus* exhibited the opposite response (Table 1c). Of the six *Metridia* and *Pleuromamma* species four exhibited evidence of niche plasticity (*M. lucens*, *P. abdominalis*, *P. gracilis* and *P. robusta*) (Table 1a, *M*. *lucens* in Figs 5 and 6, bottom row) while two (*M. longa* and *P. borealis*), showed no such evidence. The two species in this group with the highest abundances, *M. lucens* and *P. robusta*, exhibited a negative correlation between population size and SST in the warming period despite exhibiting niche plasticity to SST (Table 1a,c). The general observation is that no *Calanus*, *Euchaeta* or *Undeuchaeta* genera exhibited niche plasticity and the *Metridia* and *Pleuromamma* species showing evidence of plasticity had very low and/or declining abundance.

The complexity of the response of these taxa is illustrated by Figs 5 and 6, which plot and map the responses of two copepod taxa, *E. acuta* and *M. lucens*. The former does not display niche plasticity: there was no correlation between proportion north of the isotherms and the latitude of the isotherm (Fig. 5a, Table 1a), meaning that the geographic range of the species moves with the isotherm (Fig. 6a–c): the species moved south 5 km in the period of cooling and then north 181 km in the period of warming (Table 1b). The species displays no correlation between abundance and SST (Fig. 5b, Table 1c).

In contrast, *M. lucens* displays niche plasticity: significant negative correlations between the proportion north of the isotherms and the latitude of the isotherm (Fig. 5c, Table 1a) were observed and the species unusually moved south in both the periods of cooling and warming, 87 and 38 km, respectively (Fig. 6d–f and Table 1b). The abundance of the species, however, fell in the period of cooling and has not recovered in the period of warming (Fig. 5d).

### Range movements in the warming period

**Figure 4:**
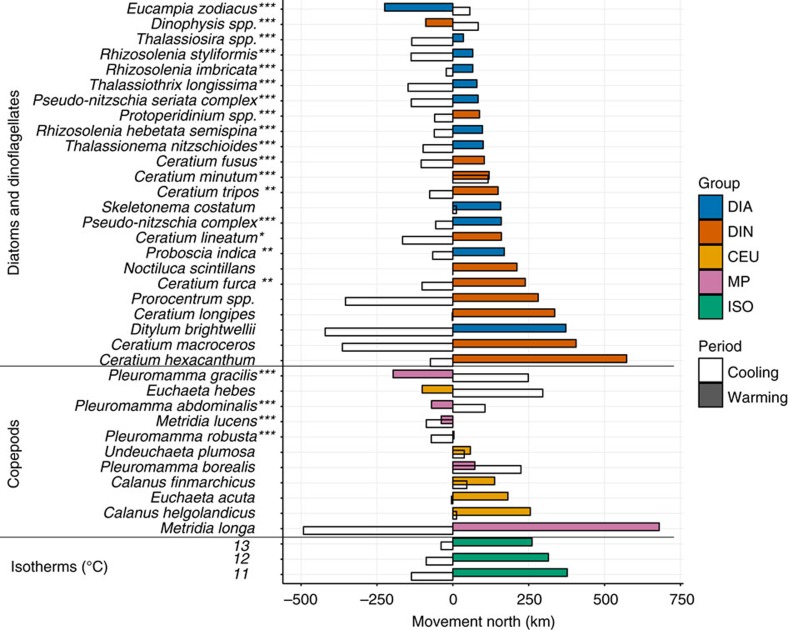
Movement of each taxon and isotherm in the cooling and warming periods. Movements in the cooling period of 1959–1984 are indicated with no colours, movements in the warming period of 1984–2008 are indicated with solid colours. The zero position on the *x* axis is the starting position of the range median latitude of each taxon at the start of each period. *Metridia longa*, for example, moved south 492 km in the cooling period then north 680 km in the warming period to move 188 km north overall in the two periods. In contrast *Eucampia zodiacus* moved north 56 km in the cooling period then south 225 km in the warming period to move 169 km south overall in the two periods. The one, two or three asterisks denote significant negative correlations (*P*<0.05) between the percentages of the populations north of one, two or three isotherms and the mean latitude of the isotherms in the six decades 1954–2013. A significant negative correlation indicates niche plasticity in relation to thermal change. CEU, *Calanus*, *Euchaeta* and *Undeuchaeta*; DIA, diatoms; DIN, dinoflagellates; ISO, isotherms; MP, *Metridia* and *Pleuromamma*.

The taxa examined here exhibiting niche conservatism (from all groups) showed a mean northward range shift in the warming period of 99 km per decade, contrasting with a 7 km per decade shift for those taxa exhibiting niche plasticity at all three isotherms (derived from Table 1a,b). The mean poleward movement in the warming period for all taxa analysed here was 54 km per decade while the mean latitude shifts for the isotherms at 11, 12 and 13 °C in the same period were 151, 126 and 104 km per decade, respectively. In the period of warming in the geographic area used here we found differences of up to 900 km in the movement of the median range latitude of individual taxa: the diatom *Eucampia zodiacus* exhibited a southerly movement of >220 km contrasting with the northerly movement of >680 km of the copepod *Metridia longa* (Fig. 4, Table 1b). While these two species exhibited the largest southerly and northerly range shifts of the taxa investigated here they are not extreme outliers: three taxa exhibited southerly movement of more than 100 km and 18 taxa exhibited a northerly movement of greater than 100 km in that period (Fig. 4, Table 1b).

### Analysis of two potential confounding factors

Two of the possible explanations for the lack of range shifts found here among some taxa, particularly the diatoms, are (i) phenological changes, whereby taxa adjust their seasonal timing of maximum abundance so that they continue to experience the same thermal regime even when sea temperatures are warming or cooling, and (ii) the relative positions of the taxon ranges and the latitudes of the isotherms used, whereby the extent of range movement depends on whether the range centre or range limits for a taxon are considered. To consider these possible explanations, we examined phenological shifts and the positions of the taxon ranges with respect to the isotherm latitudes over the period of warming and the six decades respectively. During the recent warming (1984–2008), there was no link between the extent of range movement for individual taxa and their shift in phenology (Fig. 7), that is, taxa that showed a limited northerly range shift in the recent warming era did not have a stronger tendency to shift their phenological timing of abundance to earlier in the year. Note however that generally across taxa, regardless of their range change, there was a tendency for a phenological shift to earlier in the year. The extent of range movement across taxa seemed unrelated to whether isotherms were examined at the range centre or range limits. First, for example, we found similar patterns of range movement with respect to different isotherms that occurred in different parts of the range of each taxon. Second, the extent of range movement seemed unrelated to whether taxa occurred largely to the south of the isotherms considered (for example, the copepod *Undeuchaeta plumosa*), to the north (for example, the copepod *Calanus finmarchicus* and the diatom *Skeletonema costatum*) or straddled the isotherms (the majority of taxa) (Supplementary Figs 4a–42a).

## Discussion

Profound temperature changes have been widely reported in the oceans, including the cooling and warming periods in the NE Atlantic and the cooling since 2008, which may be due to a reduction in the Atlantic meridional overturning circulation, melting of the Greenland ice sheet and/or solar variability, although the causes are not clear and are debated by various authors^22^,^23^,^24^,^25^,^26^. Using the periods of cooling and warming, the observation that the major phytoplankton groups and indeed the majority of individual taxa that moved north in periods of warming exhibited the opposite response in periods of cooling over six decades reduces the possibility of a driver other than thermal change. This conclusion is consistent with broad evidence for the role of temperature in determining plankton species ranges^2^.

The observation that most of the taxa reported here exhibited movement in the direction of the climate velocity, including many of the taxa exhibiting niche plasticity, is consistent with the predictions of Parmesan^6^ that it is more likely a species will move its range than evolve without range movement, the suggestion by Thomas *et al*.^7^ that taxa will move poleward with rising temperatures, and the finding of Pinsky *et al*.^17^ that over 70% of individual marine taxa changed latitude in the same direction as the local climate velocity. Our observations also support the conclusions from a recent examination of a 15-year plankton time-series at a fixed site in the Caribbean which suggested that some planktonic taxa can to adapt to changing temperatures by changing their environmental niche^1^. However, while our findings are consistent with these previous conclusions, they also show the complexity of the response in the biological assemblage to warming with widely different range changes occurring across taxa.

There are several possible explanations for the complex differences in plankton range movements reported here. First, genetic adaptation or genetic variation within one taxon across its range. Different taxa may show different levels of evolutionary change over these timescales or genetic differences over these geographic areas. While experimental manipulations have shown the ability of the coccolithophore *Emiliania huxleyi* to evolve over time-scales of a year^9^,^10^, with changes to the dominant genotype after exposure to changing temperature or pH, for this species one year represents many hundreds of generations. For larger metazoan taxa such as copepods which may have only a few generations per year, evolutionary change may not be as evident over a few decades compared with phytoplankton where generation times are much shorter. Second, phenotypic plasticity. Our observation that some taxa show minimal range changes in the face of rising ocean temperatures may reflect their phenotypic plasticity, also sometimes referred to as phenotypic buffering^8^. Such phenotypic plasticity is well known in terms of photoacclimation whereby individual phytoplankton cells can respond to a changing light environment^8^,^27^ but might equally occur with respect to changing temperature. Third, biotic interactions. While a taxon may be physiologically able to tolerate the abiotic conditions in a region, population of that region may not be viable because of the presence or absence of predators, prey, parasites or competitors^28^, each of which will themselves have different sensitivity and response to the biotic and abiotic changes. The chaotic nature of these interactions and their sensitivity to initial conditions make prediction of outcomes very difficult^29^. Fourth, phenological change. Taxa may show phenological responses to warming, changing their seasonal timing of maximal abundance^14^. In this way it is possible that when sea temperatures change species might remain in the same location, shifting their phenology rather than their range. Our analysis, however, suggests that the lack of movement north for the diatoms cannot be explained by a shift in the timing of the seasonal peak of abundance because this group did not exhibit any correlation between phenological shift and range movement, indeed none of the groups did. Last, analytic considerations. The apparent range movement or otherwise we found could have been an artifact of the latitude of the isotherms used in relation to the taxon ranges. Our analysis does not support this suggestion since the isotherms chosen straddle the centre of the geographic area sampled and generally straddle the range centres and limits of the taxa analysed, especially the diatoms and dinoflagellates.

While we present evidence to discount the latter two as potential explanations of the differences in range movements across taxa reported here, we do not otherwise attempt to favour one or more explanation(s) over the others. Disentangling the underlying causes of the different range changes we observed will clearly be a challenge. Yet regardless of the underlying causes, the differences in range changes across taxa are clearly profound and highlight important spatial re-organization of plankton communities over recent decades.

Within the complexity of variable range changes across taxa it was, however, clearly evident that diatoms showed a greater tendency for niche plasticity compared with the dinoflagellates. While the causes for this dichotomy are unknown it is noteworthy that this finding appears to support the change in relative abundance of diatoms to dinoflagellates in recent decades in the North Atlantic^21^ that were linked to increasing windiness with probable knock-on consequences for stratification. In this case niche plasticity has been linked to increased relative abundance, reiterating the major differences across taxa in their responses to long-term environmental change.

The largest range shifts we found are comparable with those of up to 1,000 km reported in the literature^3^,^4^,^13^ and with the movement of copepods of up to 10° north (∼1,110 km) in the area east of 20° W in the North Atlantic from the early to mid-1980s to 1999 (ref. 2). These range changes remain among the most extreme reported across terrestrial and aquatic systems, but it should be noted that these extreme poleward shifts do not reflect the movement of all taxa, many taxa moved far smaller distances despite major environmental changes across several decades. Our evidence for marked differences in the extent of niche plasticity versus conservatism across taxa points to a major spatial reorganization of plankton communities, rather than existing communities simply moving northwards collectively as ocean temperatures warm. This reorganization will presumably have major implications for grazing pressures on phytoplankton thereby impacting higher trophic levels, including fisheries^4^,^19^, biogeochemical cycling^1^,^7^,^12^ and biodiversity^7^,^11^,^30^. Predicting these various impacts remains a complex and important challenge.

## Methods

### Data collection

For this analysis, we used data collected using CPR machines and collated by the Sir Alister Hardy Foundation for Ocean Science (SAHFOS). The CPR survey is the longest-running plankton survey in the world^14^,^20^, commencing in 1931 and with continuous data from 1946.

The CPR survey is described in more detail elsewhere^20^,^31^,^32^ and is summarized here. The CPR machines are towed behind volunteer commercial shipping vessels at ∼6–7 m depth. Plankton are filtered by a slowly scrolling mesh (size 270 μm) and sandwiched by a second mesh before being rolled up in 4% formaldehyde. Although some phytoplankton may escape this mesh size, the proportions of these taxa captured are consistent and comparable between samples, the SAHFOS methods of analysis of these samples have remained consistent since 1958. Data are not collected <10 km from any coast to avoid interference from local conditions. We use data from the most consistently sampled areas of the survey.

The SST data are from the Hadley Centre of the UK Meteorological Office^33^ (http://www.metoffice.gov.uk/hadobs/hadisst/).

### Sample size

The data used were collected in the NE Atlantic and North Sea in the area bounded by 45–64° N, 20° W–8° E, over six decades from 1954 to 2013. The sample size (*n*) was as follows:

*n*=148,265 for *Metridia lucens*, *Metridia longa*, *Pleuromamma robusta*, *Pleuromamma abdominalis*, *Pleuromamma borealis*, *Pleuromamma gracilis*, *Skeletonema costatum*, *Rhizosolenia styliformis*, *Rhizosolenia hebetata semispina*, *Thalassiothrix longissima*, *Thalassionema nitzschioides*, *Eucampia zodiacus*, *Pseudo-nitzschia complex*, *Pseudo-nitzschia seriata complex*, *Proboscia indica*, *Rhizosolenia imbricata*, *Euchaeta acuta*, *Undeuchaeta plumosa*, *Euchaeta hebes*, *Thalassiosira spp.*, *Dinophysis spp.*

*n*=147,194 for *Ceratium fusus*, *Ceratium furca*, *Ceratium lineatum*, *Ceratium tripos*, *Ceratium macroceros*, *Ceratium longipes*, *Ceratium hexacanthum*, *Ceratium minutum*, *Protoperidinium spp.*, *Prorocentrum spp.*

*n*=129,270 for *Calanus finmarchicus*, *Calanus helgolandicus*, *Ditylum brightwellii* (data were not collected before 1958 for these three species).

*n*=75,414 for *Noctiluca scintillans* (data were not collected before 1981 for this species).

### Analysis

The data collated by SAHFOS include latitude, longitude, date, time and abundance. Monthly data were estimated for each 5-year period (1954–1958 January, 1954–1958 February to 2009–2013 December) using ordinary kriging to produce abundance maps for the geographic area 45–64° N, 20° W–8° E. Geostatistical methods such as kriging assume stable spatial structures over the sampling period^34^, as this is not the case with six decades of CPR data we used the shorter periods^35^ of 5-yearly monthly intervals. The ordinary kriging was applied to these data sets to estimate abundances for each 0.5 × 0.5° geographic location and the means of these geographic location estimates for each month were then used to produce 5-yearly maps of the distribution and abundance of each taxon. These 5-yearly data sets were used for all subsequent analysis with the exception of the phenological analysis and to produce all the tables and figures with the exception of Fig. 7 and the abundance maps in Supplementary Figs 4a–42a, the latter presenting decadal averages of each pair of 5-year data sets for all taxa. For the genera *Metridia* and *Pleuromamma*, taxa which exhibit diel vertical migration, abundance figures from only 6 pm–6 am local time were used. The abundance data were log transformed (log(*n*+1)) before analysis. The kriging was applied using the packages ‘sp'^36^, ‘gstat'^37^ and ‘automap'^38^ in the R^39^ statistical language.

Population numbers for each 5-year period were derived from the means of the values estimated by the ordinary kriging at each 0.5° longitude and latitude position for each 5-year period and are graphed in Fig. 5b,d and Supplementary Figs 4b–42b. All these figures include a loess smoother for locally weighted polynomial regression. Descriptive statistics are listed in Table 1d and Supplementary Table 3 for the 5-year periods 1954–1958 to 2009–2013.

Monthly mean SST values were obtained from the HADISST data. These are on a 1 × 1° grid. As with the CPR data, we divided the SST data into 5-year monthly intervals and used ordinary kriging on these data sets to estimate SST values for each 0.5 × 0.5° geographic location. Using these estimates, polynomial regression was used to find the latitude of isotherms at 11, 12 and 13 °C at each 0.5° longitude in each period, these isotherms chosen to straddle the centre of the geographic area and taxon ranges sampled (Supplementary Fig. 3). The mean latitudes of the three isotherms for each 5-year period are presented in Supplementary Table 6 and in Fig. 1b.

Using the estimates of abundance at each geographic location in each period, the percentage of each taxon population north of each isotherm was estimated using polynomial regression at each 0.5° longitude. These normalized percentages are presented in Supplementary Figs 1 and 4d–42d.

**Figure 5:**
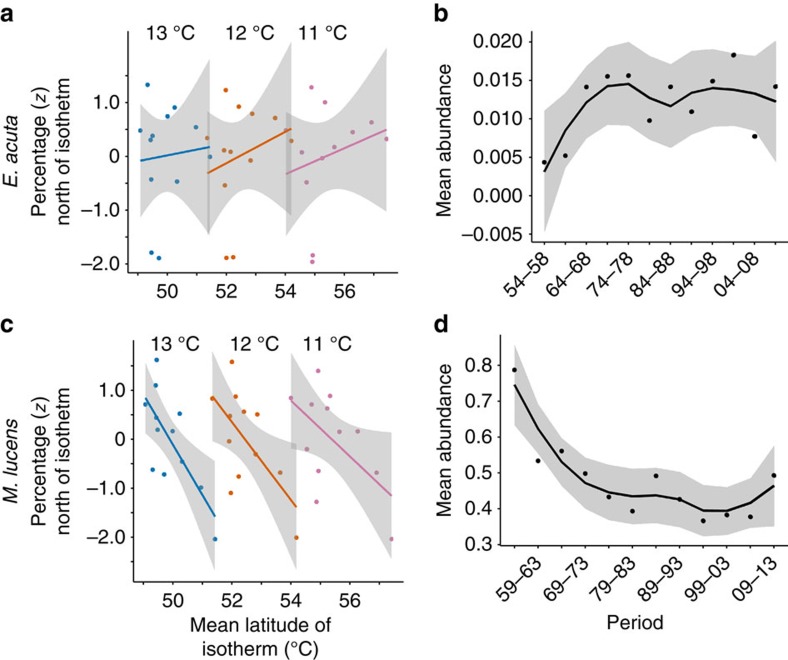
The proportions (normalized) of two individual copepod taxa north of three isotherms and their abundances in the twelve 5-year periods. *Euchaeta acuta* on the top row did not exhibit evidence of niche plasticity in relation to thermal change; *Metridia lucens* on the bottom row did exhibit evidence of niche plasticity. (**a**,**c**) Proportions (normalized) of the populations of the two species north of isotherms at 11, 12 and 13 °C in the twelve 5-year periods from 1954–1958 to 2009–2013. The isotherms move north as the SST rises; *Euchaeta acuta* exhibits no correlation (at *P*<0.05) between the normalized population percentages north of each isotherm and the mean latitude of the isotherms; *Metridia lucens* exhibits a significant negative correlation. A loess smoother was used for locally weighted polynomial regression, the grey area indicating the 95% confidence interval for the line. The *P* values are listed in Supplementary Table 1b. (**b**,**d**) Abundance: the mean of the values derived by kriging at each (longitude, latitude) for each 5-year period.

**Figure 6:**
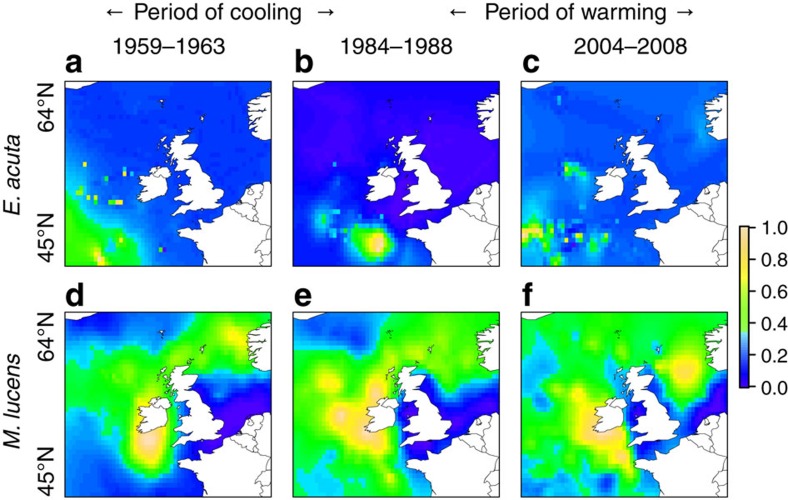
Movement of two individual copepod taxa in cooling and warming periods. (**a–f**) Maps of the totals of the log-transformed (log(*x*+1)) abundance determined by ordinary kriging. The maps were independently scaled 0.0–1.0 to highlight population movements. In the periods of cooling then warming the *E. acuta* median latitude moved south 5 km then north 182 km while the *M. lucens* median latitude moved south 87 km then continued south 38 km.

**Figure 7:**
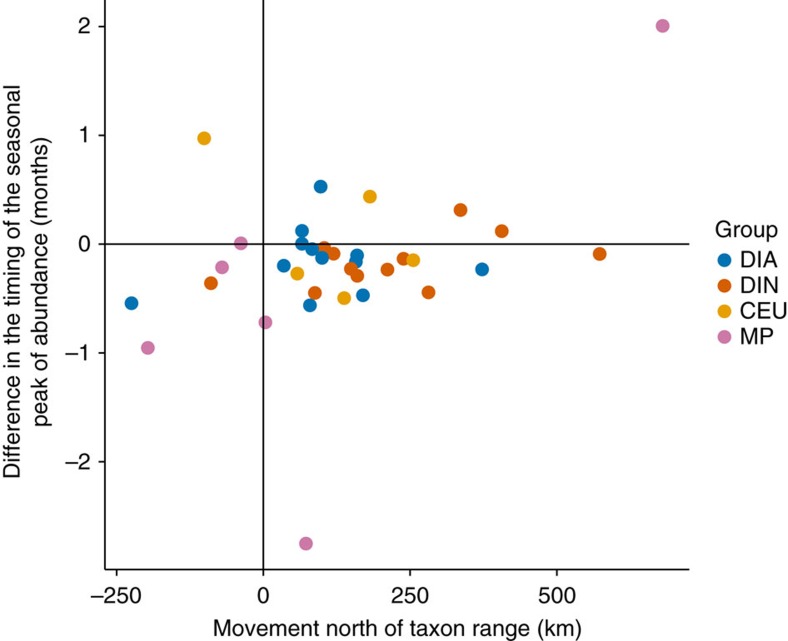
Poleward movement and phenological shift in the warming period. For each taxon, the movement of the range north and the shift in timing of the seasonal peak in abundance in the warming period of 1984–2008 are shown. The poleward movements are as reported in Fig. 4. The phenological shift is the difference in the timing of the seasonal peak of abundance over the same period. No significant correlations (*P*<0.05) were found: DIA *P*=0.5842, DIN *P*=0.1099, CEU *P*=0.2918, MP *P*=0.1594. CEU, *Calanus*, *Euchaeta* and *Undeuchaeta*; DIA, diatoms; DIN, dinoflagellates; MP, *Metridia* and *Pleuromamma*.

To look for effects of isotherm latitude on the percentage of each taxon north of the isotherm we first adjusted for serial autocorrelation using the Chelton method^40^ to re-estimate the number of degrees of freedom. Table 1a and Supplementary Tables 1a,b list the significance of negative correlations between the percentages (*z*-scores) of (a) four combined taxonomic groups and (b) the individual taxa north of isotherms at 11, 12 and 13 °C and the mean latitude of the isotherms for the 5-year periods. Figures 2a,b and 5a,c and Supplementary Figs 4c–42c graph these correlations. For figures which include multiple taxa the means of independently normalized populations were used. A loess smoother was used for locally weighted polynomial regression in all these figures. Table 1c and Supplementary Tables 2a,b highlight correlations between normalized populations of (a) four taxonomic groups and (b) the individual taxa and mean sea surface temperature for the periods 1984–1988 to 2004–2008 (a period of consistent warming) and 1954–1958 to 2009–2013.

Using established methodology^14^, we calculated seasonal timing of peak abundance as follows:


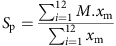


where *S*_p_ is the seasonal peak, *M* is the number of the month (1–12) and *x*_m_ is the mean abundance in the month. Nine of the thirty-five taxa exhibited population peaks in both spring and autumn, in these cases we used the most populous peaks.

### Code availability

All code was written in the in the R^39^ statistical language, which is open source and freely available. Enquiries about the code used here can be directed to the corresponding author, W.J.C.

### Data availability

The data that support the findings of this study are publicly available.

1. The plankton population data are available from the Sir Alister Hardy Foundation for Ocean Science (SAHFOS): https://www.sahfos.ac.uk/.
2. The temperature data are available from the Hadley Centre Sea Ice and Sea Surface Temperature data set (HadISST): http://www.metoffice.gov.uk/hadobs/hadisst/.

## Additional information

**How to cite this article:** Chivers, W. J. *et al*. Mismatch between marine plankton range movements and the velocity of climate change. *Nat. Commun.* **8,** 14434 doi: 10.1038/ncomms14434 (2017).

**Publisher's note**: Springer Nature remains neutral with regard to jurisdictional claims in published maps and institutional affiliations.

## Supplementary Material

Supplementary Information Supplementary Figures 1-42, Supplementary Tables 1-6.

## Figures and Tables

**Table 1:** Details of each taxon and isotherms at 11, 12 and 13 °C.

**Taxon**	**a**	**b**		**c**	**d**	
	**Negative correlation (*****P*****<0.05): proportion north and isotherm latitudes**	**Movement north (km) of range median latitude**		**Correlation: taxon abundance with SST**	**Taxon abundance**	
		**Cooling period**	**Warming period**		**1954–1958 to 2009–2013**	
	**1954–1958 to 2009–2013**	**1959–1963 to 1984–1988**	**1984–1988 to 2004–2008**	**1954–1958 to 2009–2013**	**Mean**	**s.d.**
*Diatoms*						
Ditylum brightwellii*		−420.56	372.59	+	0.1307	0.0715
*Eucampia zodiacus*	***	56.20	−224.64		0.0550	0.0227
*Pseudo-nitzschia complex*	***	−56.41	159.63		0.9588	0.3779
*Pseudo-nitzschia seriata complex*	***	−136.93	83.11		0.8218	0.2854
*Proboscia indica*	**	−66.26	169.63		0.3438	0.1881
*Rhizosolenia hebetata semispina*	***	−61.16	97.59		0.5862	0.1417
*Rhizosolenia imbricata*	***	−21.88	65.82		0.6377	0.2714
*Rhizosolenia styliformis*	***	−137.41	65.72	−	0.7889	0.2702
*Skeletonema costatum*		11.90	157.61		0.1933	0.0988
*Thalassiosira spp.*	***	−135.22	34.81	+	1.9102	0.3656
*Thalassiothrix longissima*	***	−147.47	79.34		0.5123	0.3841
*Thalassionema nitzschioides*	***	−98.02	99.99		1.3269	0.4794
						
*Dinoflagellates*						
*Ceratium furca*	**	−101.53	238.79	−	1.6862	0.5181
*Ceratium fusus*	***	−104.42	103.66	−	2.5022	0.6574
*Ceratium lineatum*	*	−165.74	159.97	−	0.6399	0.2109
*Ceratium tripos*	**	−76.19	149.08	−	1.0716	0.3533
*Ceratium macroceros*		−363.97	406.08		0.6990	0.3994
*Ceratium longipes*		−1.94	335.85		0.3721	0.2289
*Ceratium minutum*	***	116.35	119.54		0.0587	0.0324
*Ceratium hexacanthum*		−74.07	572.92		0.1088	0.0657
*Dinophysis spp.*	***	83.70	−89.27	−	0.2946	0.0898
*Noctiluca scintillans*†		NA	211.33	+	0.2157	0.1014
*Prorocentrum spp.*		−353.82	281.29	−	0.1333	0.0654
*Protoperidinium spp.*	***	−59.84	87.79	−	0.6387	0.2076
						
*Copepods*						
Calanus finmarchicus*		46.11	137.62	−	0.5730	0.1721
Calanus helgolandicus*		12.36	255.30	+	0.5865	0.1266
*Euchaeta acuta*		−4.52	181.51		0.0121	0.0044
*Euchaeta hebes*		296.32	−100.86	+	0.0311	0.0208
*Undeuchaeta plumosa*		37.67	57.78		0.0109	0.0046
*Metridia longa*		−492.04	680.17		0.0045	0.0046
*Metridia lucens*	***	−87.49	−38.32	−	0.4789	0.1167
*Pleuromamma abdominalis*	***	105.99	−70.40		0.0132	0.0094
*Pleuromamma borealis*		224.49	72.57	+	0.0427	0.0224
*Pleuromamma gracilis*	***	248.61	−196.79	+	0.0468	0.0203
*Pleuromamma robusta*	***	−71.12	3.45	−	0.1041	0.0512
						
*Isotherms*						
11 °C		−136.53	377.40			
12 °C		−87.69	315.24			
13 °C		−38.85	260.85			

a, significant negative correlations between the proportions of each taxon north of isotherms at 11, 12 and 13 °C and the mean latitude of the isotherms in the twelve 5-year periods 1954–2013 (unless noted) during which there were periods of cooling and warming. A significant negative correlation indicates niche plasticity in relation to thermal change. See Supplementary Table 1b for more detail. b, northerly movement of range median latitude in the cooling period of 1959–1963 to 1984–1988 and the warming period of 1984–1988 to 2004–2008. See Supplementary Table 4 for more detail. c, positive or negative correlations between population and mean SST in the warming period and/or the twelve 5-year periods 1954–2013 (unless noted). See Supplementary Table 2b for more detail. d, population mean and s.d. in the twelve 5-year periods 1954–2013 (unless noted). See Supplementary Table 3 for more detail.

^*^Data from 1959–1963 to 2009–2013.

^†^Data from 1984–1988 to 2009–2013.
