# Supplementary material for: Mismatch between marine plankton range movements and the velocity of climate change
Source: Nat Commun. 2017 Feb 10;8:14434. doi: 10.1038/ncomms14434 (PMC5309926; doi:10.1038/ncomms14434)
Supplement: Supplementary Information — Supplementary Figures 1-42, Supplementary Tables 1-6. [file ncomms14434-s1.pdf]

1 Supplementary figures

1.1 Percentage of the combined taxonomic groups north of three isotherms over 12 five-year periods.

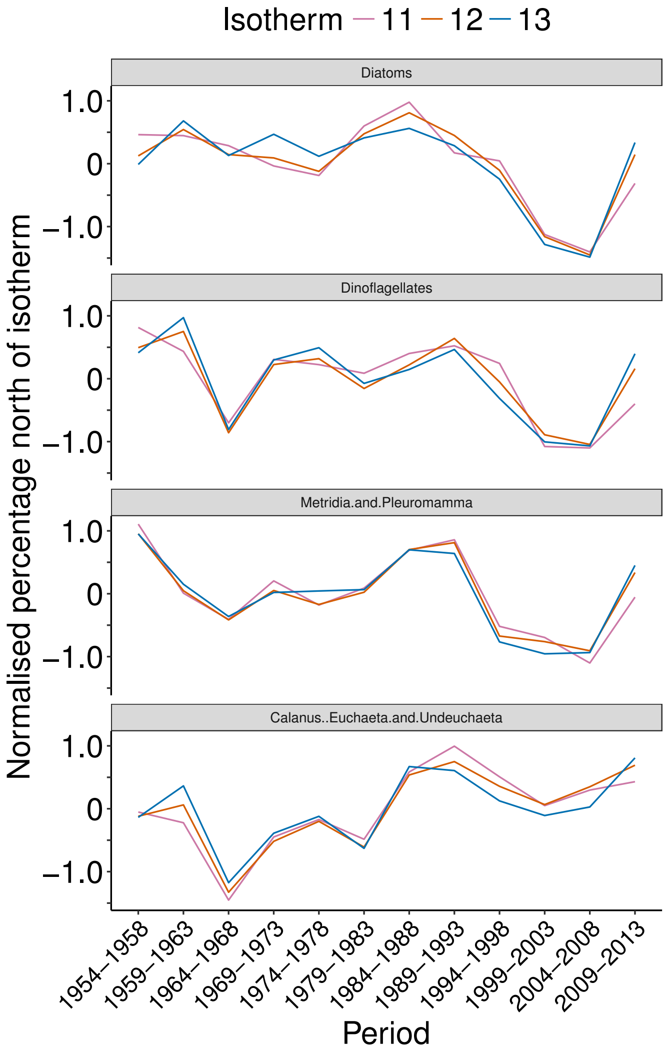

Figure 1: Percentage north of the three isotherms of the combined taxonomic groups over time. For each taxon polynomial regression at each longitude was used to estimate the percentage of the taxon north of the the estimated latitudes of the isotherms. The graphs use the means of the independently normalised taxa for each taxonomic group. Negative correlation between the percentage north of an isotherm and the isotherm latitude indicates niche plasticity in relation to thermal change, so a falling percentage in these graphs in the period 1984–2008 (during which there was constant warming) suggests niche plasticity.

1.2 Northerly movement of taxa and change in the proportion north of the isotherms in the warming period.

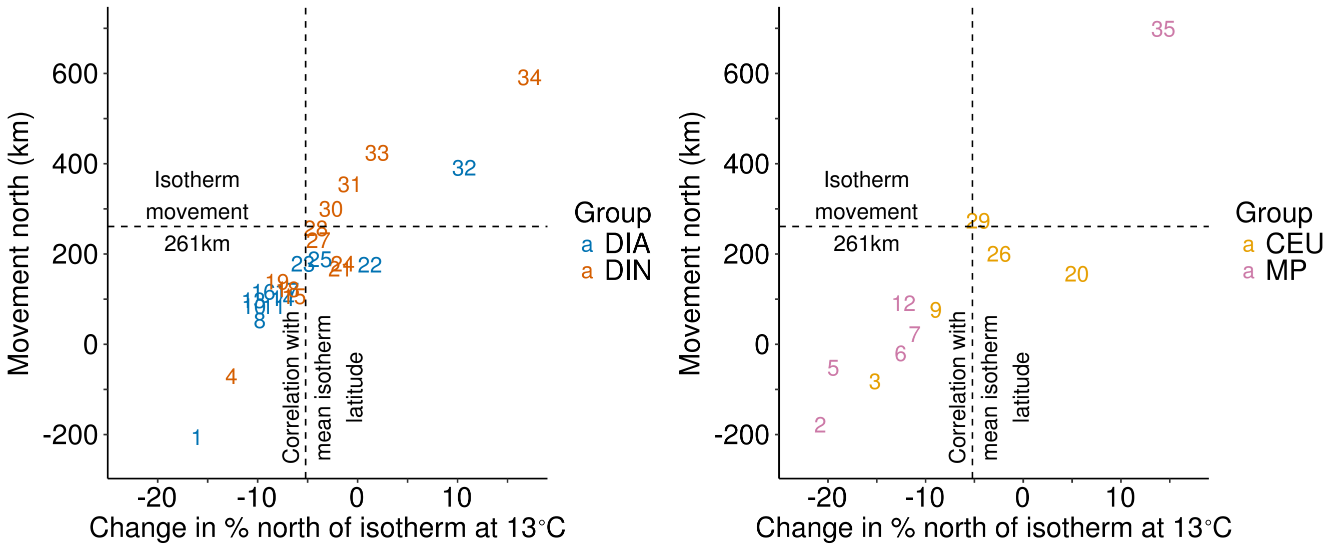

a Diatoms and Dinoflagellates **b** Copepods

Figure 2: Northerly movement of range median latitude and change in the proportion north of the isotherm at 13°C for all diatom, dinoflagellate and copepod taxa in the period 1984–1988 to 2004–2008, a period during which there was constant warming. The horizontal dashed line indicates the northerly movement of the isotherm in this period and the vertical dashed line approximately indicates the taxa which exhibit correlation between the proportion north of the isotherm and the mean latitude of the isotherm (those exhibiting correlation are generally to the left of the line, please see Supplementary Table 1b). A significant correlation indicates niche plasticity in relation to thermal change. **DIA:** Diatoms; **DIN:** Dinoflagellates; **CEU:** *Calanus*, *Euchaeta* and *Undeuchaeta*; **MP:** *Metridia* and *Pleuromamma*.

1: *Eucampia zodiacus*, 2: *Pleuromamma gracilis*, 3: *Euchaeta hebes*, 4: *Dinophysis spp.*, 5: *Pleuromamma abdominalis*, 6: *Metridia lucens*, 7: *Pleuromamma robusta*, 8: *Thalassiosira spp.*, 9: *Undeuchaeta plumosa*, 10: *Rhizosolenia styliformis*, 11: *Rhizosolenia imbricata*, 12: *Pleuromamma borealis*, 13: *Thalassiothrix longissima*, 14: *Pseudo-nitzschia seriata complex*, 15: *Protoperidinium spp.*, 16: *Rhizosolenia hebetata semispina*, 17: *Thalassionema nitzschioides*, 18: *Ceratium fusus*, 19: *Ceratium minutum*, 20: *Calanus finmarchicus*, 21: *Ceratium tripos*, 22: *Skeletonema costatum*, 23: *Pseudo-nitzschia complex*, 24: *Ceratium lineatum*, 25: *Proboscia indica*, 26: *Euchaeta acuta*, 27: *Noctiluca scintillans*, 28: *Ceratium furca*, 29: *Calanus helgolandicus*, 30: *Prorocentrum spp.*, 31: *Ceratium longipes*, 32: *Ditylum brightwellii*, 33: *Ceratium macroceros*, 34: *Ceratium hexacanthum*, 35: *Metridia longa*.

1.3 Temperature gradients and isotherms

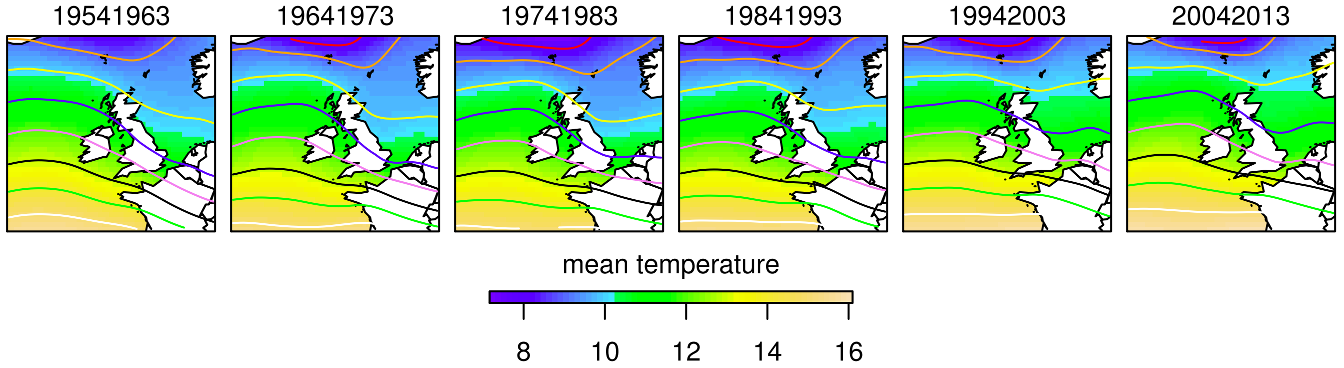

Figure 3: Positions of isotherms in the area 45–64°N, 20°W–8°E over six decades. Key: 8°C red; 9°C orange; 10°C yellow; 11°C blue; 12°C violet; 13°C black; 14°C green; 15°C white.

2 All Diatoms—abundance maps and associated graphs

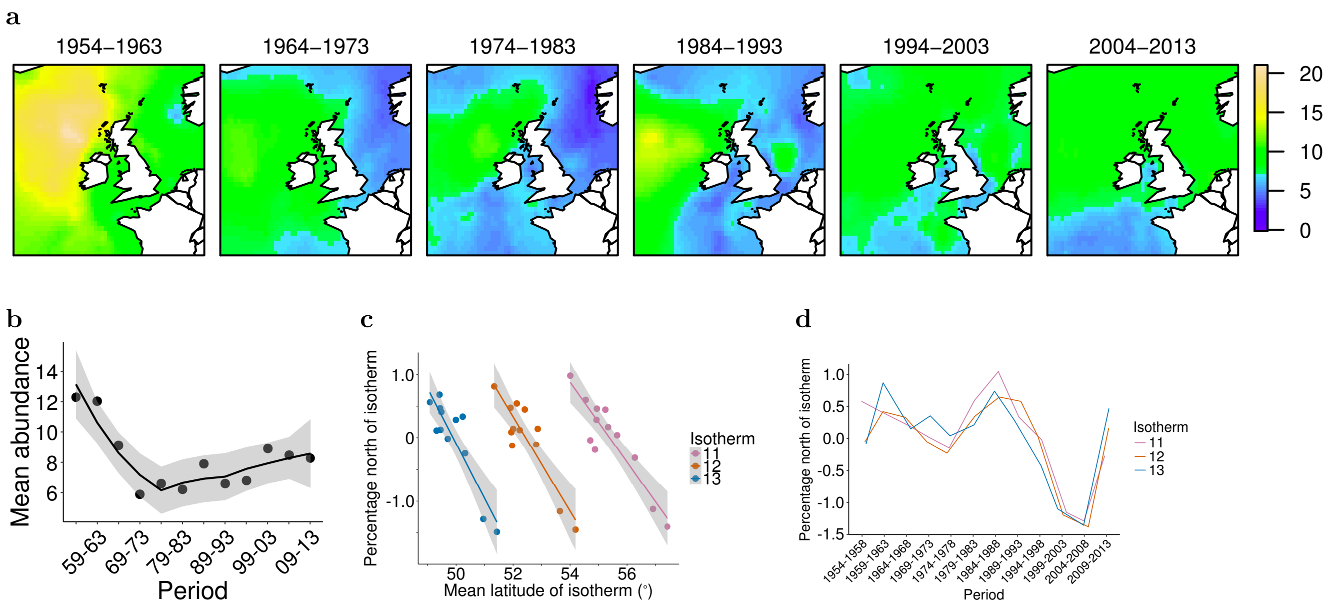

Figure 4: All 12 taxa of diatoms.

a, Sum of the log-transformed ($log(x + 1)$) cell counts per (longitude, latitude) determined by ordinary kriging for each taxon in this group. The ten-year periods are the means of the five-year pairs. The geographic area is 45–64°N, 20°W–8°E, the scale illustrates temporal and spatial abundance.

b, Abundance: the sum of the means for each taxon of the values derived by kriging at each (longitude, latitude) for each five-year period (and used to create the maps above). A loess smoother was used for locally weighted polynomial regression, the grey area indicating the 95% confidence interval for the line.

c, Proportions (normalised) of the taxon population north of isotherms at 11, 12 and 13°C within the geographic area 45–64°N, 20°W–8°E in the 12 five-year periods from 1954–1958 to 2009–2013. The isotherms move north as the SST rises; these plots illustrate correlations between the normalised population percentages north of each isotherm and the mean latitude of the isotherms. A loess smoother was used for locally weighted polynomial regression, the grey area indicating the 95% confidence interval for the line. The p-values are listed in Supplementary Table 1a.

d, Normalised percentage of the combined taxonomic group north of three isotherms over time. Negative correlation between the percentage north of an isotherm and the isotherm latitude indicates niche plasticity in relation to thermal change, so a falling percentage in this graph in the period 1984–2008 (during which there was constant warming) suggests niche plasticity.

2.1 *Ditylum brightwellii*

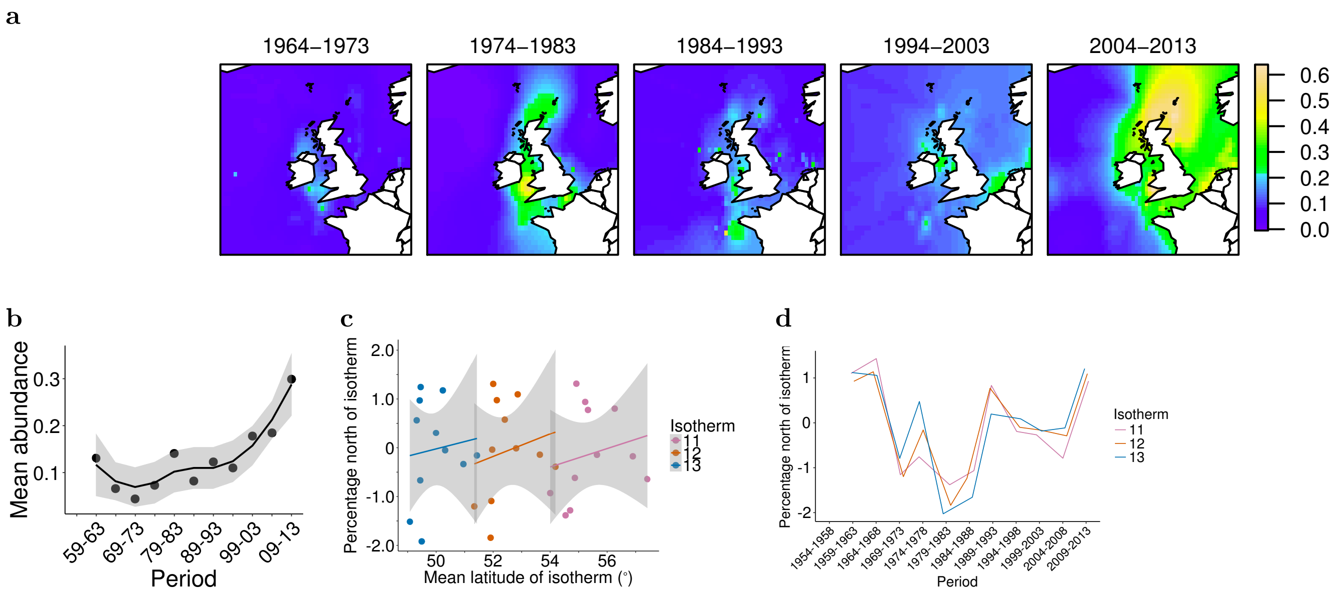

Figure 5: *Ditylum brightwellii.* Note that there were no data collected for this species before 1958.

a, Mean cell count per sample ($log(x + 1)$) of combined pairs of five-year periods, 45–64°N, 20°W–8°E. The scale illustrates temporal and spatial abundance.

b, Abundance: the mean of the values derived by kriging at each (longitude, latitude) for each five-year period (and used to create the maps above). A loess smoother was used for locally weighted polynomial regression, the grey area indicating the 95% confidence interval for the line. Descriptive statistics: max 0.299, min 0.0449, mean 0.1307, SD 0.0715.

c, Proportions (normalised) of the taxon population north of isotherms at 11, 12 and 13°C within the geographic area 45–64°N, 20°W–8°E in the 12 five-year periods from 1954–1958 to 2009–2013. The isotherms move north as the SST rises; these plots illustrate correlations between the normalised population percentages north of each isotherm and the mean latitude of the isotherms. A loess smoother was used for locally weighted polynomial regression, the grey area indicating the 95% confidence interval for the line. The *p*-values are listed in Supplementary Table 1b.

d, Normalised percentage of the taxonomic group north of three isotherms over time. Negative correlation between the percentage north of an isotherm and the isotherm latitude indicates niche plasticity in relation to thermal change, so a falling percentage in this graph in the period 1984–2008 (during which there was constant warming) suggests niche plasticity.

2.2 *Eucampia zodiacus*

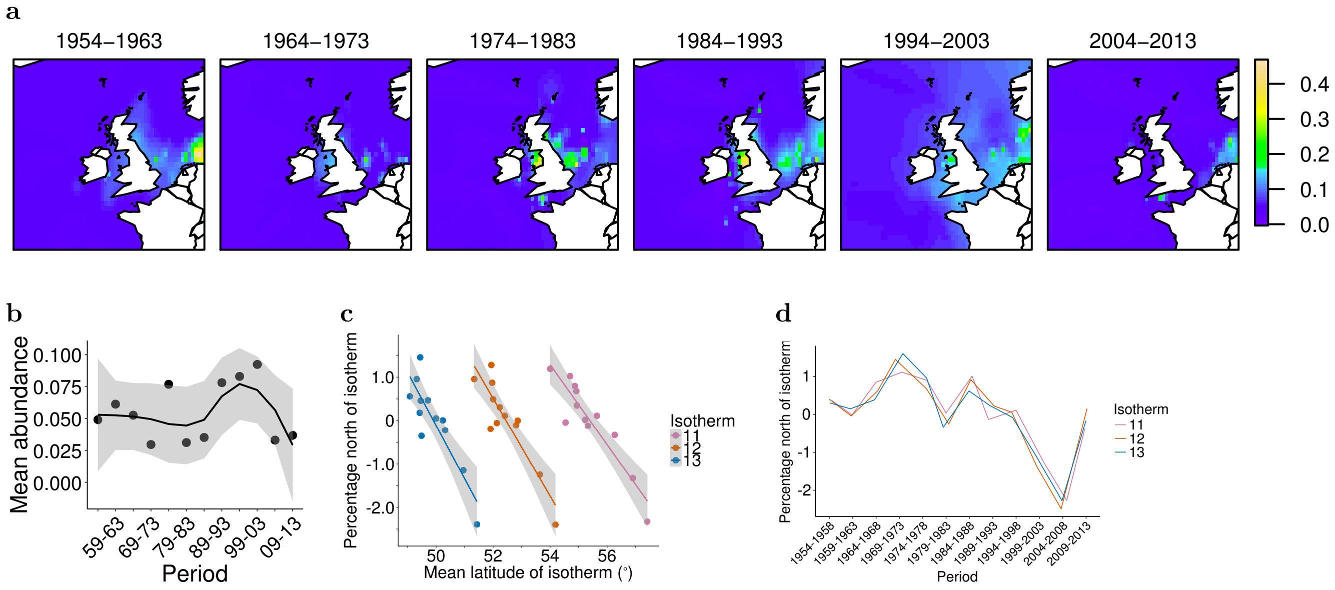

Figure 6: *Eucampia zodiacus*.

a, Mean cell count per sample ($log(x + 1)$) of combined pairs of five-year periods, 45–64°N, 20°W–8°E. The scale illustrates temporal and spatial abundance.

b, Abundance: the mean of the values derived by kriging at each (longitude, latitude) for each five-year period (and used to create the maps above). A loess smoother was used for locally weighted polynomial regression, the grey area indicating the 95% confidence interval for the line. Descriptive statistics: max 0.0923, min 0.0298, mean 0.055, SD 0.0227.

c, Proportions (normalised) of the taxon population north of isotherms at 11, 12 and 13°C within the geographic area 45–64°N, 20°W–8°E in the 12 five-year periods from 1954–1958 to 2009–2013. The isotherms move north as the SST rises; these plots illustrate correlations between the normalised population percentages north of each isotherm and the mean latitude of the isotherms. A loess smoother was used for locally weighted polynomial regression, the grey area indicating the 95% confidence interval for the line. The p-values are listed in Supplementary Table 1b.

d, Normalised percentage of the taxonomic group north of three isotherms over time. Negative correlation between the percentage north of an isotherm and the isotherm latitude indicates niche plasticity in relation to thermal change, so a falling percentage in this graph in the period 1984–2008 (during which there was constant warming) suggests niche plasticity.

2.3 *Pseudo-nitzschia complex*

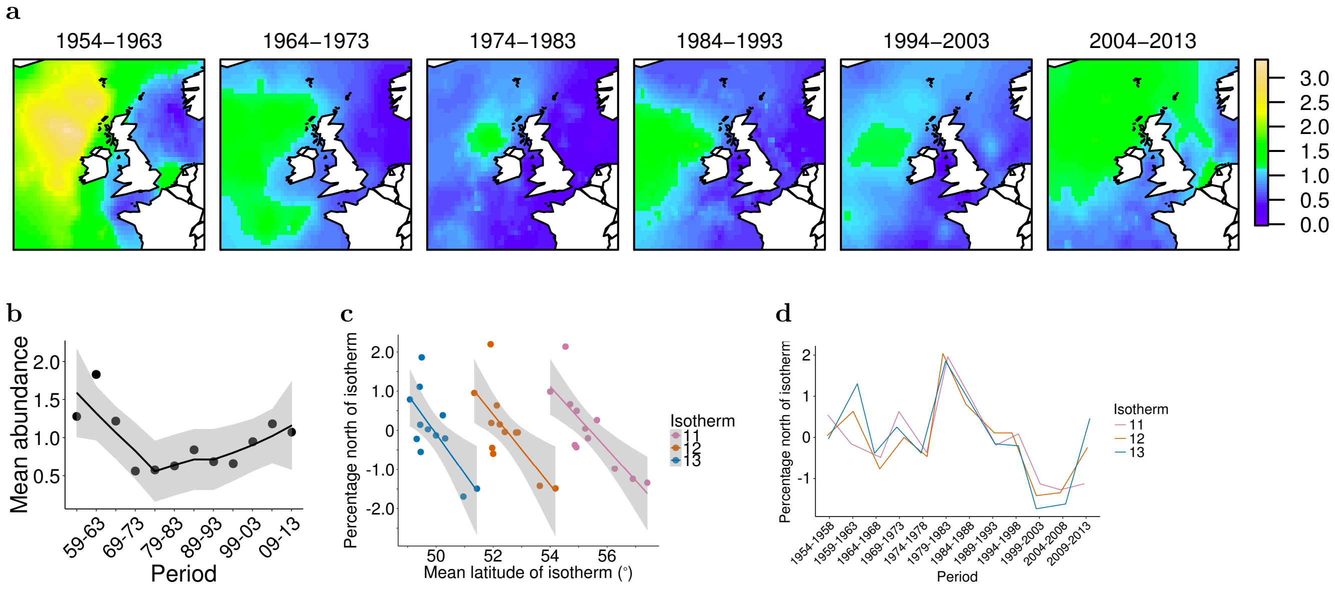

Figure 7: ***Pseudo-nitzschia complex*.**

a, Mean cell count per sample ($log(x + 1)$) of combined pairs of five-year periods, 45–64°N, 20°W–8°E. The scale illustrates temporal and spatial abundance.

b, Abundance: the mean of the values derived by kriging at each (longitude, latitude) for each five-year period (and used to create the maps above). A loess smoother was used for locally weighted polynomial regression, the grey area indicating the 95% confidence interval for the line. Descriptive statistics: max 1.8297, min 0.5653, mean 0.9588, SD 0.3779.

c, Proportions (normalised) of the taxon population north of isotherms at 11, 12 and 13°C within the geographic area 45–64°N, 20°W–8°E in the 12 five-year periods from 1954–1958 to 2009–2013. The isotherms move north as the SST rises; these plots illustrate correlations between the normalised population percentages north of each isotherm and the mean latitude of the isotherms. A loess smoother was used for locally weighted polynomial regression, the grey area indicating the 95% confidence interval for the line. The *p*-values are listed in Supplementary Table 1b.

d, Normalised percentage of the taxonomic group north of three isotherms over time. Negative correlation between the percentage north of an isotherm and the isotherm latitude indicates niche plasticity in relation to thermal change, so a falling percentage in this graph in the period 1984–2008 (during which there was constant warming) suggests niche plasticity.

2.4 *Pseudo-nitzschia seriata complex*

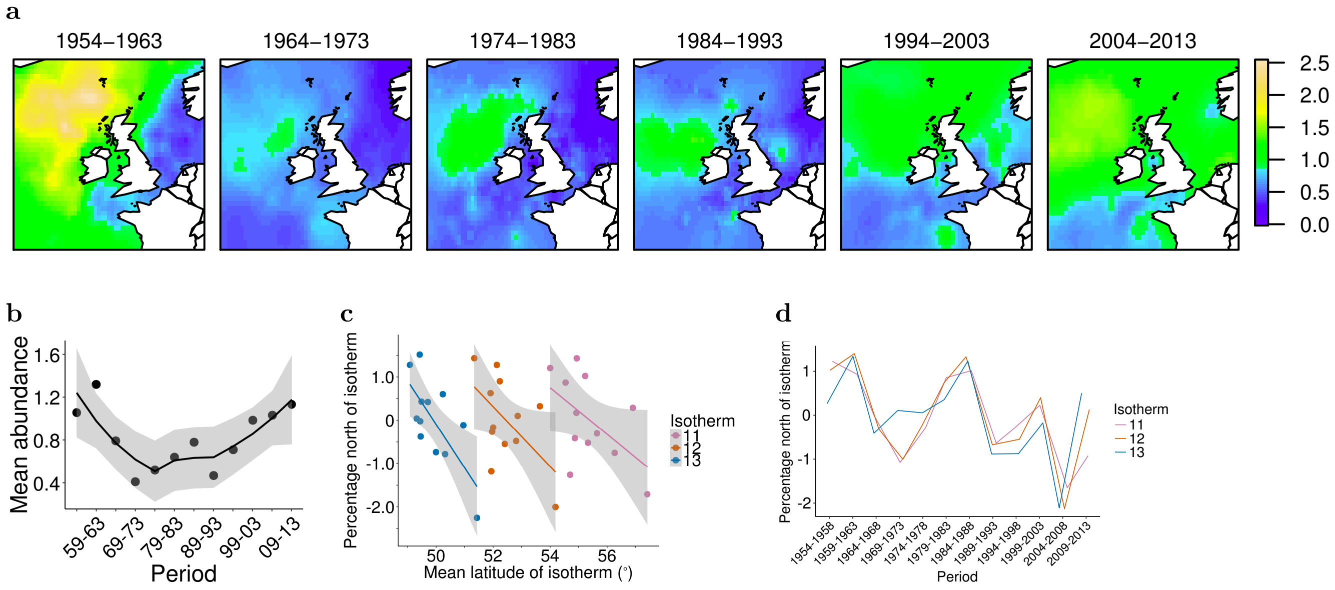

Figure 8: ***Pseudo-nitzschia seriata complex*.**

a, Mean cell count per sample ($log(x + 1)$) of combined pairs of five-year periods, 45–64°N, 20°W–8°E. The scale illustrates temporal and spatial abundance.

b, Abundance: the mean of the values derived by kriging at each (longitude, latitude) for each five-year period (and used to create the maps above). A loess smoother was used for locally weighted polynomial regression, the grey area indicating the 95% confidence interval for the line. Descriptive statistics: max 1.3192, min 0.4137, mean 0.8218, SD 0.2854.

c, Proportions (normalised) of the taxon population north of isotherms at 11, 12 and 13°C within the geographic area 45–64°N, 20°W–8°E in the 12 five-year periods from 1954–1958 to 2009–2013. The isotherms move north as the SST rises; these plots illustrate correlations between the normalised population percentages north of each isotherm and the mean latitude of the isotherms. A loess smoother was used for locally weighted polynomial regression, the grey area indicating the 95% confidence interval for the line. The p-values are listed in Supplementary Table 1b.

d, Normalised percentage of the taxonomic group north of three isotherms over time. Negative correlation between the percentage north of an isotherm and the isotherm latitude indicates niche plasticity in relation to thermal change, so a falling percentage in this graph in the period 1984–2008 (during which there was constant warming) suggests niche plasticity.

2.5 *Proboscia indica*

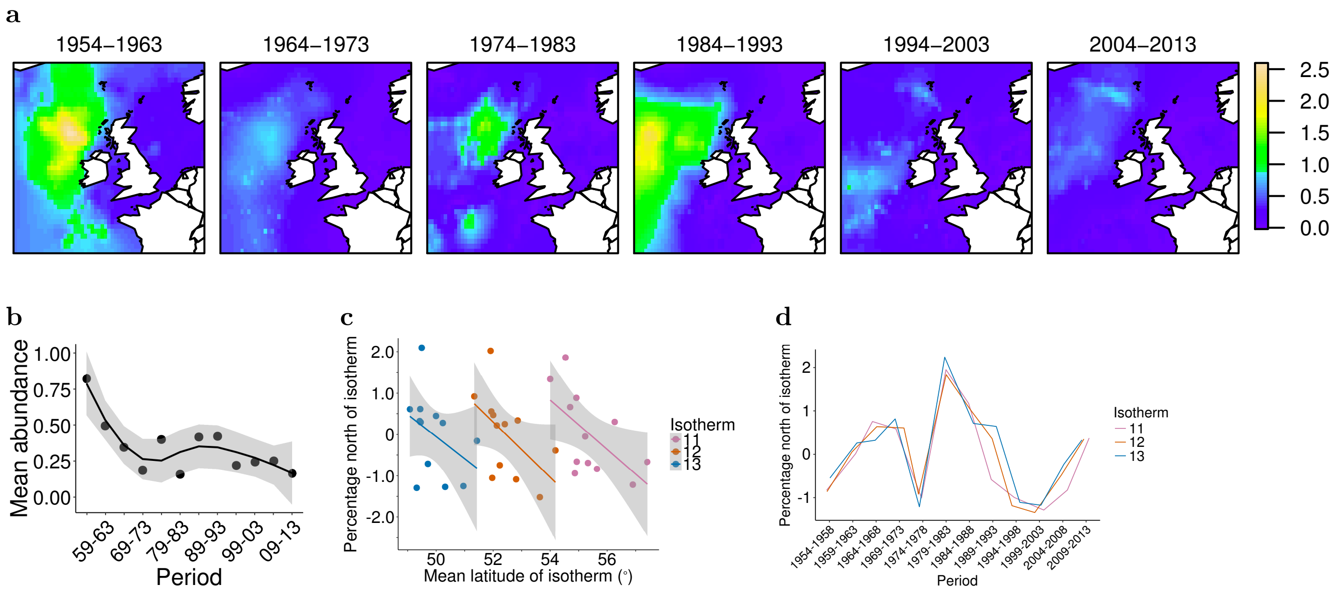

Figure 9: *Proboscia indica*.

a, Mean cell count per sample ($log(x + 1)$) of combined pairs of five-year periods, 45–64°N, 20°W–8°E. The scale illustrates temporal and spatial abundance.

b, Abundance: the mean of the values derived by kriging at each (longitude, latitude) for each five-year period (and used to create the maps above). A loess smoother was used for locally weighted polynomial regression, the grey area indicating the 95% confidence interval for the line. Descriptive statistics: max 0.821, min 0.1595, mean 0.3438, SD 0.1881.

c, Proportions (normalised) of the taxon population north of isotherms at 11, 12 and 13°C within the geographic area 45–64°N, 20°W–8°E in the 12 five-year periods from 1954–1958 to 2009–2013. The isotherms move north as the SST rises; these plots illustrate correlations between the normalised population percentages north of each isotherm and the mean latitude of the isotherms. A loess smoother was used for locally weighted polynomial regression, the grey area indicating the 95% confidence interval for the line. The p-values are listed in Supplementary Table 1b.

d, Normalised percentage of the taxonomic group north of three isotherms over time. Negative correlation between the percentage north of an isotherm and the isotherm latitude indicates niche plasticity in relation to thermal change, so a falling percentage in this graph in the period 1984–2008 (during which there was constant warming) suggests niche plasticity.

2.6 *Rhizosolenia hebetata semispina*

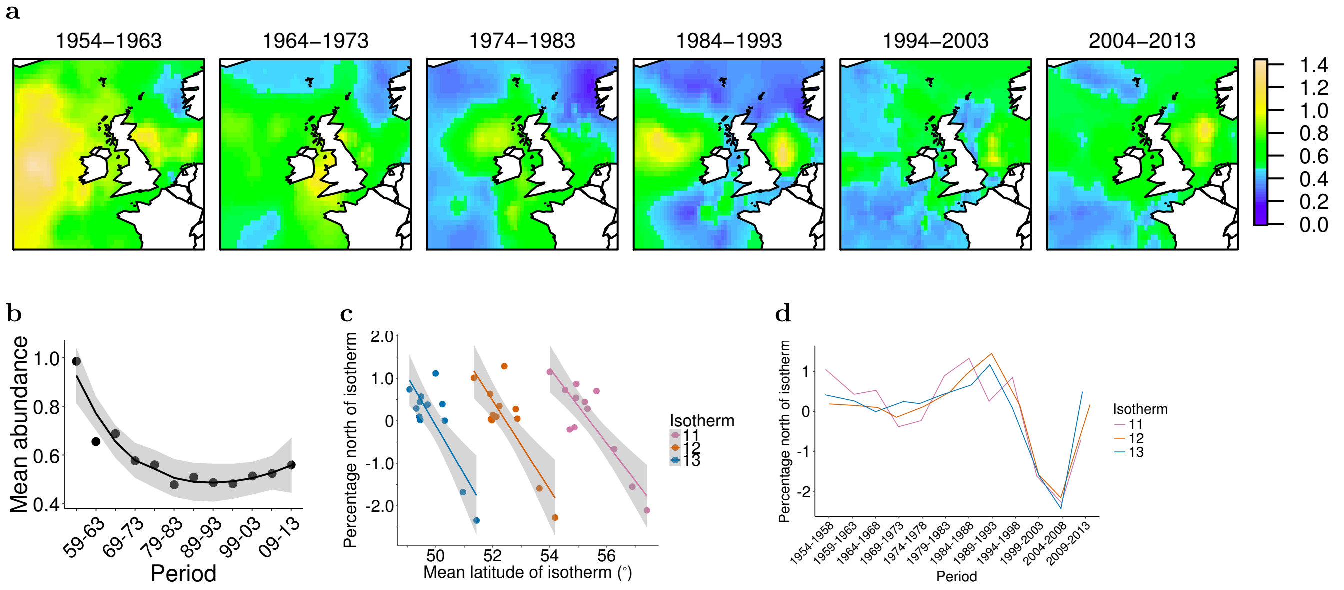

Figure 10: ***Rhizosolenia hebetata semispina*.**

a, Mean cell count per sample ($log(x + 1)$) of combined pairs of five-year periods, 45–64°N, 20°W–8°E. The scale illustrates temporal and spatial abundance.

b, Abundance: the mean of the values derived by kriging at each (longitude, latitude) for each five-year period (and used to create the maps above). A loess smoother was used for locally weighted polynomial regression, the grey area indicating the 95% confidence interval for the line. Descriptive statistics: max 0.9843, min 0.4801, mean 0.5862, SD 0.1417.

c, Proportions (normalised) of the taxon population north of isotherms at 11, 12 and 13°C within the geographic area 45–64°N, 20°W–8°E in the 12 five-year periods from 1954–1958 to 2009–2013. The isotherms move north as the SST rises; these plots illustrate correlations between the normalised population percentages north of each isotherm and the mean latitude of the isotherms. A loess smoother was used for locally weighted polynomial regression, the grey area indicating the 95% confidence interval for the line. The p-values are listed in Supplementary Table 1b.

d, Normalised percentage of the taxonomic group north of three isotherms over time. Negative correlation between the percentage north of an isotherm and the isotherm latitude indicates niche plasticity in relation to thermal change, so a falling percentage in this graph in the period 1984–2008 (during which there was constant warming) suggests niche plasticity.

2.7 *Rhizosolenia imbricata*

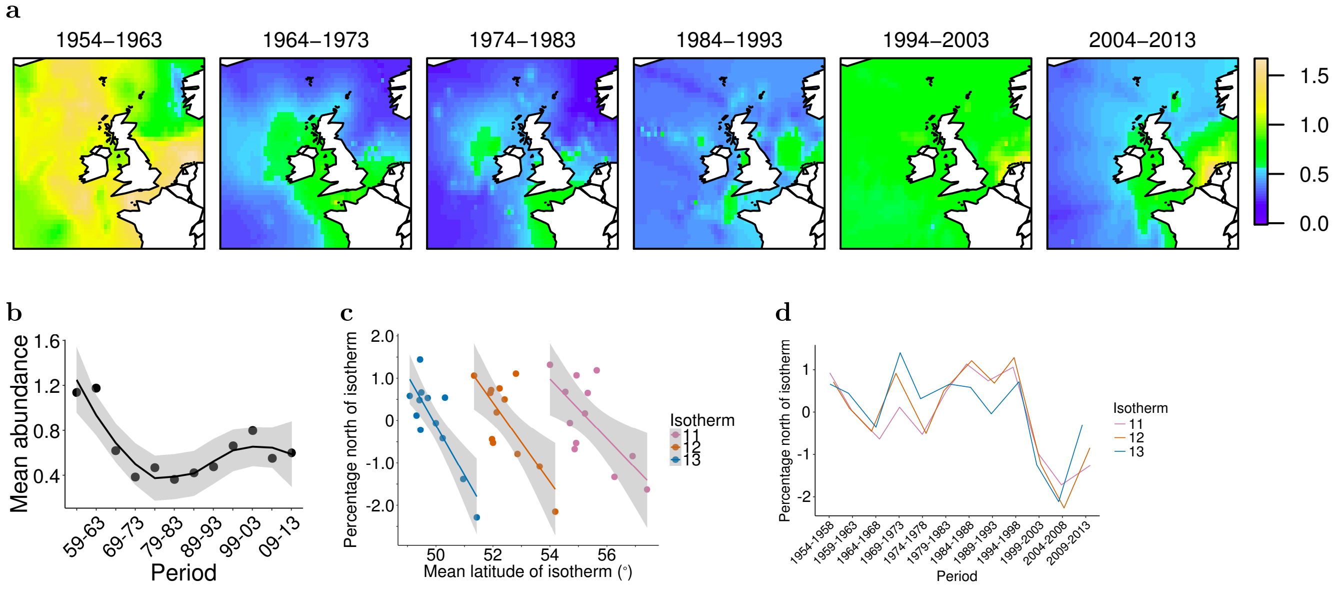

Figure 11: *Rhizosolenia imbricata*.

a, Mean cell count per sample ($log(x+1)$) of combined pairs of five-year periods, 45–64°N, 20°W–8°E. The scale illustrates temporal and spatial abundance.

b, Abundance: the mean of the values derived by kriging at each (longitude, latitude) for each five-year period (and used to create the maps above). A loess smoother was used for locally weighted polynomial regression, the grey area indicating the 95% confidence interval for the line. Descriptive statistics: max 1.1728, min 0.364, mean 0.6377, SD 0.2714.

c, Proportions (normalised) of the taxon population north of isotherms at 11, 12 and 13°C within the geographic area 45–64°N, 20°W–8°E in the 12 five-year periods from 1954–1958 to 2009–2013. The isotherms move north as the SST rises; these plots illustrate correlations between the normalised population percentages north of each isotherm and the mean latitude of the isotherms. A loess smoother was used for locally weighted polynomial regression, the grey area indicating the 95% confidence interval for the line. The *p*-values are listed in Supplementary Table 1b.

d, Normalised percentage of the taxonomic group north of three isotherms over time. Negative correlation between the percentage north of an isotherm and the isotherm latitude indicates niche plasticity in relation to thermal change, so a falling percentage in this graph in the period 1984–2008 (during which there was constant warming) suggests niche plasticity.

2.8 *Rhizosolenia styliformis*

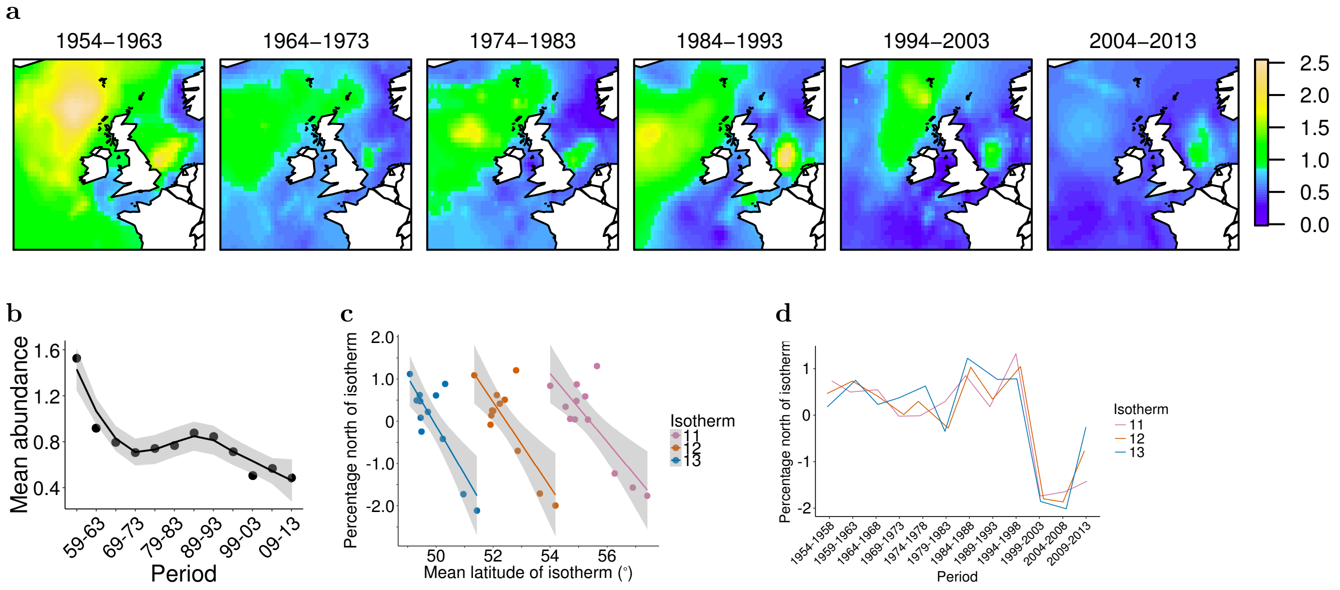

Figure 12: ***Rhizosolenia styliformis*.**

a, Mean cell count per sample ($log(x + 1)$) of combined pairs of five-year periods, 45–64°N, 20°W–8°E. The scale illustrates temporal and spatial abundance.

b, Abundance: the mean of the values derived by kriging at each (longitude, latitude) for each five-year period (and used to create the maps above). A loess smoother was used for locally weighted polynomial regression, the grey area indicating the 95% confidence interval for the line. Descriptive statistics: max 1.5255, min 0.4863, mean 0.7889, SD 0.2702.

c, Proportions (normalised) of the taxon population north of isotherms at 11, 12 and 13°C within the geographic area 45–64°N, 20°W–8°E in the 12 five-year periods from 1954–1958 to 2009–2013. The isotherms move north as the SST rises; these plots illustrate correlations between the normalised population percentages north of each isotherm and the mean latitude of the isotherms. A loess smoother was used for locally weighted polynomial regression, the grey area indicating the 95% confidence interval for the line. The p-values are listed in Supplementary Table 1b.

d, Normalised percentage of the taxonomic group north of three isotherms over time. Negative correlation between the percentage north of an isotherm and the isotherm latitude indicates niche plasticity in relation to thermal change, so a falling percentage in this graph in the period 1984–2008 (during which there was constant warming) suggests niche plasticity.

2.9 *Skeletonema costatum*

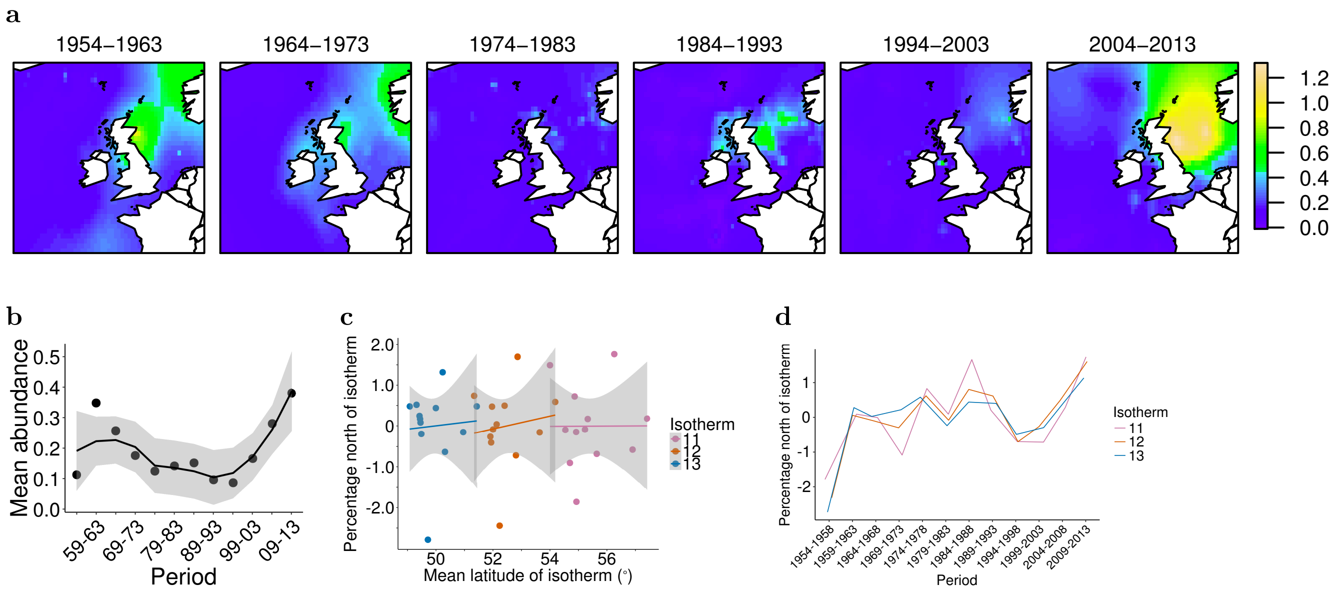

Figure 13: ***Skeletonema costatum*****.**

a, Mean cell count per sample ($log(x + 1)$) of combined pairs of five-year periods, 45–64°N, 20°W–8°E. The scale illustrates temporal and spatial abundance.

b, Abundance: the mean of the values derived by kriging at each (longitude, latitude) for each five-year period (and used to create the maps above). A loess smoother was used for locally weighted polynomial regression, the grey area indicating the 95% confidence interval for the line. Descriptive statistics: max 0.3798, min 0.0867, mean 0.1933, SD 0.0988.

c, Proportions (normalised) of the taxon population north of isotherms at 11, 12 and 13°C within the geographic area 45–64°N, 20°W–8°E in the 12 five-year periods from 1954–1958 to 2009–2013. The isotherms move north as the SST rises; these plots illustrate correlations between the normalised population percentages north of each isotherm and the mean latitude of the isotherms. A loess smoother was used for locally weighted polynomial regression, the grey area indicating the 95% confidence interval for the line. The p-values are listed in Supplementary Table 1b.

d, Normalised percentage of the taxonomic group north of three isotherms over time. Negative correlation between the percentage north of an isotherm and the isotherm latitude indicates niche plasticity in relation to thermal change, so a falling percentage in this graph in the period 1984–2008 (during which there was constant warming) suggests niche plasticity.

2.10 *Thalassiosira spp.*

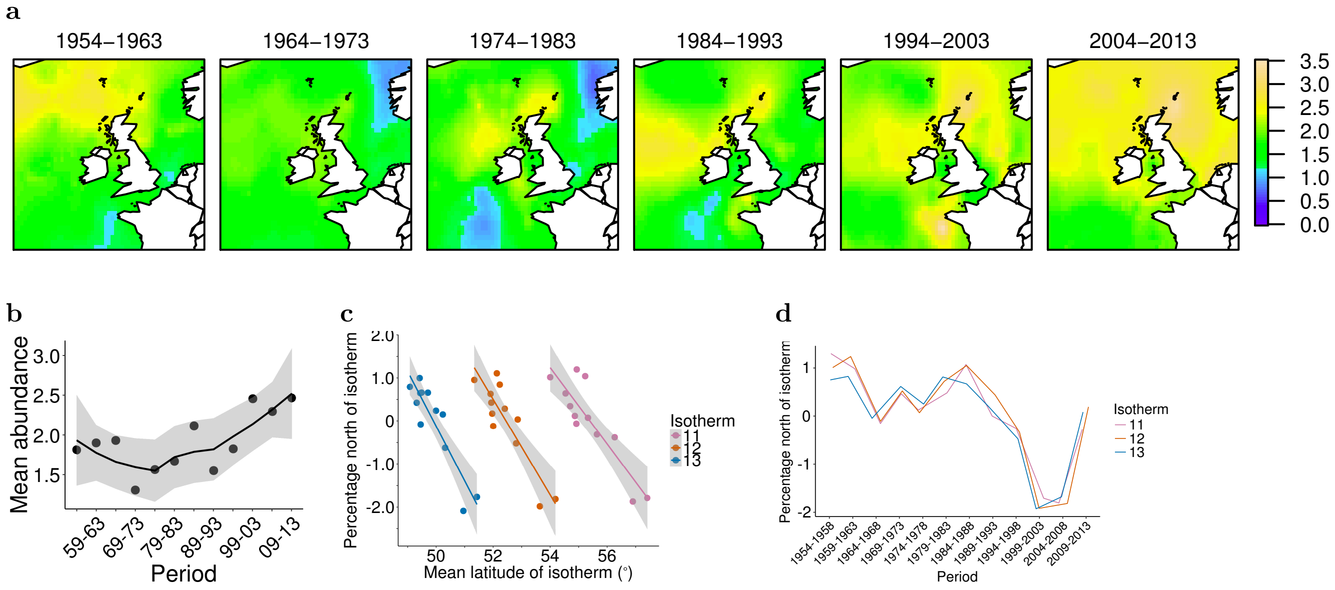

Figure 14: ***Thalassiosira spp.***

a, Mean cell count per sample ($log(x + 1)$) of combined pairs of five-year periods, 45–64°N, 20°W–8°E. The scale illustrates temporal and spatial abundance.

b, Abundance: the mean of the values derived by kriging at each (longitude, latitude) for each five-year period (and used to create the maps above). A loess smoother was used for locally weighted polynomial regression, the grey area indicating the 95% confidence interval for the line. Descriptive statistics: max 2.4654, min 1.3121, mean 1.9102, SD 0.3656.

c, Proportions (normalised) of the taxon population north of isotherms at 11, 12 and 13°C within the geographic area 45–64°N, 20°W–8°E in the 12 five-year periods from 1954–1958 to 2009–2013. The isotherms move north as the SST rises; these plots illustrate correlations between the normalised population percentages north of each isotherm and the mean latitude of the isotherms. A loess smoother was used for locally weighted polynomial regression, the grey area indicating the 95% confidence interval for the line. The p-values are listed in Supplementary Table 1b.

d, Normalised percentage of the taxonomic group north of three isotherms over time. Negative correlation between the percentage north of an isotherm and the isotherm latitude indicates niche plasticity in relation to thermal change, so a falling percentage in this graph in the period 1984–2008 (during which there was constant warming) suggests niche plasticity.

2.11 *Thalassiothrix longissima*

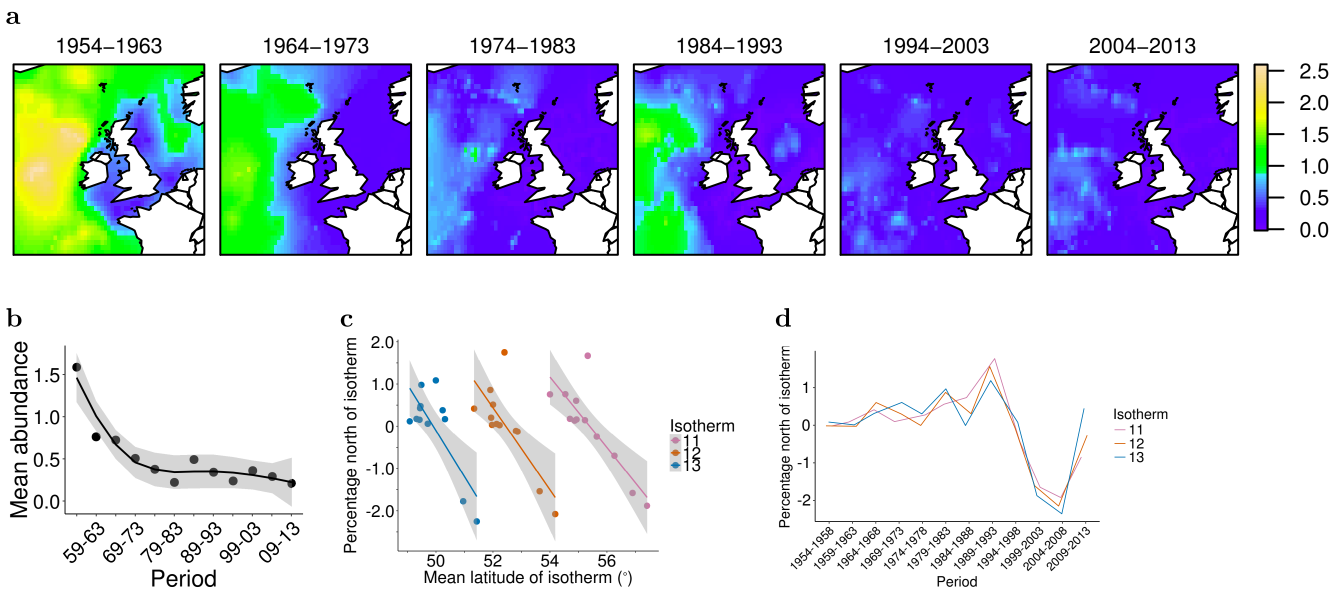

Figure 15: *Thalassiothrix longissima*.

a, Mean cell count per sample ($log(x + 1)$) of combined pairs of five-year periods, 45–64°N, 20°W–8°E. The scale illustrates temporal and spatial abundance.

b, Abundance: the mean of the values derived by kriging at each (longitude, latitude) for each five-year period (and used to create the maps above). A loess smoother was used for locally weighted polynomial regression, the grey area indicating the 95% confidence interval for the line. Descriptive statistics: max 1.5868, min 0.211, mean 0.5123, SD 0.3841.

c, Proportions (normalised) of the taxon population north of isotherms at 11, 12 and 13°C within the geographic area 45–64°N, 20°W–8°E in the 12 five-year periods from 1954–1958 to 2009–2013. The isotherms move north as the SST rises; these plots illustrate correlations between the normalised population percentages north of each isotherm and the mean latitude of the isotherms. A loess smoother was used for locally weighted polynomial regression, the grey area indicating the 95% confidence interval for the line. The *p*-values are listed in Supplementary Table 1b.

d, Normalised percentage of the taxonomic group north of three isotherms over time. Negative correlation between the percentage north of an isotherm and the isotherm latitude indicates niche plasticity in relation to thermal change, so a falling percentage in this graph in the period 1984–2008 (during which there was constant warming) suggests niche plasticity.

2.12 *Thalassionema nitzschioides*

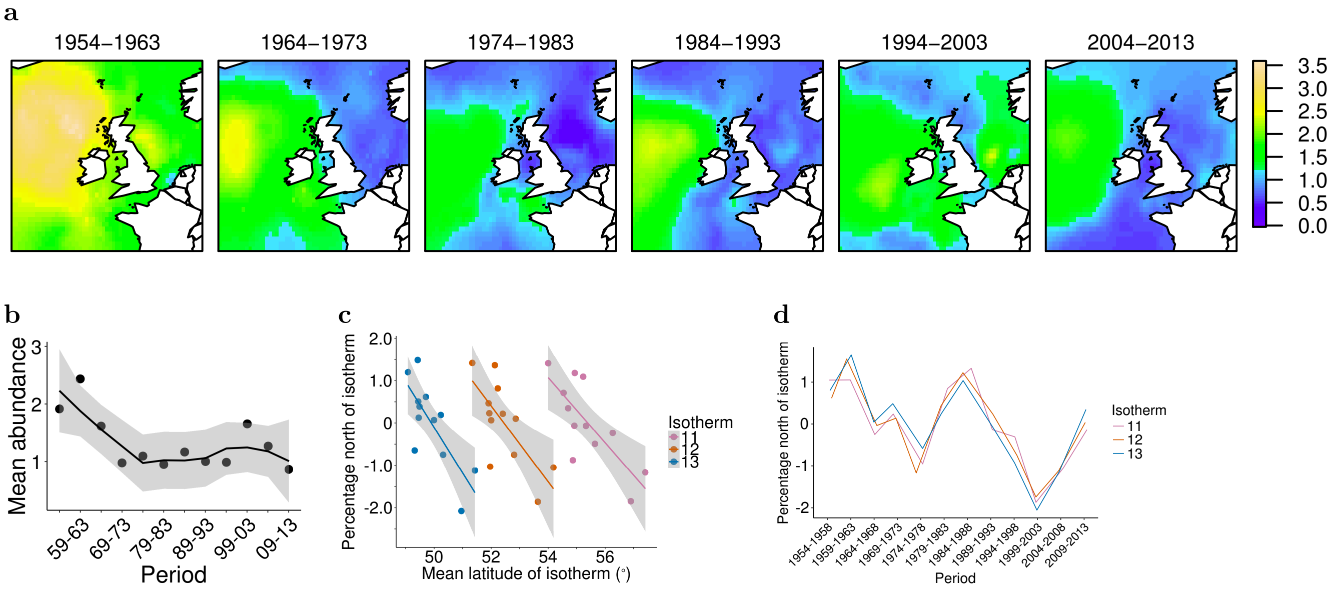

Figure 16: ***Thalassionema nitzschioides*.**

a, Mean cell count per sample ($log(x + 1)$) of combined pairs of five-year periods, 45–64°N, 20°W–8°E. The scale illustrates temporal and spatial abundance.

b, Abundance: the mean of the values derived by kriging at each (longitude, latitude) for each five-year period (and used to create the maps above). A loess smoother was used for locally weighted polynomial regression, the grey area indicating the 95% confidence interval for the line. Descriptive statistics: max 2.4307, min 0.8655, mean 1.3269, SD 0.4794.

c, Proportions (normalised) of the taxon population north of isotherms at 11, 12 and 13°C within the geographic area 45–64°N, 20°W–8°E in the 12 five-year periods from 1954–1958 to 2009–2013. The isotherms move north as the SST rises; these plots illustrate correlations between the normalised population percentages north of each isotherm and the mean latitude of the isotherms. A loess smoother was used for locally weighted polynomial regression, the grey area indicating the 95% confidence interval for the line. The p-values are listed in Supplementary Table 1b.

d, Normalised percentage of the taxonomic group north of three isotherms over time. Negative correlation between the percentage north of an isotherm and the isotherm latitude indicates niche plasticity in relation to thermal change, so a falling percentage in this graph in the period 1984–2008 (during which there was constant warming) suggests niche plasticity.

3 All Dinoflagellates—abundance maps and analysis

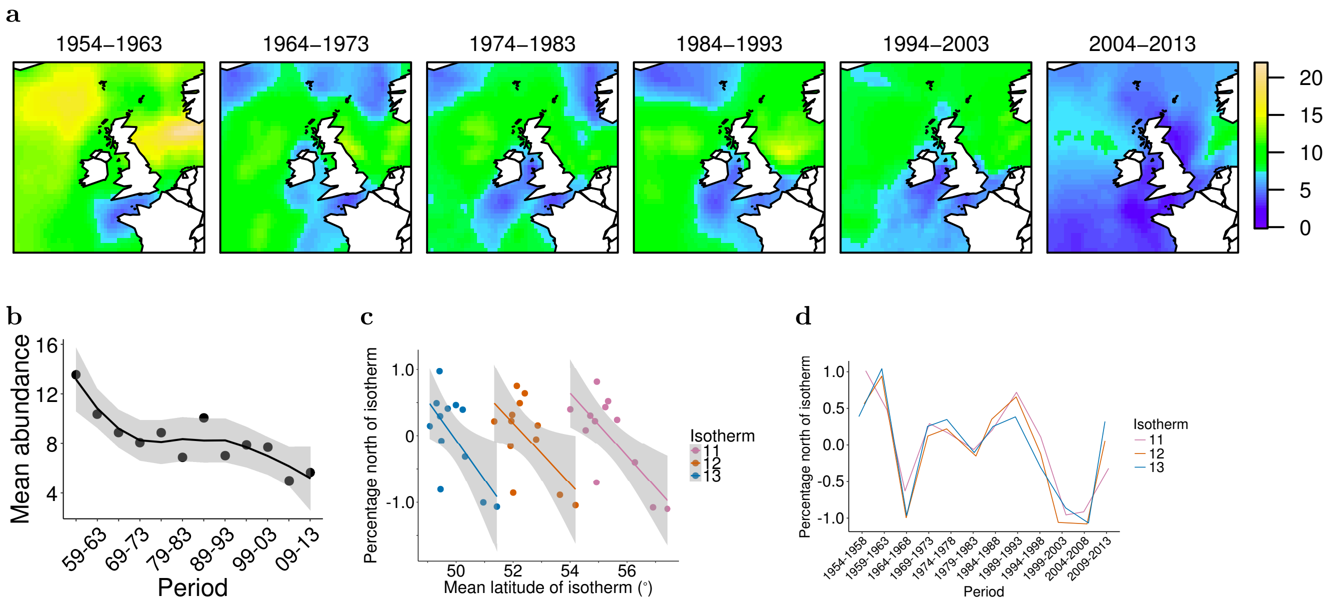

Figure 17: All 12 taxa of dinoflagellates.

a, Sum of the log-transformed ($log(x + 1)$) cell counts per (longitude, latitude) determined by ordinary kriging for each taxon in this group. The ten-year periods are the means of the five-year pairs. The geographic area is 45–64°N, 20°W–8°E, the scale illustrates temporal and spatial abundance.

b, Abundance: the sum of the means for each taxon of the values derived by kriging at each (longitude, latitude) for each five-year period (and used to create the maps above). A loess smoother was used for locally weighted polynomial regression, the grey area indicating the 95% confidence interval for the line.

c, Proportions (normalised) of the taxon population north of isotherms at 11, 12 and 13°C within the geographic area 45–64°N, 20°W–8°E in the 12 five-year periods from 1954–1958 to 2009–2013. The isotherms move north as the SST rises; these plots illustrate correlations between the normalised population percentages north of each isotherm and the mean latitude of the isotherms. A loess smoother was used for locally weighted polynomial regression, the grey area indicating the 95% confidence interval for the line. The p-values are listed in Supplementary Table 1a.

d, Normalised percentage of the combined taxonomic group north of three isotherms over time. Negative correlation between the percentage north of an isotherm and the isotherm latitude indicates niche plasticity in relation to thermal change, so a falling percentage in this graph in the period 1984–2008 (during which there was constant warming) suggests niche plasticity.

3.1 *Ceratium furca*

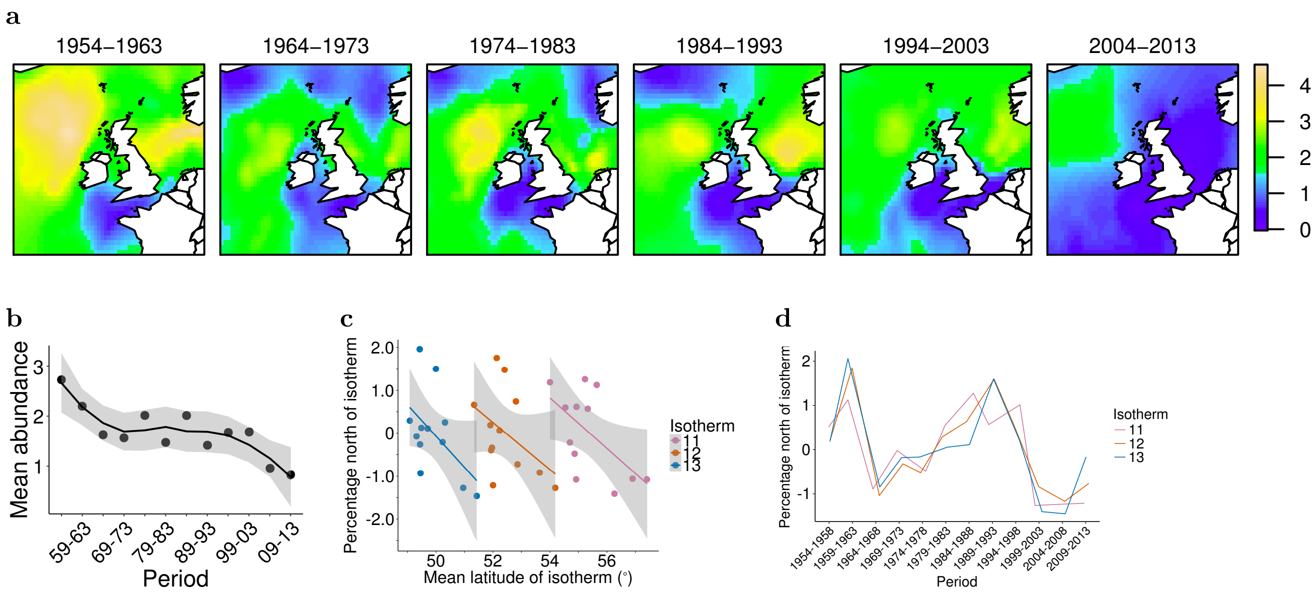

Figure 18: ***Ceratium furca*.**

a, Mean cell count per sample ($log(x + 1)$) of combined pairs of five-year periods, 45–64°N, 20°W–8°E. The scale illustrates temporal and spatial abundance.

b, Abundance: the mean of the values derived by kriging at each (longitude, latitude) for each five-year period (and used to create the maps above). A loess smoother was used for locally weighted polynomial regression, the grey area indicating the 95% confidence interval for the line. Descriptive statistics: max 2.7296, min 0.832, mean 1.6862, SD 0.5181.

c, Proportions (normalised) of the taxon population north of isotherms at 11, 12 and 13°C within the geographic area 45–64°N, 20°W–8°E in the 12 five-year periods from 1954–1958 to 2009–2013. The isotherms move north as the SST rises; these plots illustrate correlations between the normalised population percentages north of each isotherm and the mean latitude of the isotherms. A loess smoother was used for locally weighted polynomial regression, the grey area indicating the 95% confidence interval for the line. The *p*-values are listed in Supplementary Table 1b.

d, Normalised percentage of the taxonomic group north of three isotherms over time. Negative correlation between the percentage north of an isotherm and the isotherm latitude indicates niche plasticity in relation to thermal change, so a falling percentage in this graph in the period 1984–2008 (during which there was constant warming) suggests niche plasticity.

3.2 *Ceratium fusus*

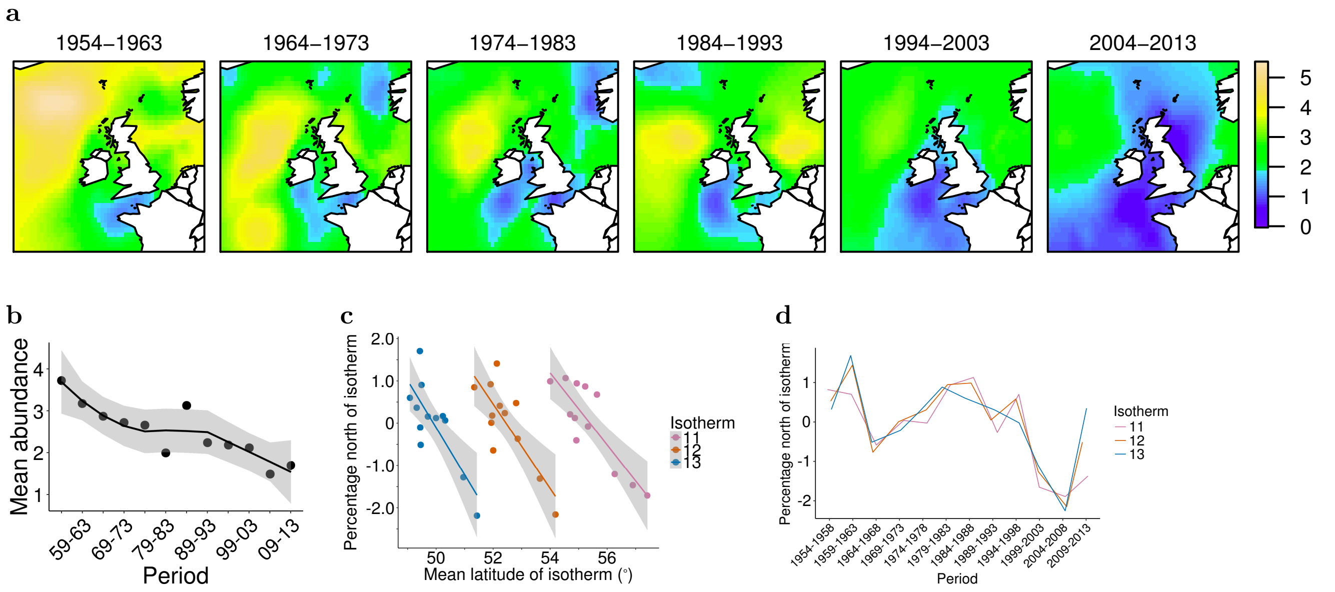

Figure 19: ***Ceratium fusus*****.**

a, Mean cell count per sample ($log(x + 1)$) of combined pairs of five-year periods, 45–64°N, 20°W–8°E. The scale illustrates temporal and spatial abundance.

b, Abundance: the mean of the values derived by kriging at each (longitude, latitude) for each five-year period (and used to create the maps above). A loess smoother was used for locally weighted polynomial regression, the grey area indicating the 95% confidence interval for the line. Descriptive statistics: max 3.72, min 1.4981, mean 2.5022, SD 0.6574.

c, Proportions (normalised) of the taxon population north of isotherms at 11, 12 and 13°C within the geographic area 45–64°N, 20°W–8°E in the 12 five-year periods from 1954–1958 to 2009–2013. The isotherms move north as the SST rises; these plots illustrate correlations between the normalised population percentages north of each isotherm and the mean latitude of the isotherms. A loess smoother was used for locally weighted polynomial regression, the grey area indicating the 95% confidence interval for the line. The p-values are listed in Supplementary Table 1b.

d, Normalised percentage of the taxonomic group north of three isotherms over time. Negative correlation between the percentage north of an isotherm and the isotherm latitude indicates niche plasticity in relation to thermal change, so a falling percentage in this graph in the period 1984–2008 (during which there was constant warming) suggests niche plasticity.

3.3 *Ceratium lineatum*

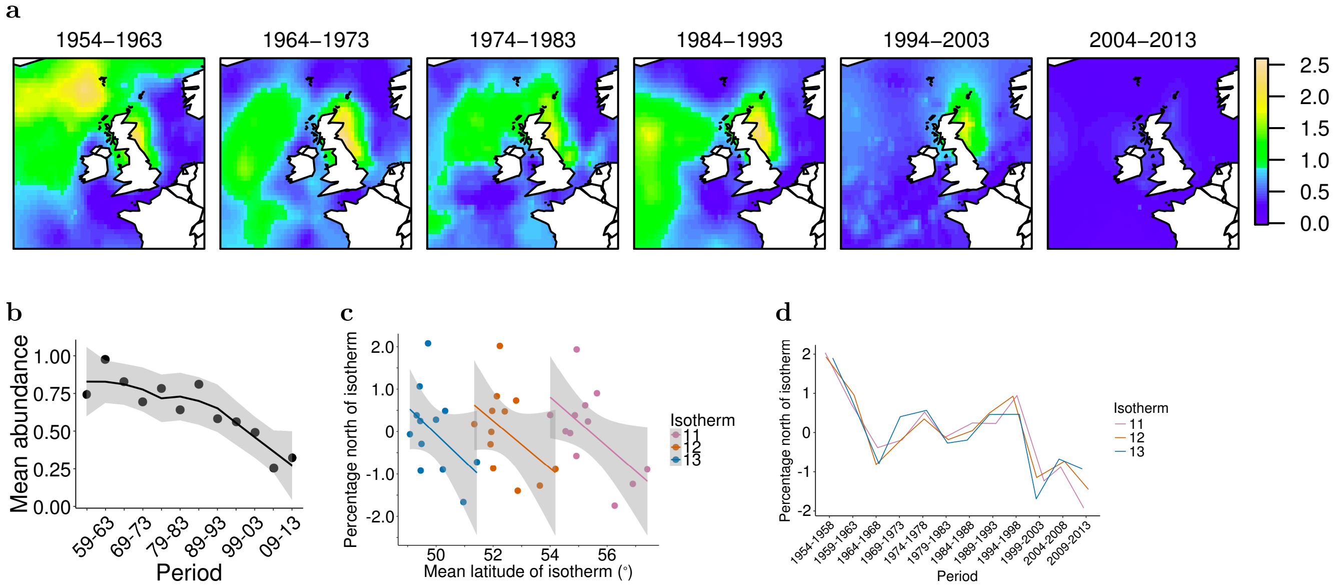

Figure 20: *Ceratium lineatum*.

a, Mean cell count per sample ($log(x + 1)$) of combined pairs of five-year periods, 45–64°N, 20°W–8°E. The scale illustrates temporal and spatial abundance.

b, Abundance: the mean of the values derived by kriging at each (longitude, latitude) for each five-year period (and used to create the maps above). A loess smoother was used for locally weighted polynomial regression, the grey area indicating the 95% confidence interval for the line. Descriptive statistics: max 0.9737, min 0.2551, mean 0.6399, SD 0.2109.

c, Proportions (normalised) of the taxon population north of isotherms at 11, 12 and 13°C within the geographic area 45–64°N, 20°W–8°E in the 12 five-year periods from 1954–1958 to 2009–2013. The isotherms move north as the SST rises; these plots illustrate correlations between the normalised population percentages north of each isotherm and the mean latitude of the isotherms. A loess smoother was used for locally weighted polynomial regression, the grey area indicating the 95% confidence interval for the line. The p-values are listed in Supplementary Table 1b.

d, Normalised percentage of the taxonomic group north of three isotherms over time. Negative correlation between the percentage north of an isotherm and the isotherm latitude indicates niche plasticity in relation to thermal change, so a falling percentage in this graph in the period 1984–2008 (during which there was constant warming) suggests niche plasticity.

3.4 *Ceratium tripos*

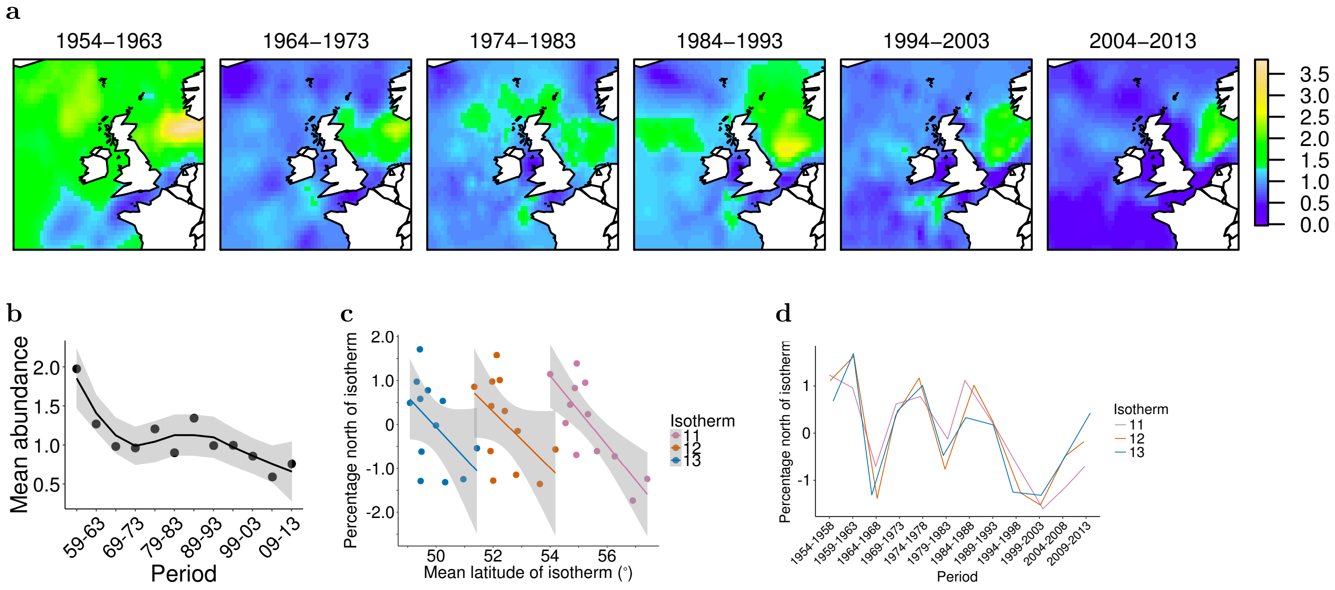

Figure 21: ***Ceratium tripos***.

a, Mean cell count per sample ($log(x + 1)$) of combined pairs of five-year periods, 45–64°N, 20°W–8°E. The scale illustrates temporal and spatial abundance.

b, Abundance: the mean of the values derived by kriging at each (longitude, latitude) for each five-year period (and used to create the maps above). A loess smoother was used for locally weighted polynomial regression, the grey area indicating the 95% confidence interval for the line. Descriptive statistics: max 1.9737, min 0.5958, mean 1.0716, SD 0.3533.

c, Proportions (normalised) of the taxon population north of isotherms at 11, 12 and 13°C within the geographic area 45–64°N, 20°W–8°E in the 12 five-year periods from 1954–1958 to 2009–2013. The isotherms move north as the SST rises; these plots illustrate correlations between the normalised population percentages north of each isotherm and the mean latitude of the isotherms. A loess smoother was used for locally weighted polynomial regression, the grey area indicating the 95% confidence interval for the line. The p-values are listed in Supplementary Table 1b.

d, Normalised percentage of the taxonomic group north of three isotherms over time. Negative correlation between the percentage north of an isotherm and the isotherm latitude indicates niche plasticity in relation to thermal change, so a falling percentage in this graph in the period 1984–2008 (during which there was constant warming) suggests niche plasticity.

3.5 *Ceratium macroceros*

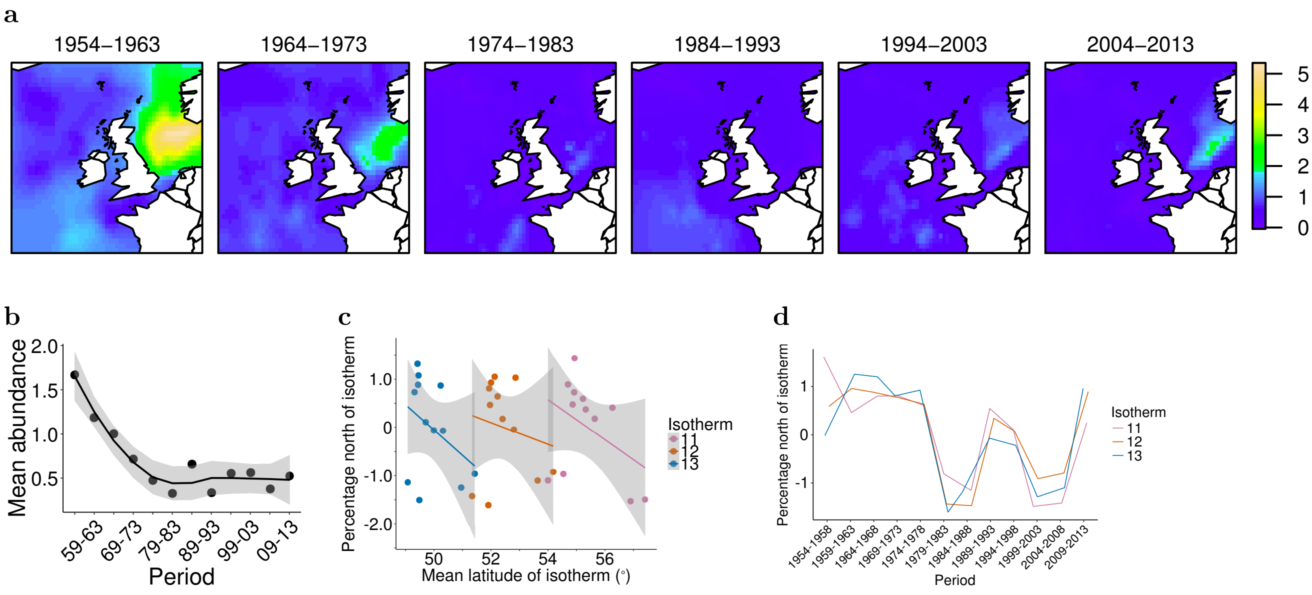

Figure 22: ***Ceratium macroceros*.**

a, Mean cell count per sample ($log(x + 1)$) of combined pairs of five-year periods, 45–64°N, 20°W–8°E. The scale illustrates temporal and spatial abundance.

b, Abundance: the mean of the values derived by kriging at each (longitude, latitude) for each five-year period (and used to create the maps above). A loess smoother was used for locally weighted polynomial regression, the grey area indicating the 95% confidence interval for the line. Descriptive statistics: max 1.6671, min 0.3288, mean 0.699, SD 0.3994.

c, Proportions (normalised) of the taxon population north of isotherms at 11, 12 and 13°C within the geographic area 45–64°N, 20°W–8°E in the 12 five-year periods from 1954–1958 to 2009–2013. The isotherms move north as the SST rises; these plots illustrate correlations between the normalised population percentages north of each isotherm and the mean latitude of the isotherms. A loess smoother was used for locally weighted polynomial regression, the grey area indicating the 95% confidence interval for the line. The p-values are listed in Supplementary Table 1b.

d, Normalised percentage of the taxonomic group north of three isotherms over time. Negative correlation between the percentage north of an isotherm and the isotherm latitude indicates niche plasticity in relation to thermal change, so a falling percentage in this graph in the period 1984–2008 (during which there was constant warming) suggests niche plasticity.

3.6 *Ceratium longipes*

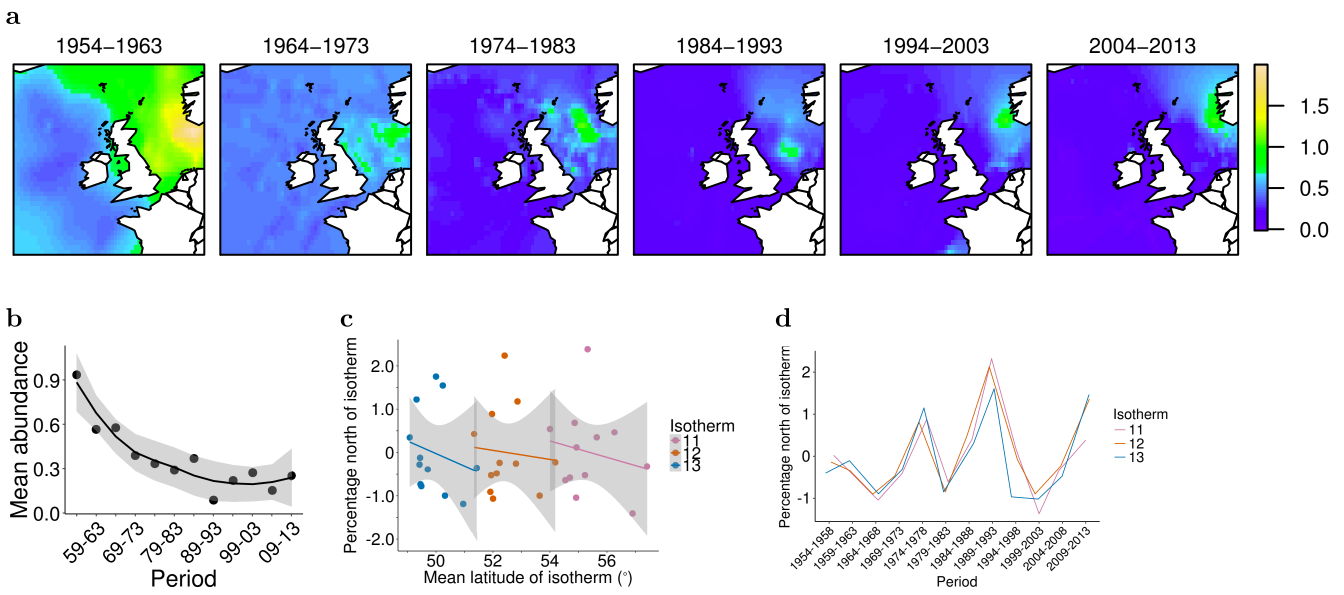

Figure 23: ***Ceratium longipes*.**

a, Mean cell count per sample ($log(x + 1)$) of combined pairs of five-year periods, 45–64°N, 20°W–8°E. The scale illustrates temporal and spatial abundance.

b, Abundance: the mean of the values derived by kriging at each (longitude, latitude) for each five-year period (and used to create the maps above). A loess smoother was used for locally weighted polynomial regression, the grey area indicating the 95% confidence interval for the line. Descriptive statistics: max 0.9342, min 0.0905, mean 0.3721, SD 0.2289.

c, Proportions (normalised) of the taxon population north of isotherms at 11, 12 and 13°C within the geographic area 45–64°N, 20°W–8°E in the 12 five-year periods from 1954–1958 to 2009–2013. The isotherms move north as the SST rises; these plots illustrate correlations between the normalised population percentages north of each isotherm and the mean latitude of the isotherms. A loess smoother was used for locally weighted polynomial regression, the grey area indicating the 95% confidence interval for the line. The p-values are listed in Supplementary Table 1b.

d, Normalised percentage of the taxonomic group north of three isotherms over time. Negative correlation between the percentage north of an isotherm and the isotherm latitude indicates niche plasticity in relation to thermal change, so a falling percentage in this graph in the period 1984–2008 (during which there was constant warming) suggests niche plasticity.

3.7 *Ceratium minutum*

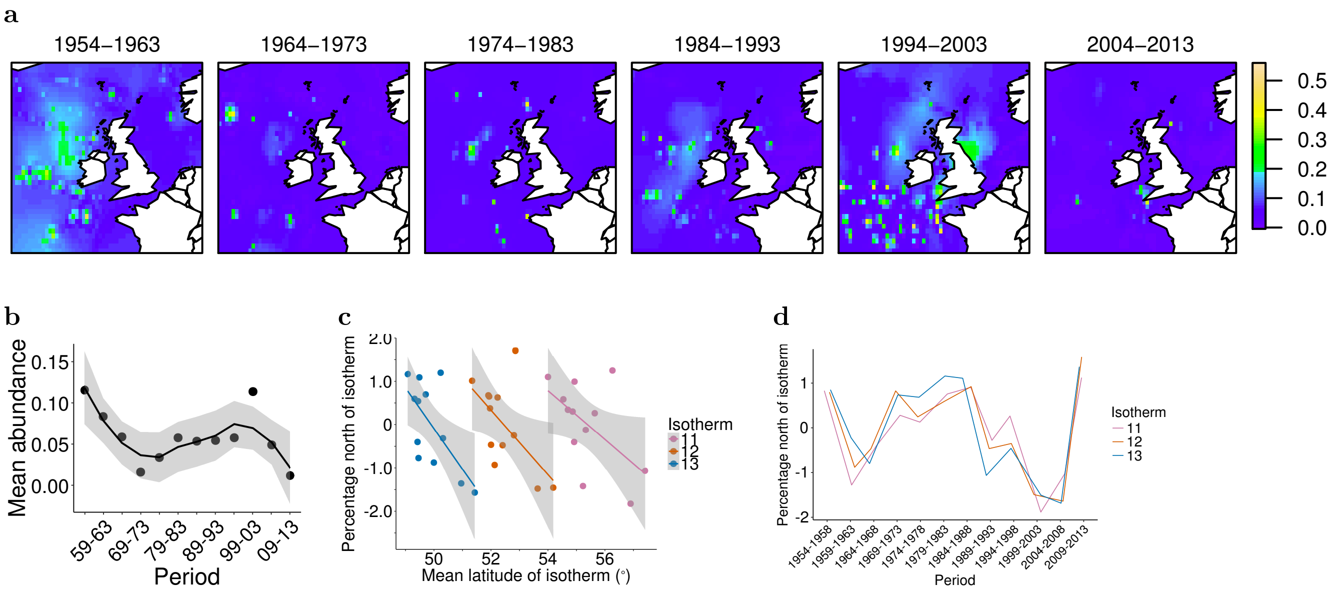

Figure 24: ***Ceratium minutum*.**

a, Mean cell count per sample ($log(x + 1)$) of combined pairs of five-year periods, 45–64°N, 20°W–8°E. The scale illustrates temporal and spatial abundance.

b, Abundance: the mean of the values derived by kriging at each (longitude, latitude) for each five-year period (and used to create the maps above). A loess smoother was used for locally weighted polynomial regression, the grey area indicating the 95% confidence interval for the line. Descriptive statistics: max 0.1153, min 0.0118, mean 0.0587, SD 0.0324.

c, Proportions (normalised) of the taxon population north of isotherms at 11, 12 and 13°C within the geographic area 45–64°N, 20°W–8°E in the 12 five-year periods from 1954–1958 to 2009–2013. The isotherms move north as the SST rises; these plots illustrate correlations between the normalised population percentages north of each isotherm and the mean latitude of the isotherms. A loess smoother was used for locally weighted polynomial regression, the grey area indicating the 95% confidence interval for the line. The p-values are listed in Supplementary Table 1b.

d, Normalised percentage of the taxonomic group north of three isotherms over time. Negative correlation between the percentage north of an isotherm and the isotherm latitude indicates niche plasticity in relation to thermal change, so a falling percentage in this graph in the period 1984–2008 (during which there was constant warming) suggests niche plasticity.

3.8 *Ceratium hexacanthum*

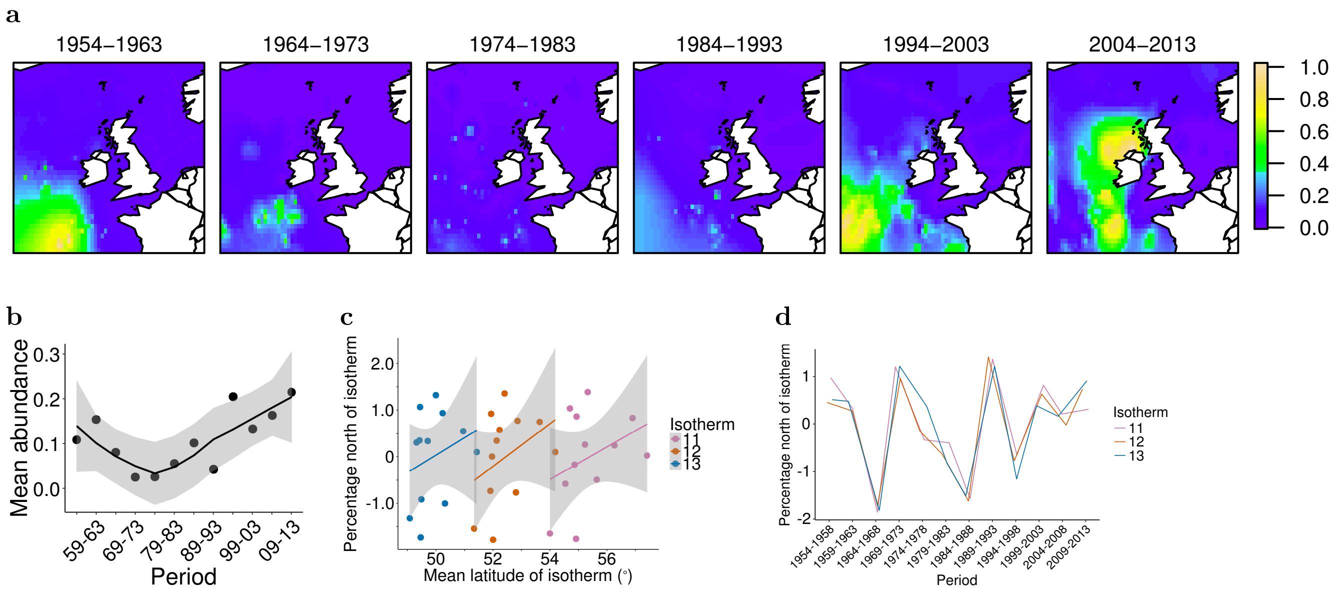

Figure 25: *Ceratium hexacanthum*.

a, Mean cell count per sample ($log(x + 1)$) of combined pairs of five-year periods, 45–64°N, 20°W–8°E. The scale illustrates temporal and spatial abundance.

b, Abundance: the mean of the values derived by kriging at each (longitude, latitude) for each five-year period (and used to create the maps above). A loess smoother was used for locally weighted polynomial regression, the grey area indicating the 95% confidence interval for the line. Descriptive statistics: max 0.2144, min 0.0253, mean 0.1088, SD 0.0657.

c, Proportions (normalised) of the taxon population north of isotherms at 11, 12 and 13°C within the geographic area 45–64°N, 20°W–8°E in the 12 five-year periods from 1954–1958 to 2009–2013. The isotherms move north as the SST rises; these plots illustrate correlations between the normalised population percentages north of each isotherm and the mean latitude of the isotherms. A loess smoother was used for locally weighted polynomial regression, the grey area indicating the 95% confidence interval for the line. The p-values are listed in Supplementary Table 1b.

d, Normalised percentage of the taxonomic group north of three isotherms over time. Negative correlation between the percentage north of an isotherm and the isotherm latitude indicates niche plasticity in relation to thermal change, so a falling percentage in this graph in the period 1984–2008 (during which there was constant warming) suggests niche plasticity.

3.9 *Dinophysis spp.*

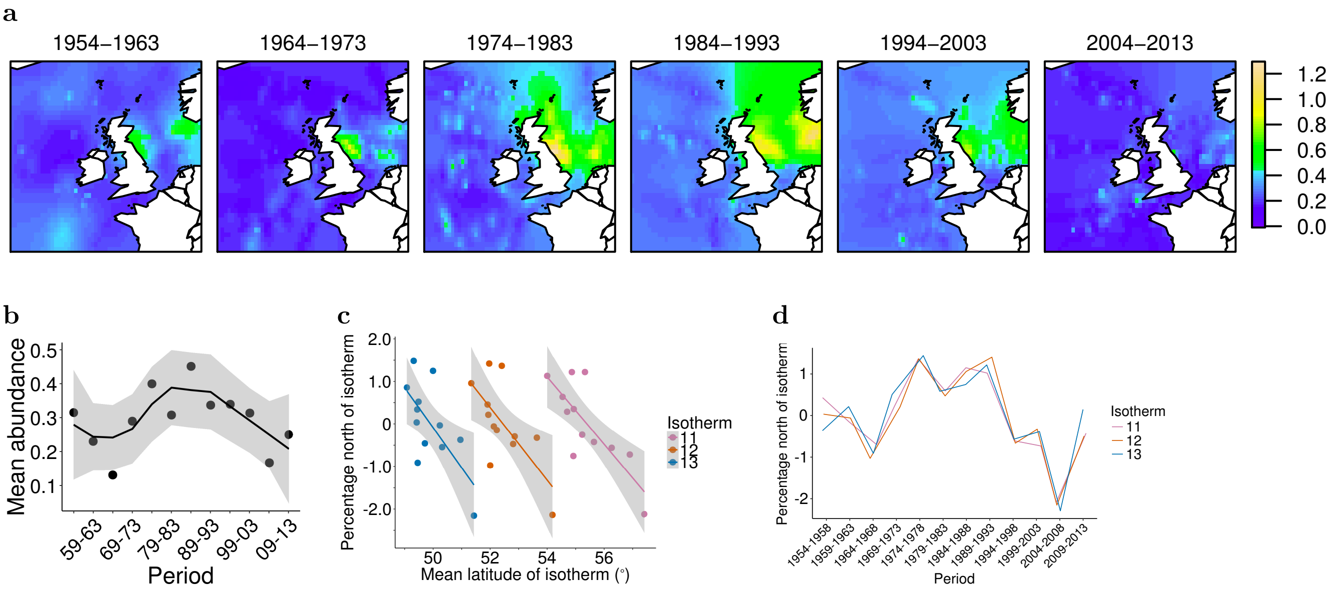

Figure 26: ***Dinophysis spp.***

a, Mean cell count per sample ($log(x + 1)$) of combined pairs of five-year periods, 45–64°N, 20°W–8°E. The scale illustrates temporal and spatial abundance.

b, Abundance: the mean of the values derived by kriging at each (longitude, latitude) for each five-year period (and used to create the maps above). A loess smoother was used for locally weighted polynomial regression, the grey area indicating the 95% confidence interval for the line. Descriptive statistics: max 0.451, min 0.1323, mean 0.2946, SD 0.0898.

c, Proportions (normalised) of the taxon population north of isotherms at 11, 12 and 13°C within the geographic area 45–64°N, 20°W–8°E in the 12 five-year periods from 1954–1958 to 2009–2013. The isotherms move north as the SST rises; these plots illustrate correlations between the normalised population percentages north of each isotherm and the mean latitude of the isotherms. A loess smoother was used for locally weighted polynomial regression, the grey area indicating the 95% confidence interval for the line. The p-values are listed in Supplementary Table 1b.

d, Normalised percentage of the taxonomic group north of three isotherms over time. Negative correlation between the percentage north of an isotherm and the isotherm latitude indicates niche plasticity in relation to thermal change, so a falling percentage in this graph in the period 1984–2008 (during which there was constant warming) suggests niche plasticity.

3.10 *Noctiluca scintillans*

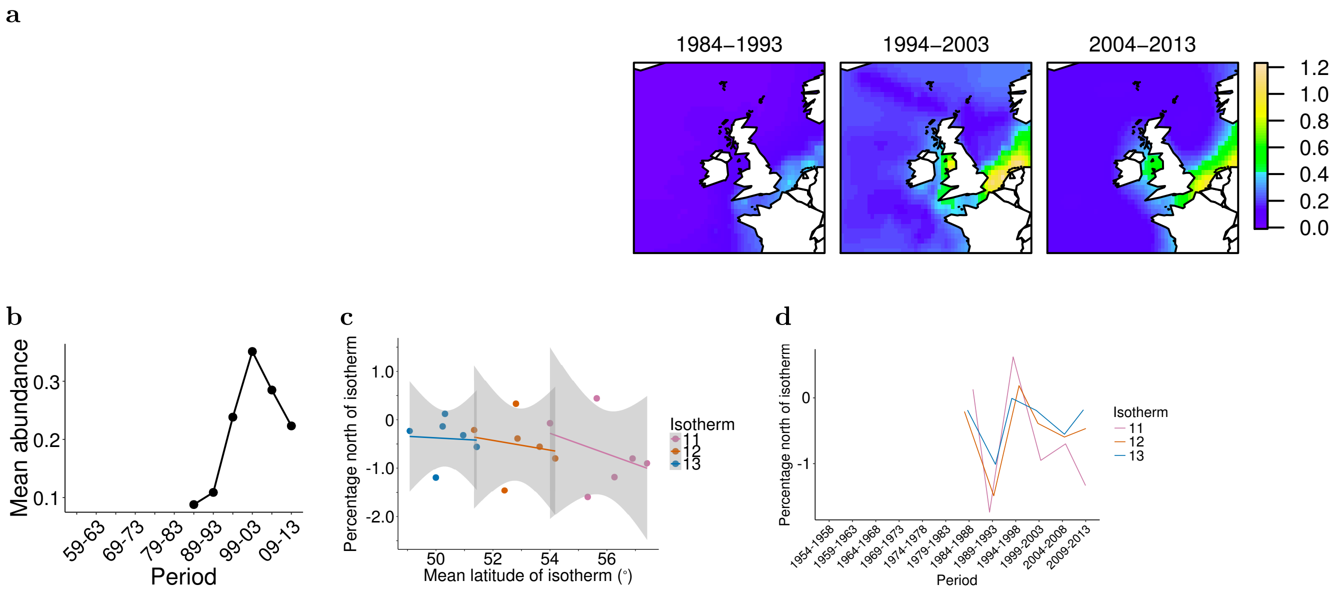

Figure 27: ***Noctiluca scintillans.*** Note that there were no data collected for this species before 1981.

a, Mean cell count per sample ($log(x + 1)$) of combined pairs of five-year periods, 45–64°N, 20°W–8°E. The scale illustrates temporal and spatial abundance.

b, Abundance: the mean of the values derived by kriging at each (longitude, latitude) for each five-year period (and used to create the maps above). A loess smoother was used for locally weighted polynomial regression, the grey area indicating the 95% confidence interval for the line. Descriptive statistics: max 0.3512, min 0.088, mean 0.2157, SD 0.1014.

c, Proportions (normalised) of the taxon population north of isotherms at 11, 12 and 13°C within the geographic area 45–64°N, 20°W–8°E in the 12 five-year periods from 1954–1958 to 2009–2013. The isotherms move north as the SST rises; these plots illustrate correlations between the normalised population percentages north of each isotherm and the mean latitude of the isotherms. A loess smoother was used for locally weighted polynomial regression, the grey area indicating the 95% confidence interval for the line. The p-values are listed in Supplementary Table 1b.

d, Normalised percentage of the taxonomic group north of three isotherms over time. Negative correlation between the percentage north of an isotherm and the isotherm latitude indicates niche plasticity in relation to thermal change, so a falling percentage in this graph in the period 1984–2008 (during which there was constant warming) suggests niche plasticity.

3.11 *Prorocentrum spp.*

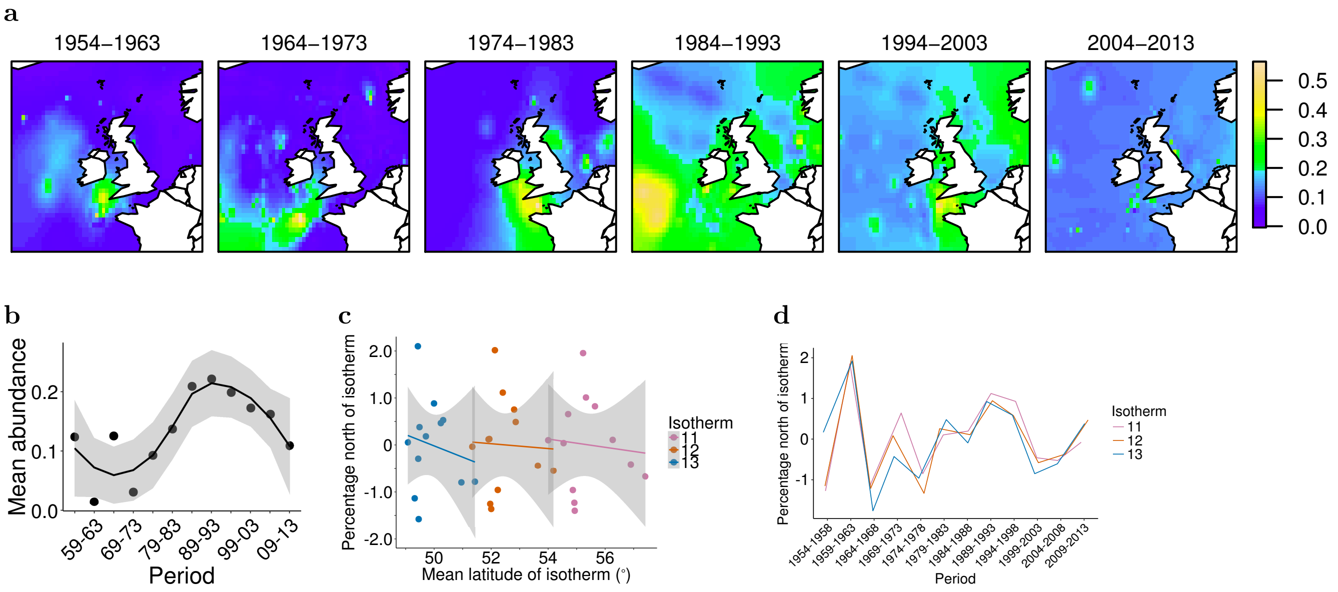

Figure 28: ***Prorocentrum spp.***

a, Mean cell count per sample ($log(x + 1)$) of combined pairs of five-year periods, 45–64°N, 20°W–8°E. The scale illustrates temporal and spatial abundance.

b, Abundance: the mean of the values derived by kriging at each (longitude, latitude) for each five-year period (and used to create the maps above). A loess smoother was used for locally weighted polynomial regression, the grey area indicating the 95% confidence interval for the line. Descriptive statistics: max 0.2215, min 0.0147, mean 0.1333, SD 0.0654.

c, Proportions (normalised) of the taxon population north of isotherms at 11, 12 and 13°C within the geographic area 45–64°N, 20°W–8°E in the 12 five-year periods from 1954–1958 to 2009–2013. The isotherms move north as the SST rises; these plots illustrate correlations between the normalised population percentages north of each isotherm and the mean latitude of the isotherms. A loess smoother was used for locally weighted polynomial regression, the grey area indicating the 95% confidence interval for the line. The p-values are listed in Supplementary Table 1b.

d, Normalised percentage of the taxonomic group north of three isotherms over time. Negative correlation between the percentage north of an isotherm and the isotherm latitude indicates niche plasticity in relation to thermal change, so a falling percentage in this graph in the period 1984–2008 (during which there was constant warming) suggests niche plasticity.

3.12 *Protoperidinium spp.*

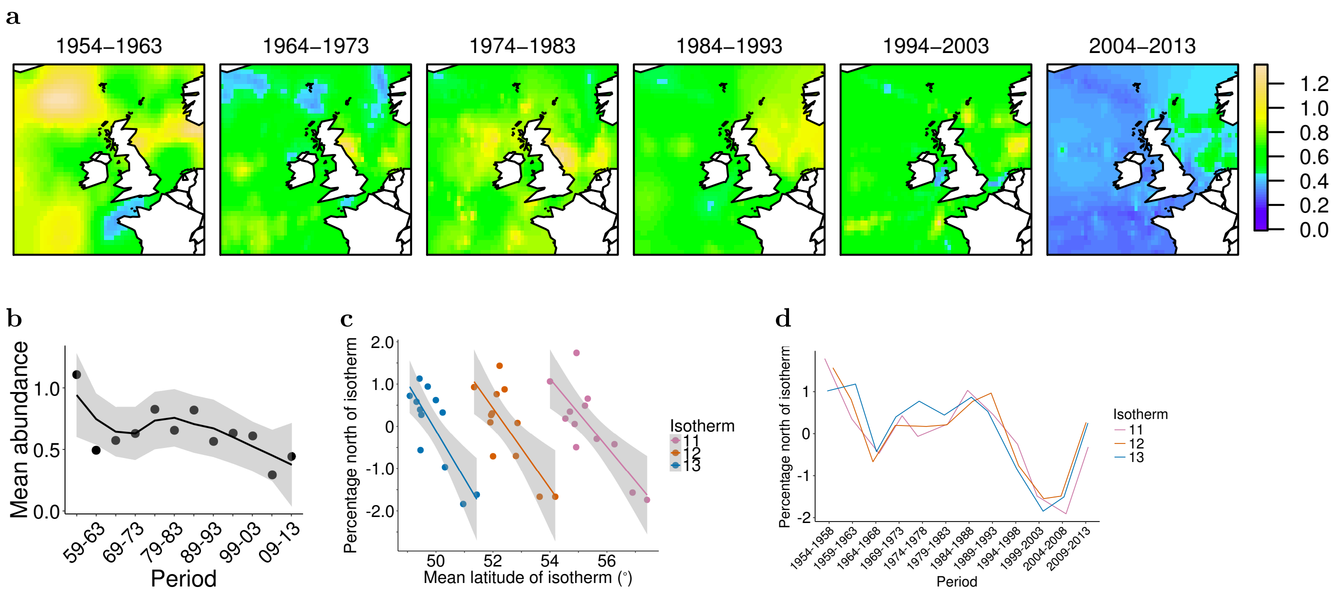

Figure 29: ***Protoperidinium spp.***

a, Mean cell count per sample ($log(x + 1)$) of combined pairs of five-year periods, 45–64°N, 20°W–8°E. The scale illustrates temporal and spatial abundance.

b, Abundance: the mean of the values derived by kriging at each (longitude, latitude) for each five-year period (and used to create the maps above). A loess smoother was used for locally weighted polynomial regression, the grey area indicating the 95% confidence interval for the line. Descriptive statistics: max 1.1074, min 0.2964, mean 0.6387, SD 0.2076.

c, Proportions (normalised) of the taxon population north of isotherms at 11, 12 and 13°C within the geographic area 45–64°N, 20°W–8°E in the 12 five-year periods from 1954–1958 to 2009–2013. The isotherms move north as the SST rises; these plots illustrate correlations between the normalised population percentages north of each isotherm and the mean latitude of the isotherms. A loess smoother was used for locally weighted polynomial regression, the grey area indicating the 95% confidence interval for the line. The p-values are listed in Supplementary Table 1b.

d, Normalised percentage of the taxonomic group north of three isotherms over time. Negative correlation between the percentage north of an isotherm and the isotherm latitude indicates niche plasticity in relation to thermal change, so a falling percentage in this graph in the period 1984–2008 (during which there was constant warming) suggests niche plasticity.

4 All *Calanus*, *Euchaeta* and *Undeuchaeta*—abundance maps and analysis

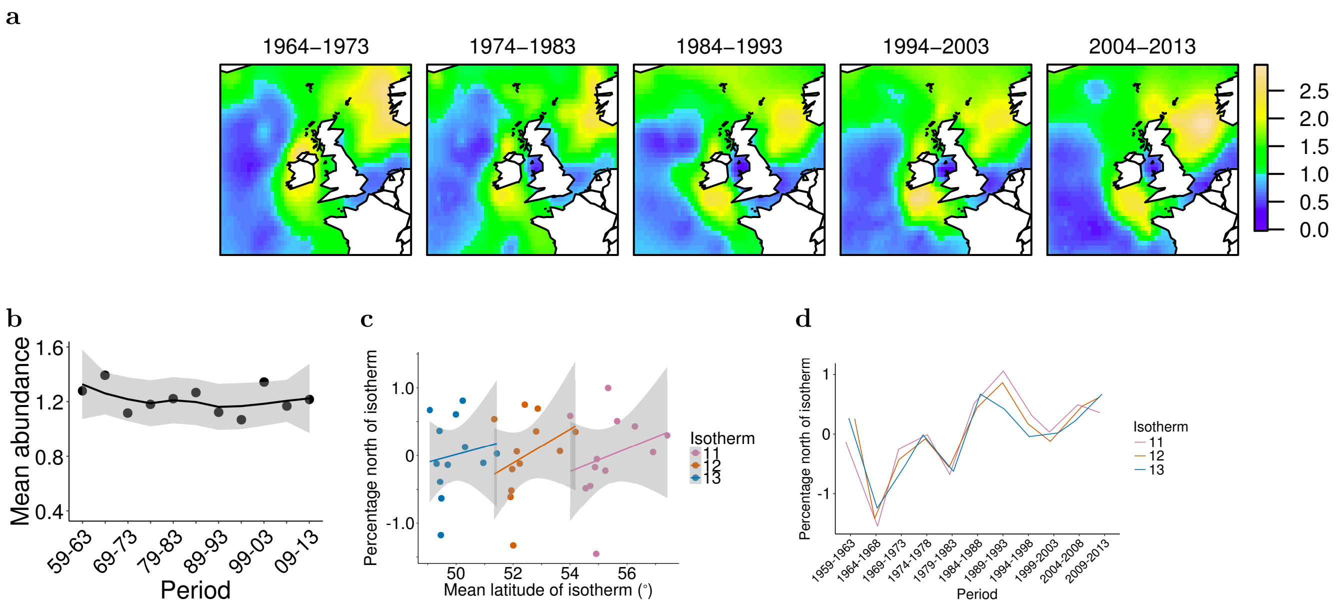

Figure 30: All 5 species of *Calanus*, *Euchaeta* and *Undeuchaeta*. Note that there were no data collected for the two individual *Calanus* species, by far the most populous of this taxonomic group (Supplementary Table 4), before 1958.

a, Sum of the log-transformed ($log(x + 1)$) cell counts per (longitude, latitude) determined by ordinary kriging for each taxon in this group. The ten-year periods are the means of the five-year pairs. The geographic area is 45–64°N, 20°W–8°E, the scale illustrates temporal and spatial abundance.

b, Abundance: the sum of the means for each taxon of the values derived by kriging at each (longitude, latitude) for each five-year period (and used to create the maps above). A loess smoother was used for locally weighted polynomial regression, the grey area indicating the 95% confidence interval for the line.

c, Proportions (normalised) of the taxon population north of isotherms at 11, 12 and 13°C within the geographic area 45–64°N, 20°W–8°E in the 12 five-year periods from 1954–1958 to 2009–2013. The isotherms move north as the SST rises; these plots illustrate correlations between the normalised population percentages north of each isotherm and the mean latitude of the isotherms. A loess smoother was used for locally weighted polynomial regression, the grey area indicating the 95% confidence interval for the line. The p-values are listed in Supplementary Table 1a.

d, Normalised percentage of the combined taxonomic group north of three isotherms over time. Negative correlation between the percentage north of an isotherm and the isotherm latitude indicates niche plasticity in relation to thermal change, so a falling percentage in this graph in the period 1984–2008 (during which there was constant warming) suggests niche plasticity.

4.1 *Calanus finmarchicus*

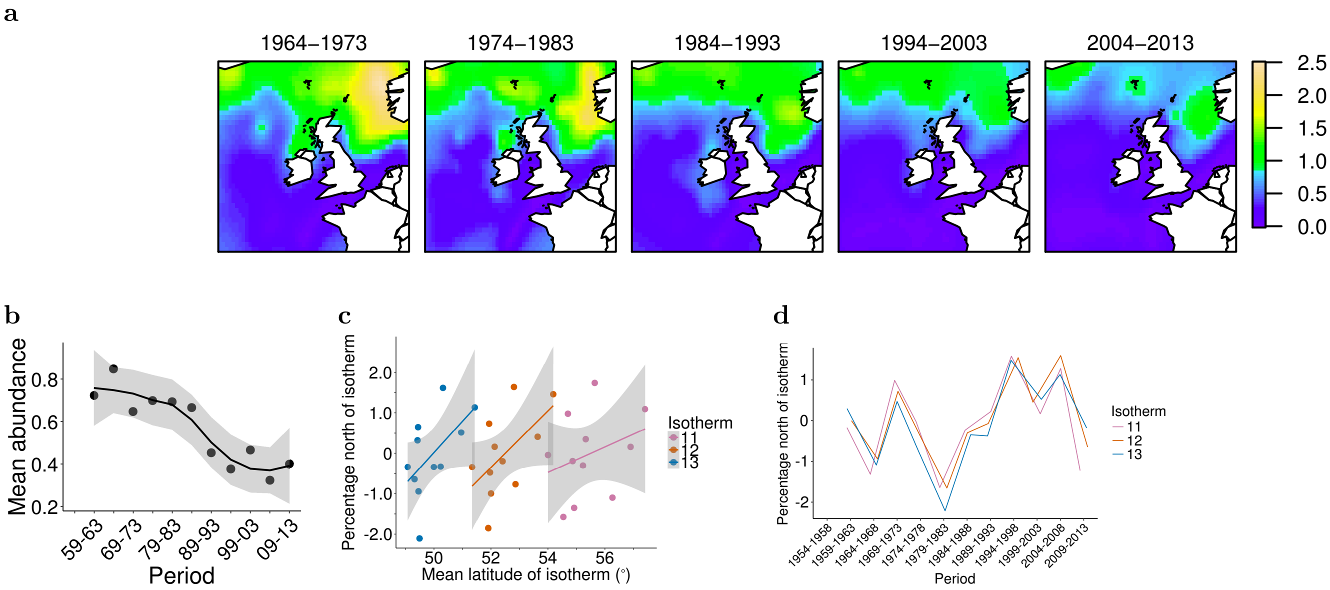

Figure 31: *Calanus finmarchicus*. Note that there were no data collected for this species before 1958.

a, Mean cell count per sample ($log(x + 1)$) of combined pairs of five-year periods, 45–64°N, 20°W–8°E. The scale illustrates temporal and spatial abundance.

b, Abundance: the mean of the values derived by kriging at each (longitude, latitude) for each five-year period (and used to create the maps above). A loess smoother was used for locally weighted polynomial regression, the grey area indicating the 95% confidence interval for the line. Descriptive statistics: max 0.8476, min 0.3258, mean 0.573, SD 0.1721.

c, Proportions (normalised) of the taxon population north of isotherms at 11, 12 and 13°C within the geographic area 45–64°N, 20°W–8°E in the 12 five-year periods from 1954–1958 to 2009–2013. The isotherms move north as the SST rises; these plots illustrate correlations between the normalised population percentages north of each isotherm and the mean latitude of the isotherms. A loess smoother was used for locally weighted polynomial regression, the grey area indicating the 95% confidence interval for the line. The *p*-values are listed in Supplementary Table 1b.

d, Normalised percentage of the taxonomic group north of three isotherms over time. Negative correlation between the percentage north of an isotherm and the isotherm latitude indicates niche plasticity in relation to thermal change, so a falling percentage in this graph in the period 1984–2008 (during which there was constant warming) suggests niche plasticity.

4.2 *Calanus helgolandicus*

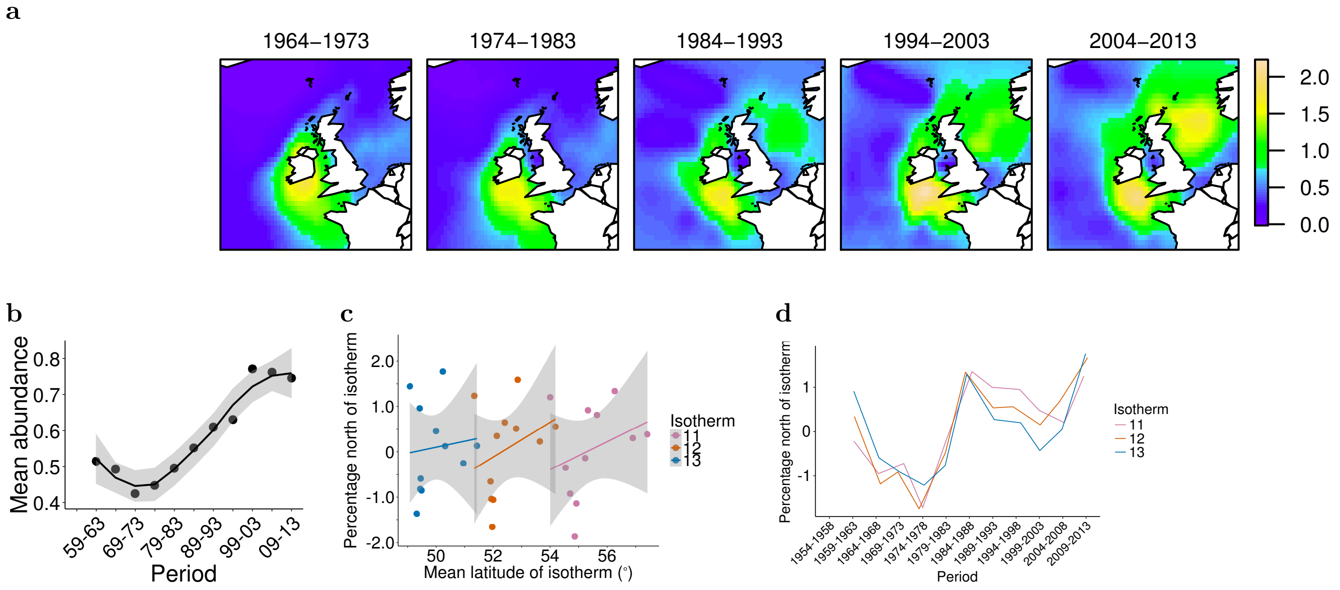

Figure 32: *Calanus helgolandicus*. Note that there were no data collected for this species before 1958.

a, Mean cell count per sample ($log(x + 1)$) of combined pairs of five-year periods, 45–64°N, 20°W–8°E. The scale illustrates temporal and spatial abundance.

b, Abundance: the mean of the values derived by kriging at each (longitude, latitude) for each five-year period (and used to create the maps above). A loess smoother was used for locally weighted polynomial regression, the grey area indicating the 95% confidence interval for the line. Descriptive statistics: max 0.771, min 0.4262, mean 0.5865, SD 0.1266.

c, Proportions (normalised) of the taxon population north of isotherms at 11, 12 and 13°C within the geographic area 45–64°N, 20°W–8°E in the 12 five-year periods from 1954–1958 to 2009–2013. The isotherms move north as the SST rises; these plots illustrate correlations between the normalised population percentages north of each isotherm and the mean latitude of the isotherms. A loess smoother was used for locally weighted polynomial regression, the grey area indicating the 95% confidence interval for the line. The p-values are listed in Supplementary Table 1b.

d, Normalised percentage of the taxonomic group north of three isotherms over time. Negative correlation between the percentage north of an isotherm and the isotherm latitude indicates niche plasticity in relation to thermal change, so a falling percentage in this graph in the period 1984–2008 (during which there was constant warming) suggests niche plasticity.

4.3 *Euchaeta acuta*

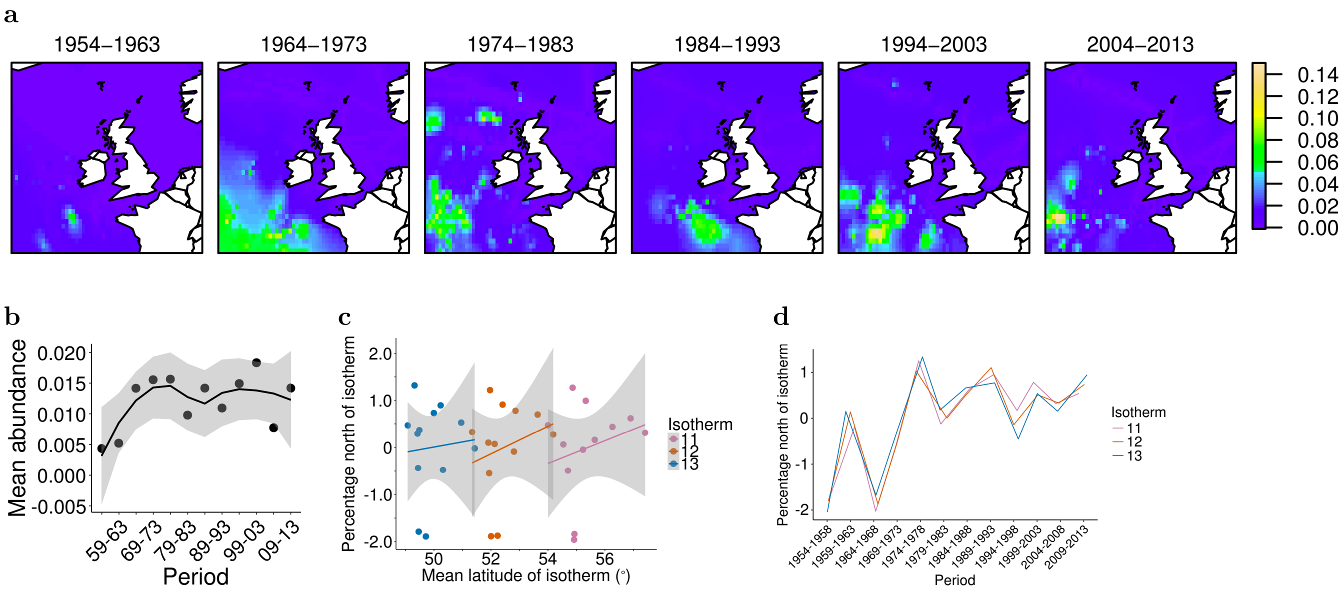

Figure 33: ***Euchaeta acuta*.**

a, Mean cell count per sample ($log(x + 1)$) of combined pairs of five-year periods, 45–64°N, 20°W–8°E. The scale illustrates temporal and spatial abundance.

b, Abundance: the mean of the values derived by kriging at each (longitude, latitude) for each five-year period (and used to create the maps above). A loess smoother was used for locally weighted polynomial regression, the grey area indicating the 95% confidence interval for the line. Descriptive statistics: max 0.0183, min 0.0043, mean 0.0121, SD 0.0044.

c, Proportions (normalised) of the taxon population north of isotherms at 11, 12 and 13°C within the geographic area 45–64°N, 20°W–8°E in the 12 five-year periods from 1954–1958 to 2009–2013. The isotherms move north as the SST rises; these plots illustrate correlations between the normalised population percentages north of each isotherm and the mean latitude of the isotherms. A loess smoother was used for locally weighted polynomial regression, the grey area indicating the 95% confidence interval for the line. The p-values are listed in Supplementary Table 1b.

d, Normalised percentage of the taxonomic group north of three isotherms over time. Negative correlation between the percentage north of an isotherm and the isotherm latitude indicates niche plasticity in relation to thermal change, so a falling percentage in this graph in the period 1984–2008 (during which there was constant warming) suggests niche plasticity.

4.4 *Euchaeta hebes*

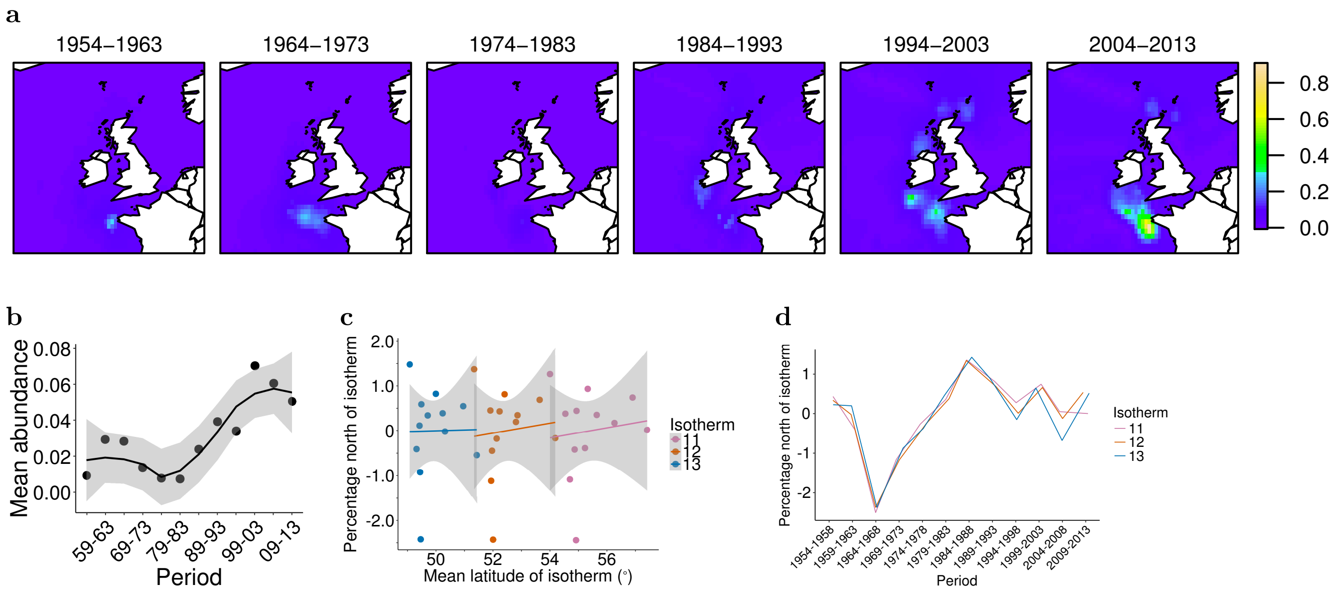

Figure 34: ***Euchaeta hebes*.**

a, Mean cell count per sample ($log(x + 1)$) of combined pairs of five-year periods, 45–64°N, 20°W–8°E. The scale illustrates temporal and spatial abundance.

b, Abundance: the mean of the values derived by kriging at each (longitude, latitude) for each five-year period (and used to create the maps above). A loess smoother was used for locally weighted polynomial regression, the grey area indicating the 95% confidence interval for the line. Descriptive statistics: max 0.0701, min 0.0074, mean 0.0311, SD 0.0208.

c, Proportions (normalised) of the taxon population north of isotherms at 11, 12 and 13°C within the geographic area 45–64°N, 20°W–8°E in the 12 five-year periods from 1954–1958 to 2009–2013. The isotherms move north as the SST rises; these plots illustrate correlations between the normalised population percentages north of each isotherm and the mean latitude of the isotherms. A loess smoother was used for locally weighted polynomial regression, the grey area indicating the 95% confidence interval for the line. The p-values are listed in Supplementary Table 1b.

d, Normalised percentage of the taxonomic group north of three isotherms over time. Negative correlation between the percentage north of an isotherm and the isotherm latitude indicates niche plasticity in relation to thermal change, so a falling percentage in this graph in the period 1984–2008 (during which there was constant warming) suggests niche plasticity.

4.5 *Undeuchaeta plumosa*

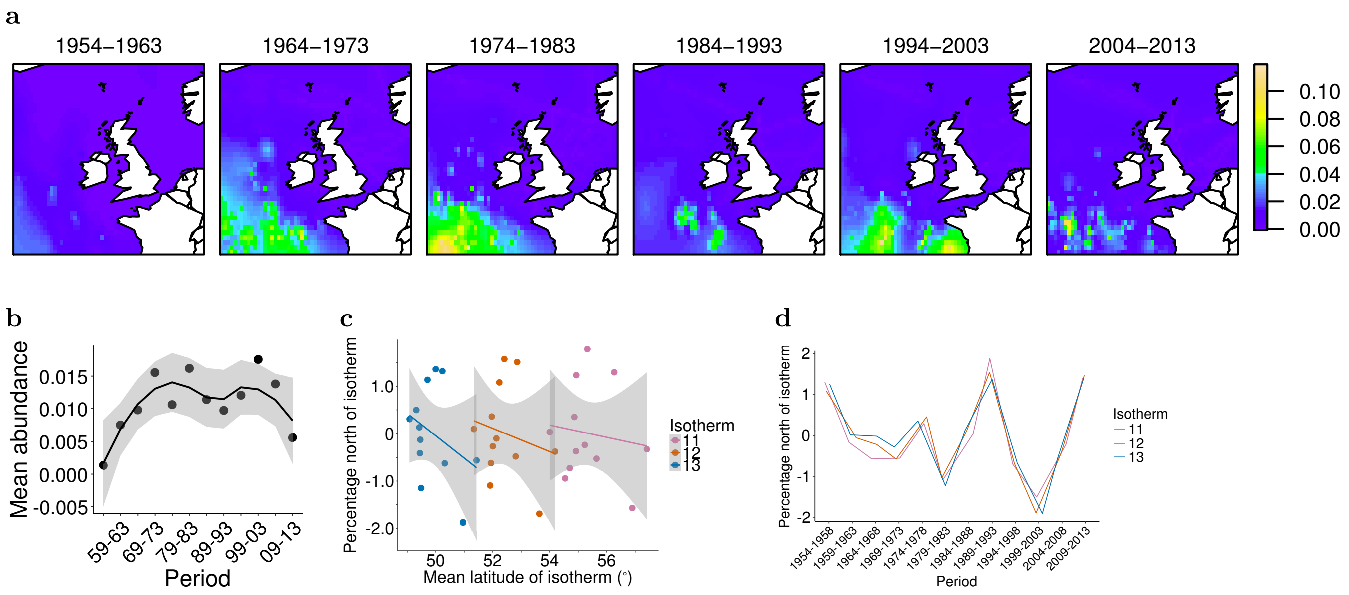

Figure 35: ***Undeuchaeta plumosa*.**

a, Mean cell count per sample ($log(x+1)$) of combined pairs of five-year periods, 45–64°N, 20°W–8°E. The scale illustrates temporal and spatial abundance.

b, Abundance: the mean of the values derived by kriging at each (longitude, latitude) for each five-year period (and used to create the maps above). A loess smoother was used for locally weighted polynomial regression, the grey area indicating the 95% confidence interval for the line. Descriptive statistics: max 0.0175, min 0.0014, mean 0.0109, SD 0.0046.

c, Proportions (normalised) of the taxon population north of isotherms at 11, 12 and 13°C within the geographic area 45–64°N, 20°W–8°E in the 12 five-year periods from 1954–1958 to 2009–2013. The isotherms move north as the SST rises; these plots illustrate correlations between the normalised population percentages north of each isotherm and the mean latitude of the isotherms. A loess smoother was used for locally weighted polynomial regression, the grey area indicating the 95% confidence interval for the line. The p-values are listed in Supplementary Table 1b.

d, Normalised percentage of the taxonomic group north of three isotherms over time. Negative correlation between the percentage north of an isotherm and the isotherm latitude indicates niche plasticity in relation to thermal change, so a falling percentage in this graph in the period 1984–2008 (during which there was constant warming) suggests niche plasticity.

5 All *Metridia* and *Pleuromamma*—abundance maps and analysis

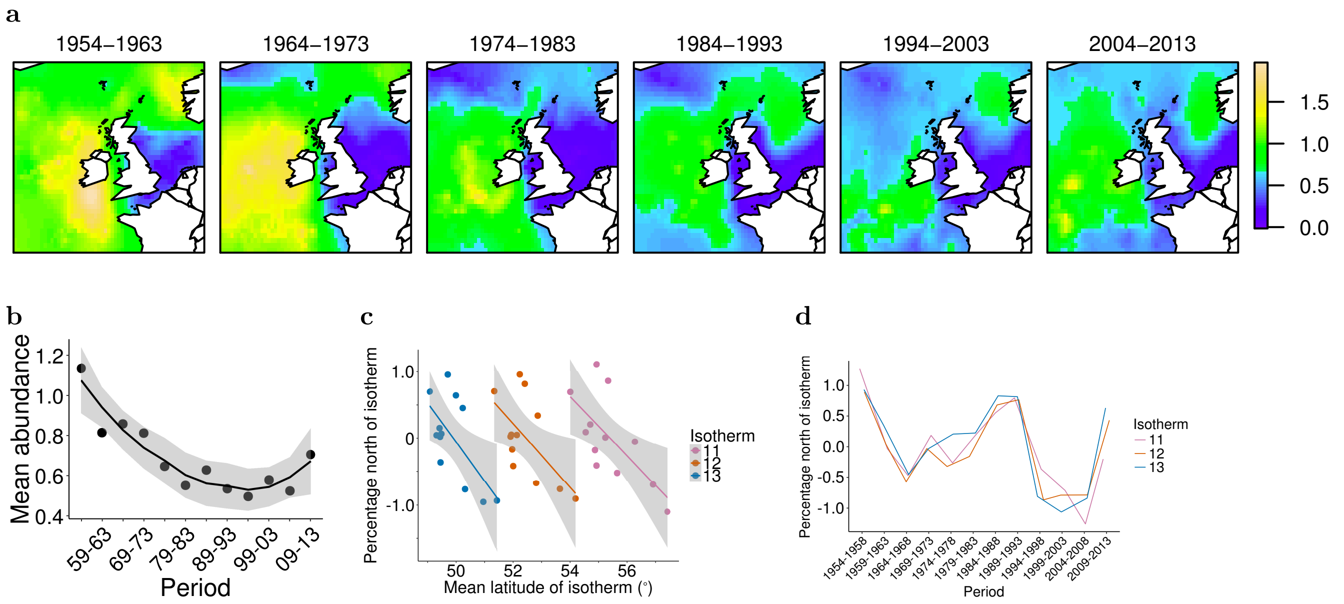

Figure 36: All 6 species of *Metridia* and *Pleuromamma*. Note that the data used were collected 6pm–6am only due to diel vertical migration.

a, Sum of the log-transformed ($log(x + 1)$) cell counts per (longitude, latitude) determined by ordinary kriging for each taxon in this group. The ten-year periods are the means of the five-year pairs. The geographic area is 45–64°N, 20°W–8°E, the scale illustrates temporal and spatial abundance.

b, Abundance: the sum of the means for each taxon of the values derived by kriging at each (longitude, latitude) for each five-year period (and used to create the maps above). A loess smoother was used for locally weighted polynomial regression, the grey area indicating the 95% confidence interval for the line.

c, Proportions (normalised) of the taxon population north of isotherms at 11, 12 and 13°C within the geographic area 45–64°N, 20°W–8°E in the 12 five-year periods from 1954–1958 to 2009–2013. The isotherms move north as the SST rises; these plots illustrate correlations between the normalised population percentages north of each isotherm and the mean latitude of the isotherms. A loess smoother was used for locally weighted polynomial regression, the grey area indicating the 95% confidence interval for the line. The p-values are listed in Supplementary Table 1a.

d, Normalised percentage of the combined taxonomic group north of three isotherms over time. Negative correlation between the percentage north of an isotherm and the isotherm latitude indicates niche plasticity in relation to thermal change, so a falling percentage in this graph in the period 1984–2008 (during which there was constant warming) suggests niche plasticity.

5.1 *Metridia longa*

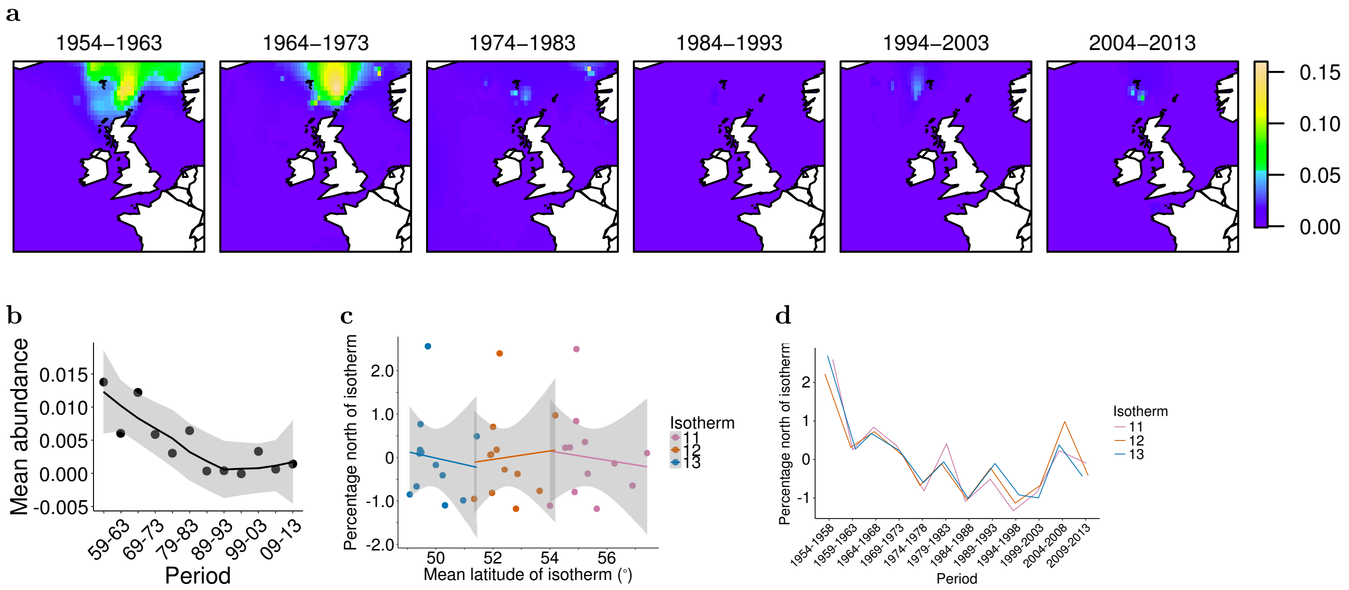

Figure 37: ***Metridia longa.*** Note that the data used were collected 6pm–6am only due to diel vertical migration.

a, Mean cell count per sample ($log(x+1)$) of combined pairs of five-year periods, 45–64°N, 20°W–8°E. The scale illustrates temporal and spatial abundance.

b, Abundance: the mean of the values derived by kriging at each (longitude, latitude) for each five-year period (and used to create the maps above). A loess smoother was used for locally weighted polynomial regression, the grey area indicating the 95% confidence interval for the line. Descriptive statistics: max 0.0138, min 0, mean 0.0045, SD 0.0046.

c, Proportions (normalised) of the taxon population north of isotherms at 11, 12 and 13°C within the geographic area 45–64°N, 20°W–8°E in the 12 five-year periods from 1954–1958 to 2009–2013. The isotherms move north as the SST rises; these plots illustrate correlations between the normalised population percentages north of each isotherm and the mean latitude of the isotherms. A loess smoother was used for locally weighted polynomial regression, the grey area indicating the 95% confidence interval for the line. The p-values are listed in Supplementary Table 1b.

d, Normalised percentage of the taxonomic group north of three isotherms over time. Negative correlation between the percentage north of an isotherm and the isotherm latitude indicates niche plasticity in relation to thermal change, so a falling percentage in this graph in the period 1984–2008 (during which there was constant warming) suggests niche plasticity.

5.2 *Metridia lucens*

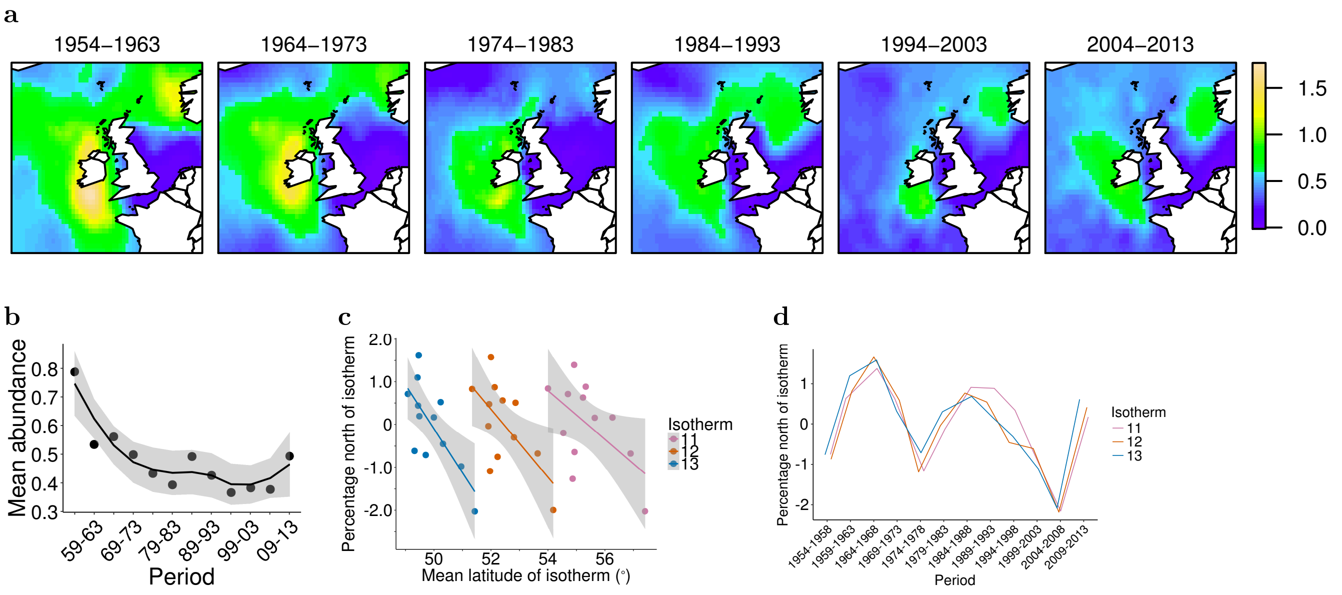

Figure 38: ***Metridia lucens.*** Note that the data used were collected 6pm–6am only due to diel vertical migration.

a, Mean cell count per sample ($log(x+1)$) of combined pairs of five-year periods, 45–64°N, 20°W–8°E. The scale illustrates temporal and spatial abundance.

b, Abundance: the mean of the values derived by kriging at each (longitude, latitude) for each five-year period (and used to create the maps above). A loess smoother was used for locally weighted polynomial regression, the grey area indicating the 95% confidence interval for the line. Descriptive statistics: max 0.7872, min 0.3667, mean 0.4789, SD 0.1167.

c, Proportions (normalised) of the taxon population north of isotherms at 11, 12 and 13°C within the geographic area 45–64°N, 20°W–8°E in the 12 five-year periods from 1954–1958 to 2009–2013. The isotherms move north as the SST rises; these plots illustrate correlations between the normalised population percentages north of each isotherm and the mean latitude of the isotherms. A loess smoother was used for locally weighted polynomial regression, the grey area indicating the 95% confidence interval for the line. The p-values are listed in Supplementary Table 1b.

d, Normalised percentage of the taxonomic group north of three isotherms over time. Negative correlation between the percentage north of an isotherm and the isotherm latitude indicates niche plasticity in relation to thermal change, so a falling percentage in this graph in the period 1984–2008 (during which there was constant warming) suggests niche plasticity.

5.3 *Pleuromamma abdominalis*

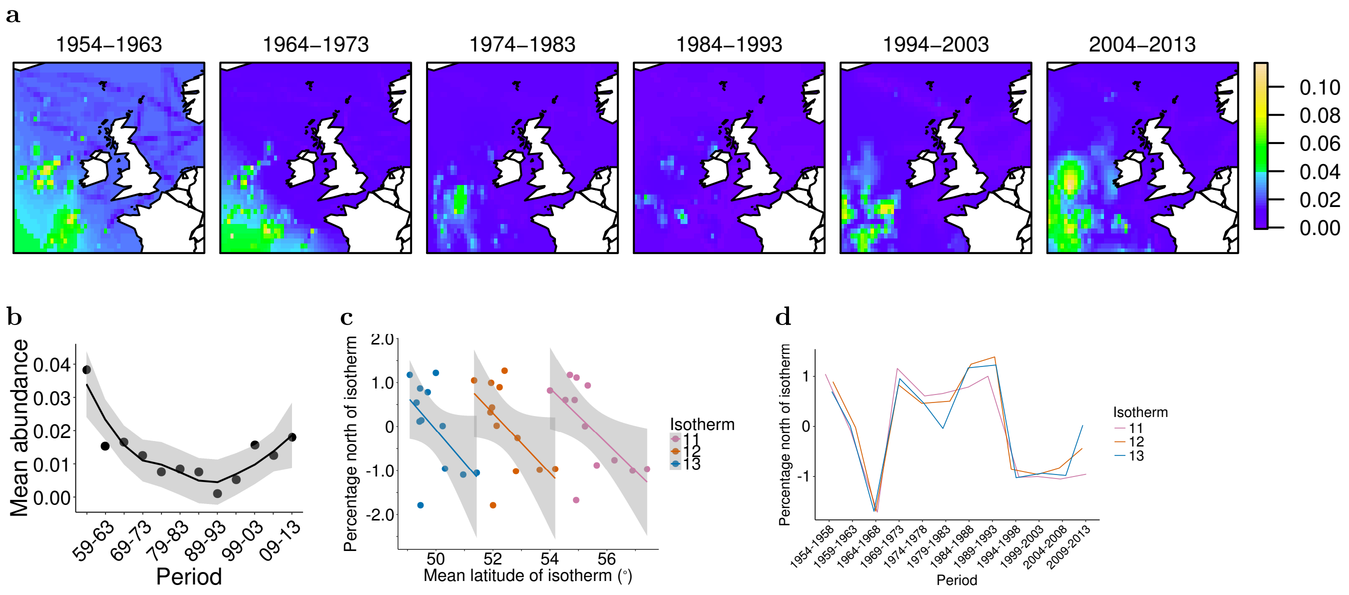

Figure 39: ***Pleuromamma abdominalis*.** Note that the data used were collected 6pm–6am only due to diel vertical migration.

a, Mean cell count per sample ($log(x + 1)$) of combined pairs of five-year periods, 45–64°N, 20°W–8°E. The scale illustrates temporal and spatial abundance.

b, Abundance: the mean of the values derived by kriging at each (longitude, latitude) for each five-year period (and used to create the maps above). A loess smoother was used for locally weighted polynomial regression, the grey area indicating the 95% confidence interval for the line. Descriptive statistics: max 0.0382, min 0.0011, mean 0.0132, SD 0.0094.

c, Proportions (normalised) of the taxon population north of isotherms at 11, 12 and 13°C within the geographic area 45–64°N, 20°W–8°E in the 12 five-year periods from 1954–1958 to 2009–2013. The isotherms move north as the SST rises; these plots illustrate correlations between the normalised population percentages north of each isotherm and the mean latitude of the isotherms. A loess smoother was used for locally weighted polynomial regression, the grey area indicating the 95% confidence interval for the line. The p-values are listed in Supplementary Table 1b.

d, Normalised percentage of the taxonomic group north of three isotherms over time. Negative correlation between the percentage north of an isotherm and the isotherm latitude indicates niche plasticity in relation to thermal change, so a falling percentage in this graph in the period 1984–2008 (during which there was constant warming) suggests niche plasticity.

5.4 *Pleuromamma borealis*

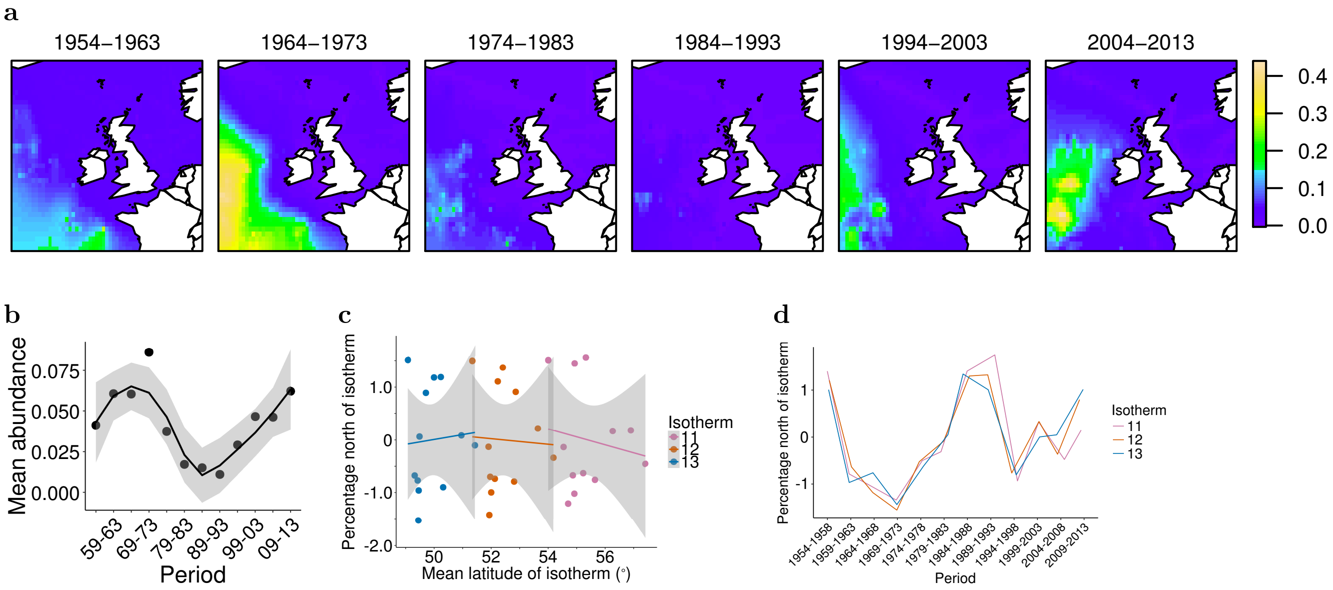

Figure 40: ***Pleuromamma borealis.*** Note that the data used were collected 6pm–6am only due to diel vertical migration.

a, Mean cell count per sample ($log(x+1)$) of combined pairs of five-year periods, 45–64°N, 20°W–8°E. The scale illustrates temporal and spatial abundance.

b, Abundance: the mean of the values derived by kriging at each (longitude, latitude) for each five-year period (and used to create the maps above). A loess smoother was used for locally weighted polynomial regression, the grey area indicating the 95% confidence interval for the line. Descriptive statistics: max 0.0859, min 0.0111, mean 0.0427, SD 0.0224.

c, Proportions (normalised) of the taxon population north of isotherms at 11, 12 and 13°C within the geographic area 45–64°N, 20°W–8°E in the 12 five-year periods from 1954–1958 to 2009–2013. The isotherms move north as the SST rises; these plots illustrate correlations between the normalised population percentages north of each isotherm and the mean latitude of the isotherms. A loess smoother was used for locally weighted polynomial regression, the grey area indicating the 95% confidence interval for the line. The p-values are listed in Supplementary Table 1b.

d, Normalised percentage of the taxonomic group north of three isotherms over time. Negative correlation between the percentage north of an isotherm and the isotherm latitude indicates niche plasticity in relation to thermal change, so a falling percentage in this graph in the period 1984–2008 (during which there was constant warming) suggests niche plasticity.

5.5 *Pleuromamma gracilis*

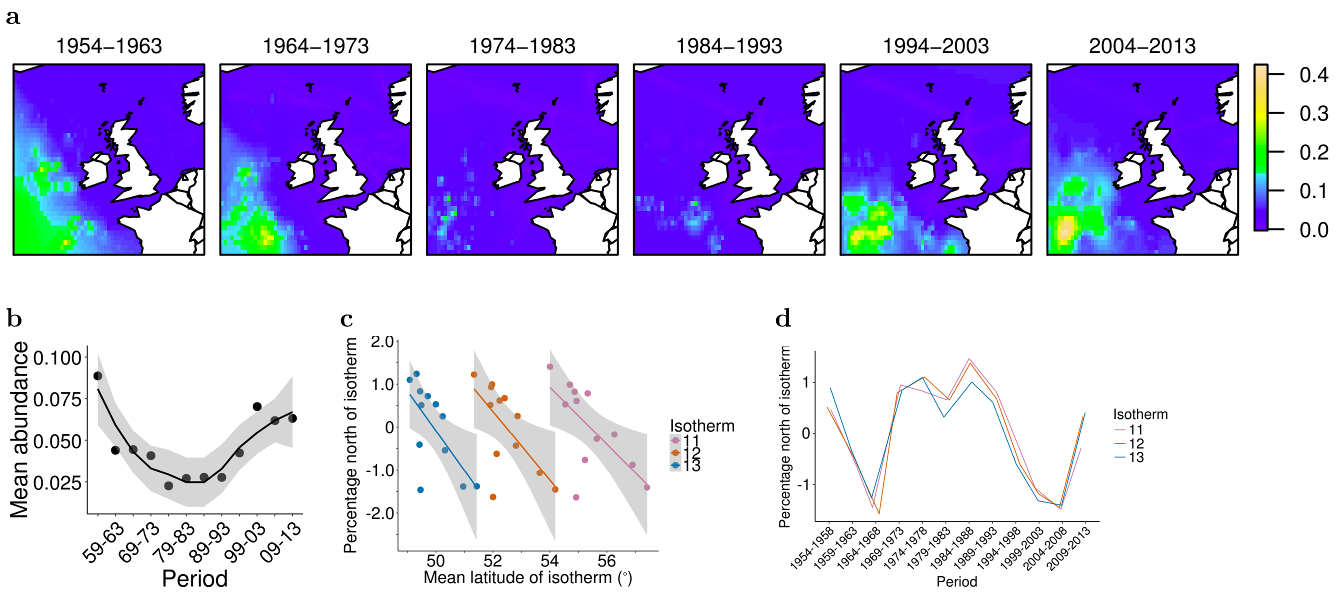

Figure 41: ***Pleuromamma gracilis.*** Note that the data used were collected 6pm–6am only due to diel vertical migration.

a, Mean cell count per sample ($log(x + 1)$) of combined pairs of five-year periods, 45–64°N, 20°W–8°E. The scale illustrates temporal and spatial abundance.

b, Abundance: the mean of the values derived by kriging at each (longitude, latitude) for each five-year period (and used to create the maps above). A loess smoother was used for locally weighted polynomial regression, the grey area indicating the 95% confidence interval for the line. Descriptive statistics: max 0.0887, min 0.0228, mean 0.0468, SD 0.0203.

c, Proportions (normalised) of the taxon population north of isotherms at 11, 12 and 13°C within the geographic area 45–64°N, 20°W–8°E in the 12 five-year periods from 1954–1958 to 2009–2013. The isotherms move north as the SST rises; these plots illustrate correlations between the normalised population percentages north of each isotherm and the mean latitude of the isotherms. A loess smoother was used for locally weighted polynomial regression, the grey area indicating the 95% confidence interval for the line. The p-values are listed in Supplementary Table 1b.

d, Normalised percentage of the taxonomic group north of three isotherms over time. Negative correlation between the percentage north of an isotherm and the isotherm latitude indicates niche plasticity in relation to thermal change, so a falling percentage in this graph in the period 1984–2008 (during which there was constant warming) suggests niche plasticity.

5.6 *Pleuromamma robusta*

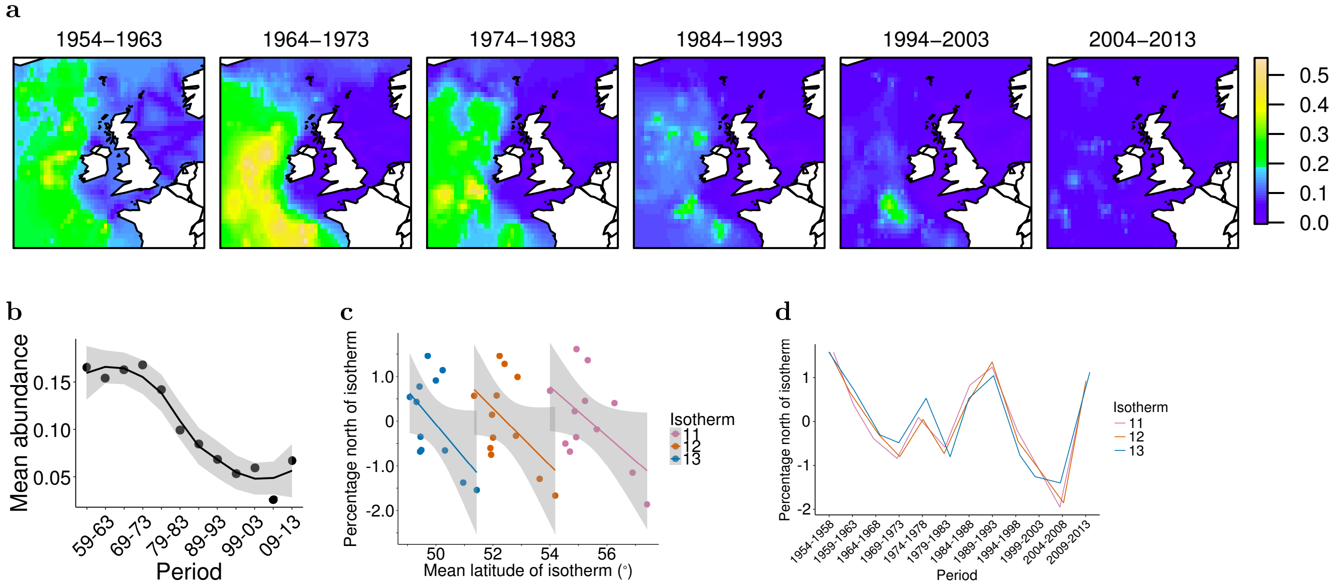

Figure 42: ***Pleuromamma robusta.*** Note that the data used were collected 6pm–6am only due to diel vertical migration.

a, Mean cell count per sample ($log(x + 1)$) of combined pairs of five-year periods, 45–64°N, 20°W–8°E. The scale illustrates temporal and spatial abundance.

b, Abundance: the mean of the values derived by kriging at each (longitude, latitude) for each five-year period (and used to create the maps above). A loess smoother was used for locally weighted polynomial regression, the grey area indicating the 95% confidence interval for the line. Descriptive statistics: max 0.1676, min 0.0259, mean 0.1041, SD 0.0512.

c, Proportions (normalised) of the taxon population north of isotherms at 11, 12 and 13°C within the geographic area 45–64°N, 20°W–8°E in the 12 five-year periods from 1954–1958 to 2009–2013. The isotherms move north as the SST rises; these plots illustrate correlations between the normalised population percentages north of each isotherm and the mean latitude of the isotherms. A loess smoother was used for locally weighted polynomial regression, the grey area indicating the 95% confidence interval for the line. The p-values are listed in Supplementary Table 1b.

d, Normalised percentage of the taxonomic group north of three isotherms over time. Negative correlation between the percentage north of an isotherm and the isotherm latitude indicates niche plasticity in relation to thermal change, so a falling percentage in this graph in the period 1984–2008 (during which there was constant warming) suggests niche plasticity.

6 Supplementary tables

6.1 Negative correlations between the proportions of four broad taxonomic groups north of isotherms and mean latitude of isotherms 1954–2013.

Table 1a: Significance of negative correlations between the proportions of four broad taxonomic groups north of isotherms at 11°, 12° and 13°C and the mean latitude of the isotherms in the 12 five-year periods 1954–2013 during which there were periods of cooling and warming. Abundance data used for each taxonomic group were the means of the normalised values for the individual taxa. The *p*-values were calculated after correction for autocorrelation using the Chelton equation to re-estimate degrees of freedom. A significant negative correlation indicates niche plasticity in relation to thermal change.

	Isotherm at:			
	11°C	12°C	13°C	
Diatoms	0.0002	0.0003	0.0004	***
Dinoflagellates	0.0044	0.0173	0.0107	***
Metridia and *Pleuromamma*	0.0083	0.0157	0.0113	***
Calanus, *Euchaeta* and *Undeuchaeta*	0.4254	0.3139	0.6611	

* one isotherm $p < 0.05$; ** two isotherms $p < 0.05$; *** all isotherms $p < 0.05$

6.2 Negative correlations between the proportions of all taxa north of isotherms and mean latitude of isotherms 1954–2013.

Table 1b: Significance of negative correlations between the proportions of diatom, dinoflagellate and copepod taxa north of isotherms at 11°, 12° and 13°C and the mean latitude of the isotherms in the 12 five-year periods 1954–2013 (unless noted) during which there were periods of cooling and warming. The p-values were calculated after correction for autocorrelation using the Chelton equation to re-estimate degrees of freedom. A significant negative correlation indicates niche plasticity in relation to thermal change.

	11°C	12°C	13°C	
Diatoms				
Ditylum brightwellii †	0.5451	0.5795	0.7580	
Eucampia zodiacus	0.0002	0.0003	0.0009	***
Pseudo-nitzschia complex	0.0026	0.0060	0.0054	***
Pseudo-nitzschia seriata complex	0.0352	0.0274	0.0042	***
Proboscia indica	0.0212	0.0362	0.1067	**
Rhizosolenia hebetata semispina	0.0005	0.0010	0.0013	***
Rhizosolenia imbricata	0.0093	0.0042	0.0015	***
Rhizosolenia styliformis	0.0019	0.0017	0.0013	***
Skeletonema costatum	0.9904	0.6950	0.8516	
Thalassiosira spp.	0.0007	0.0006	0.0005	***
Thalassiothrix longissima	0.0019	0.0031	0.0028	***
Thalassionema nitzschioides	0.0034	0.0063	0.0039	***
Dinoflagellates				
Ceratium furca	0.0257	0.0762	0.0392	**
Ceratium fusus	0.0011	0.0018	0.0017	***
Ceratium lineatum	0.0258	0.0718	0.0691	*
Ceratium tripos	0.0032	0.0433	0.0506	**
Ceratium macroceros	0.0969	0.2918	0.1251	
Ceratium longipes	0.2784	0.3976	0.2569	
Ceratium minutum	0.0261	0.0187	0.0094	***
Ceratium hexacanthum	0.2484	0.2223	0.3887	
Dinophysis spp.	0.0041	0.0115	0.0130	***
Noctiluca scintillans ‡	0.2412	0.3725	0.4557	
Prorocentrum spp.	0.3920	0.4489	0.2962	
Protoperidinium spp.	0.0020	0.0034	0.0021	***
Copepods				
Calanus finmarchicus †	0.3425	0.0742	0.0819	
Calanus helgolandicus †	0.3783	0.3666	0.7731	
Euchaeta acuta	0.4583	0.4699	0.8121	
Euchaeta hebes	0.7357	0.7875	0.9678	
Undeuchaeta plumosa	0.3426	0.2671	0.1370	
Metridia longa	0.3754	0.8115	0.3755	
Metridia lucens	0.0245	0.0120	0.0047	***
Pleuromamma abdominalis	0.0170	0.0331	0.0354	***
Pleuromamma borealis	0.3211	0.4487	0.8408	
Pleuromamma gracilis	0.0134	0.0151	0.0124	***
Pleuromamma robusta	0.0304	0.0414	0.0327	***

* one isotherm $p < 0.05$; ** two isotherms $p < 0.05$; *** all isotherms $p < 0.05$
† 1959–1963 to 2009–2013; ‡ 1984–1988 to 2009–2013.

6.3 Correlations between raw and normalised populations of four broad taxonomic groups and mean SST for the warming period and for the 12 five-year periods 1954–2013.

Table 2a: Correlations between raw and normalised populations of four broad taxonomic groups and mean sea surface temperature for the period 1984–88 to 2004–2008 (a period of consistent warming) and 1954–58 to 2009–2013 (during which there were periods of warming and cooling). The raw population numbers were the sum of the values estimated by ordinary kriging at each 0.5° longitude and latitude position for each 5-year period for each group. The normalised data for each taxonomic group were the means of the normalised values for the individual taxa. The p-values were calculated after correction for autocorrelation using the Chelton equation to re-estimate degrees of freedom.

	1984–88 to 2004–2008			1954–58 to 2009–2013	
	t value	p value		t value	p value
Sum of raw populations					
Diatoms	1.1237	0.3260		0.6944	0.5180
Dinoflagellates	−2.6706	0.0264	*	−1.8182	0.0559
Metridia and *Pleuromamma*	−0.9342	0.1937		−0.8453	0.2202
Calanus, *Euchaeta* and *Undeuchaeta* †	0.1440	0.8890		−0.0009	0.4996
Mean of normalised populations					
Diatoms	1.6690	0.1807		0.8629	0.4279
Dinoflagellates	−1.7422	0.0683		−1.1547	0.1378
Metridia and *Pleuromamma*	1.4860	0.2200		−0.1595	0.4403
Calanus, *Euchaeta* and *Undeuchaeta* †	0.9647	0.3804		1.7125	0.1185

* $p < 0.05$

† 1959–1963 to 2009–2013 in columns 4 and 5.

6.4 Correlations between raw and normalised populations of all taxa and mean SST for the warming period and for the 12 five-year periods 1954–2013.

Table 2b: Correlations between populations of diatom, dinoflagellate and copepod taxa and mean sea surface temperature for the periods 1984–88 to 2004–2008, a period of consistent warming, and 1954–58 to 2009–2013 (unless noted), during which there were periods of warming and cooling. The population numbers were estimated by ordinary kriging at each 0.5° longitude and latitude position for each 5-year period. The p-values were calculated after correction for autocorrelation using the Chelton equation to re-estimate degrees of freedom.

	1984–88 to 2004–2008			1954–58 to 2009–2013		
	t value	p value		t value	p value	
Diatoms						
Ditylum brightwellii †	6.6281	0.0080	*	2.7185	0.0422	*
Eucampia zodiacus	0.2009	0.8487		0.6413	0.5320	
Pseudo-nitzschia complex	1.6536	0.1899		1.0223	0.3275	
Pseudo-nitzschia seriata complex	1.5610	0.2123		1.8850	0.0994	
Proboscia indica	−1.9381	0.0736		−0.7851	0.2241	
Rhizosolenia hebetata semispina	1.0024	0.3756		−0.3732	0.3578	
Rhizosolenia imbricata	1.3556	0.2567		0.8072	0.4412	
Rhizosolenia styliformis	−4.1833	0.0166	*	−1.5986	0.0733	
Skeletonema costatum	1.4776	0.2254		1.3971	0.1834	
Thalassiosira spp.	1.0907	0.3397		2.9954	0.0303	*
Thalassiothrix longissima	−1.3132	0.1261		−0.7101	0.2463	
Thalassionema nitzschioides	1.1231	0.3172		0.3144	0.7570	
Dinoflagellates						
Ceratium furca	−2.0123	0.0510		−1.8356	0.0463	*
Ceratium fusus	−4.2192	0.0099	*	−2.3967	0.0221	*
Ceratium lineatum	−5.5269	0.0055	*	−3.7881	0.0044	*
Ceratium tripos	−6.4263	0.0036	*	−1.8378	0.0474	*
Ceratium macroceros	−0.8749	0.2017		−0.5833	0.2850	
Ceratium longipes	−0.7850	0.2264		−1.1155	0.1433	
Ceratium minutum	0.6585	0.5345		0.4941	0.6270	
Ceratium hexacanthum	0.8292	0.4455		2.6654	0.0527	
Dinophysis spp.	−4.0609	0.0112	*	−1.7373	0.0528	
Noctiluca scintillans ‡	3.1979	0.0588		3.5963	0.0364	*
Prorocentrum spp.	−3.2105	0.0296	*	0.5348	0.5991	
Protoperidinium spp.	−2.7814	0.0252	*	−2.3582	0.0220	*
Copepods						
Calanus finmarchicus †	−2.2943	0.0428	*	−3.5204	0.0094	*
Calanus helgolandicus †	6.9201	0.0092	*	5.7153	0.0074	*
Euchaeta acuta	−0.3643	0.3625		−0.0312	0.4877	
Euchaeta hebes	3.8120	0.0314	*	6.1786	0.0040	*
Undeuchaeta plumosa	1.6137	0.1938		0.3063	0.7644	
Metridia longa	0.9450	0.3839		−1.0258	0.1663	
Metridia lucens	−2.4387	0.0448	*	−0.9529	0.1809	
Pleuromamma abdominalis	1.5265	0.2153		0.4494	0.6581	
Pleuromamma borealis	3.6056	0.0472	*	0.7609	0.4586	
Pleuromamma gracilis	3.5975	0.0454	*	2.4633	0.0421	*
Pleuromamma robusta	−3.3345	0.0200	*	−2.5383	0.0245	*

* $p < 0.05$; † 1959–1963 to 2009–2013; ‡ 1984–1988 to 2009–2013

6.5 Descriptive statistics of all taxa for the 12 five-year periods 1954–2013.

Table 3: Descriptive statistics of populations of diatom, dinoflagellate and copepod taxa for the 12 five-year periods 1954–2013 (unless noted) during which there were periods of cooling and warming. The population numbers here are the means of the values estimated by ordinary kriging at each 0.5° longitude and latitude position for each 5-year period.

	Population 1954–58 to 2009–2013			
	mean	SD	max	min
Diatoms				
Ditylum brightwellii †	0.1307	0.0715	0.299	0.0449
Eucampia zodiacus	0.055	0.0227	0.0923	0.0298
Pseudo-nitzschia complex	0.9588	0.3779	1.8297	0.5653
Pseudo-nitzschia seriata complex	0.8218	0.2854	1.3192	0.4137
Proboscia indica	0.3438	0.1881	0.821	0.1595
Rhizosolenia hebetata semispina	0.5862	0.1417	0.9843	0.4801
Rhizosolenia imbricata	0.6377	0.2714	1.1728	0.364
Rhizosolenia styliformis	0.7889	0.2702	1.5255	0.4863
Skeletonema costatum	0.1933	0.0988	0.3798	0.0867
Thalassiosira spp.	1.9102	0.3656	2.4654	1.3121
Thalassiothrix longissima	0.5123	0.3841	1.5868	0.211
Thalassionema nitzschioides	1.3269	0.4794	2.4307	0.8655
Dinoflagellates				
Ceratium furca	1.6862	0.5181	2.7296	0.832
Ceratium fusus	2.5022	0.6574	3.72	1.4981
Ceratium lineatum	0.6399	0.2109	0.9737	0.2551
Ceratium tripos	1.0716	0.3533	1.9737	0.5958
Ceratium macroceros	0.699	0.3994	1.6671	0.3288
Ceratium longipes	0.3721	0.2289	0.9342	0.0905
Ceratium minutum	0.0587	0.0324	0.1153	0.0118
Ceratium hexacanthum	0.1088	0.0657	0.2144	0.0253
Dinophysis spp.	0.2946	0.0898	0.451	0.1323
Noctiluca scintillans ‡	0.2157	0.1014	0.3512	0.088
Prorocentrum spp.	0.1333	0.0654	0.2215	0.0147
Protoperidinium spp.	0.6387	0.2076	1.1074	0.2964
Copepods				
Calanus finmarchicus †	0.573	0.1721	0.8476	0.3258
Calanus helgolandicus †	0.5865	0.1266	0.771	0.4262
Euchaeta acuta	0.0121	0.0044	0.0183	0.0043
Euchaeta hebes	0.0311	0.0208	0.0701	0.0074
Undeuchaeta plumosa	0.0109	0.0046	0.0175	0.0014
Metridia longa	0.0045	0.0046	0.0138	0
Metridia lucens	0.4789	0.1167	0.7872	0.3667
Pleuromamma abdominalis	0.0132	0.0094	0.0382	0.0011
Pleuromamma borealis	0.0427	0.0224	0.0859	0.0111
Pleuromamma gracilis	0.0468	0.0203	0.0887	0.0228
Pleuromamma robusta	0.1041	0.0512	0.1676	0.0259

† 1959–1963 to 2009–2013; ‡ 1984–1988 to 2009–2013

6.6 Northerly movement of all taxa in the cooling period and the warming period, changes in the proportion of each taxon north of isotherms in the warming period and significant negative correlations between the proportion north of isotherms and the mean latitude of the isotherms in the 12 five-year periods 1954–2013.

Table 4: a, Northerly movement of range median latitude of diatom, dinoflagellate and copepod taxa in the cooling period of 1959–1963 to 1984–1988 and the warming period of 1984–1988 to 2004–2008. **b,** Changes in the proportion of each taxon north of isotherms at 11°, 12° and 13°C in the warming period of 1984–1988 to 2004–2008. **c,** Significant negative correlations ($p < 0.05$ indicated by asterisks) between the proportion of each taxon north of isotherms at 11°, 12° and 13°C and the mean latitude of the isotherms in the 12 five-year periods 1954–2013 during which there were periods of warming and cooling. A significant negative correlation indicates niche plasticity in relation to thermal change (please see Supplementary Table 1b).

	**a** Movement north (km) of range median latitude		**b** Change in proportion north of isotherm in warming period			**c** Negative correlation proportion north and isotherm latitudes
	cooling period 59–63 to 84–88	warming period 84–88 to 04–08	11°C	12°C	13°C	54–58 to 09–13
Eucampia zodiacus (Dia)	56.20	−224.64	−27.59	−23.80	−16.69	***
Pleuromamma gracilis (Cop)	248.61	−196.79	−20.19	−21.73	−21.41	***
Euchaeta hebes (Cop)	296.32	−100.86	−11.30	−14.60	−15.80	
Dinophysis spp. (Din)	83.70	−89.27	−20.71	−16.81	−13.23	***
Pleuromamma abdominalis (Cop)	105.99	−70.40	−12.69	−17.32	−20.07	***
Metridia lucens (Cop)	−87.49	−38.32	−14.09	−14.76	−13.18	***
Pleuromamma robusta (Cop)	−71.12	3.45	−12.32	−12.82	−11.75	***
Thalassiosira spp. (Dia)	−135.22	34.81	−15.66	−13.04	−10.40	***
Undeuchaeta plumosa (Cop)	37.67	57.78	−2.95	−4.83	−9.57	
Rhizosolenia styliformis (Dia)	−137.41	65.72	−14.79	−13.88	−11.60	***
Rhizosolenia imbricata (Dia)	−21.88	65.82	−15.19	−12.24	−9.62	***
Pleuromamma borealis (Cop)	224.49	72.57	−15.02	−16.24	−13.52	
Thalassiothrix longissima (Dia)	−147.47	79.34	−17.45	−14.22	−11.65	***
Pseudo-nitzschia seriata complex (Dia)	−136.93	83.11	−13.31	−10.80	−8.72	***
Protoperidinium spp. (Din)	−59.84	87.79	−13.98	−10.08	−7.59	***
Rhizosolenia hebetata semispina (Dia)	−61.16	97.59	−12.50	−11.85	−10.65	***
Thalassionema nitzschioides (Dia)	−98.02	99.99	−13.49	−10.92	−8.29	***
Ceratium fusus (Din)	−104.42	103.66	−13.69	−11.18	−8.23	***
Ceratium minutum (Din)	116.35	119.54	−10.85	−10.73	−9.29	***
Calanus finmarchicus (Cop) †	46.11	137.62	5.27	7.28	4.23	
Ceratium tripos (Din)	−76.19	149.08	−10.42	−4.68	−2.93	**
Skeletonema costatum (Dia)	11.90	157.61	−8.22	−1.20	0.02	
Pseudo-nitzschia complex (Dia)	−56.41	159.63	−10.22	−8.06	−6.68	***
Ceratium lineatum (Din)	−165.74	159.97	−10.77	−6.34	−2.71	*
Proboscia indica (Dia)	−66.26	169.63	−13.68	−9.70	−4.99	**
Euchaeta acuta (Cop)	−4.52	181.51	−1.28	−0.43	−3.73	
Noctiluca scintillans (Din) ‡	NA	211.33	−10.07	−8.09	−5.14	
Ceratium furca (Din)	−101.53	238.79	−9.83	−7.26	−5.41	**
Calanus helgolandicus (Cop) †	12.36	255.30	−5.78	−4.55	−5.87	
Prorocentrum spp. (Din)	−353.82	281.29	−5.25	−3.09	−3.88	
Ceratium longipes (Din)	−1.94	335.85	−4.46	−2.56	−1.98	
Ditylum brightwellii (Dia) †	−420.56	372.59	2.19	6.30	9.45	
Ceratium macroceros (Din)	−363.97	406.08	−1.99	2.33	0.73	
Ceratium hexacanthum (Din)	−74.07	572.92	18.54	20.45	16.00	
Metridia longa (Cop)	−492.04	680.17	21.85	27.37	13.09	

Data available from † 1959–1963 to 2009–2013; ‡ 1984–1988 to 2009–2013.

6.7 Northerly movement of four broad taxonomic groups and three isotherms in the cooling and warming periods.

Table 5: Northerly movement of four broad taxonomic groups and three isotherms in the periods 1959–1963 to 1984–1988, a period of cooling, and 1984–1988 to 2004–2008, a period of warming. The data for each taxonomic group were the means of the raw values for the individual taxa estimated by ordinary kriging at each 0.5° longitude and latitude position for each 5-year period. DIA: Diatoms; DIN: Dinoflagellates; MP: *Metridia* and *Pleuromamma*; CEU: *Calanus*, *Euchaeta* and *Undeuchaeta*.

Group	Cooling period 1959–1963 to 1984–1988	Warming period 1984–1988 to 2004–2008
DIA	−84km	92km
DIN	−111km	135km
MP	−1km	−90km
CEU	−29km	84km
Isotherm		
11°C	−137km	377km
12°C	−88km	315km
13°C	−39km	261km

6.8 Group median latitude of four broad taxonomic groups, mean isotherm latitude and mean SST in the 12 five-year periods 1954–2013.

Table 6: Group median latitude, mean isotherm latitude and mean SST in the 12 five-year periods 1954–2013 during which there were periods of warming and cooling: **a,** Median latitude of four broad taxonomic groups (the data for each taxonomic group were the means of the raw values for the individual taxa estimated by ordinary kriging at each 0.5° longitude and latitude position), **b,** latitide of mean isotherm positions in the geographic range 45–64°N, 20°W–8°E and **c,** mean SST in the same geographic range. **DIA:** Diatoms; **DIN:** Dinoflagellates; **MP:** *Metridia* and *Pleuromamma*; **CEU:** *Calanus*, *Euchaeta* and *Undeuchaeta*.

Five-year	**a** Group median lat. (°N)				**b** Mean iso. lat. (°N)			**c** Mean SST (°C)
period	DIA	DIN	MP	CEU	11°C	12°C	13°C	
1954–1958	55.50	56.18	54.77	56.09	54.93	52.23	49.72	11.47
1959–1963	55.59	56.04	55.04	56.74	55.23	52.13	49.43	11.52
1964–1968	54.66	54.47	54.94	55.89	54.92	52.00	49.46	11.43
1969–1973	54.84	55.05	54.07	56.87	54.70	51.94	49.45	11.36
1974–1978	54.48	55.23	53.54	55.91	54.86	51.97	49.32	11.32
1979–1983	55.33	55.23	54.07	55.09	54.54	51.91	49.49	11.24
1984–1988	54.83	55.04	54.95	56.48	54.00	51.34	49.08	11.21
1989–1993	55.31	55.72	55.96	56.62	55.32	52.41	50.00	11.52
1994–1998	55.47	55.97	54.89	56.68	55.64	52.81	50.32	11.62
1999–2003	55.08	55.69	54.83	56.95	56.89	53.64	50.96	11.93
2004–2008	55.66	56.26	54.14	57.24	57.40	54.18	51.43	12.09
2009–2013	55.68	55.56	55.56	56.79	56.26	52.86	50.24	11.79
